# Limitations of bacterial culture, viral PCR, and tulathromycin susceptibility from upper respiratory tract samples in predicting clinical outcome of tulathromycin control or treatment of bovine respiratory disease in high-risk feeder heifers

**DOI:** 10.1371/journal.pone.0247213

**Published:** 2022-02-10

**Authors:** Jeffrey J. Sarchet, John P. Pollreisz, David T. Bechtol, Mitchell R. Blanding, Roger L. Saltman, Patrick C. Taube

**Affiliations:** 1 Zoetis, Parsippany-Troy Hills, New Jersey, United States of America; 2 Agri Research Center, Canyon, Texas, United States of America; University of Lincoln, UNITED KINGDOM

## Abstract

A cross-sectional prospective cohort study including 1026 heifers administered tulathromycin due to high risk of clinical signs of bovine respiratory disease (BRD), measured poor association between BRD clinical outcomes and results of bacterial culture and tulathromycin susceptibility from BRD isolates of deep nasopharyngeal swabs (DNS) and adequate association with viral polymerase chain reaction (PCR) results from nasal swabs. Isolation rates from DNS collected on day-0 and at 1^st^ BRD-treatment respectively were: *Mannheimia haemolytica* (10.9% & 34.1%); *Pasteurella multocida* (10.4% & 7.4%); *Mycoplasma bovis* (1.0% & 36.6%); and *Histophilus somni* (0.7% & 6.3%). Prevalence of BRD viral nucleic acid on nasal swabs collected exclusively at 1^st^ BRD-treatment were: bovine parainfluenza virus type-3 (bPIV-3) 34.1%; bovine viral diarrhea virus (BVDV) 26.3%; bovine herpes virus type-1 (BHV-1) 10.8%; and bovine respiratory syncytial virus (BRSV) 54.1%. Increased relative risk, at 95% confidence intervals, of 1^st^ BRD-treatment failure was associated with positive viral PCR results: BVDV 1.39 (1.17–1.66), bPIV-3 1.26 (1.06–1.51), BHV-1 1.52 (1.25–1.83), and BRSV 1.35 (1.11–1.63) from nasal swabs collected at 1^st^ BRD-treatment and culture of *M*. *haemolytica* 1.23 (1.00–1.51) from DNS collected at day-0. However, in this population of high-risk feeder heifers, the predictive values of susceptible and resistant isolates had inadequate association with BRD clinical outcome. These results indicate, that using tulathromycin susceptibility testing of isolates of *M*. *haemolytica* or *P*. *multocida* from DNS collected on arrival or at 1^st^ BRD-treatment to evaluate tulathromycin clinical efficacy, is unreliable.

## Introduction

For many decades, Bovine Respiratory Disease (BRD) has been a challenging problem for veterinarians, cattle, and producers due to complex interactions that exist with various bacterial and viral pathogens, inconsistent and unpredictable environmental and management risk factors, and variable immune capabilities of cattle that alter the outcome of BRD and make diagnosis and treatment difficult [1–4]. Some [5–8] consider immune compromise to be as important in the disease process of BRD as the various infectious pathogens involved. Together, these interactions as well as limitations of sampling lungs of live cattle, contribute to the lack of a “gold standard” for definitive antemortem diagnosis of BRD [9]. In 2021, the most commonly applied method for diagnosing BRD in the cattle industry of North America is the use of clinical signs however, variations in ability to efficiently observe clinical signs of individual animals in a group along with inability to observe subclinical signs of BRD, makes observation of clinical signs an imperfect standard [9, 10]. Bacterial culture and antimicrobial susceptibility testing are commonly used to guide the selection or evaluate the efficacy of BRD antimicrobial treatments [7–9, 11]. However, applying these methods to complex disease processes like BRD, may lead to erroneous conclusions, similar to what has been found in other diseases of animals and humans with polymicrobial infections [12–15]. Use of less effective or ineffective antimicrobials can lead to animal welfare issues as well as increased prevalence of antimicrobial resistance.

BRD is a complex disease process involving variable bacterial and viral pathogens as well as a variety of physical and physiological stressors that predispose cattle to pneumonia [3, 10, 16, 17]. Infectious pathogens associated with BRD are ubiquitous among cattle populations and major bacterial respiratory pathogens are typically commensal in clinically normal feedlot cattle therefore, one or a combination of stressors are typically necessary to initiate BRD [4, 6–8]. Clinical BRD is a product of the effects of factors causing immunosuppression, which allows colonization of the respiratory tract by opportunistic pathogens. Some management factors that have been associated with increased risk of BRD are transportation, time without feed and water, comingling, climate, nutritional status, and introductory diets [18–20].

PCR is gaining favor as a diagnostic tool because of the ease of application and recent advances in technology that provide relatively inexpensive and rapid test results. These benefits can be mitigated in the realm of BRD diagnostics by drawbacks with interpretation and validation of results. One of the challenges with diagnosis of BRD is obtaining samples from the lower respiratory tract in live cattle, because it involves more invasive, time consuming, and more complex procedures that are less routinely performed in the field and can have unpredictable inherent risks to animals sampled. Consequently, sampling the upper respiratory tract is more frequently used for BRD diagnostic testing [11]. Characteristics of diagnostic tests for BRD from samples taken via the upper respiratory tract have been investigated by several different researchers with inconsistent conclusions [21–27]. However, antemortem diagnostic methods of sampling the lower respiratory tract have shown superior association with clinical signs of BRD [28].

A key parameter that guides decisions regarding antimicrobial therapy is the “clinical breakpoint” or veterinary specific interpretive criteria which are the antimicrobial concentrations used to define isolates as susceptible (S), intermediate (I), or resistant (R) [29, 30]. However, some investigators have suggested that this bioassay is fundamentally flawed because it is based largely on in vitro efficacy, and often fails to correlate with patient outcome [13–15, 31–35].

There is pervasive ambiguity about the interpretation of susceptible, intermediate, and resistant isolates in human and veterinary medicine and definitions in published manuscripts are inconsistent. Confusion can occur because susceptibility categories can refer to direct interaction between antibacterial agent and organism or likelihood that the patient will respond to antimicrobial treatment. A more encompassing set of definitions is provided by Turnidge [29] and the Clinical and Laboratory Standards Institute (CLSI) [30]. Susceptible category implies that isolates are inhibited by usually achievable concentrations of an antimicrobial agent when the recommended dosage (dosage regimen) is used for that site of infection. Intermediate category includes isolates with antimicrobial agent minimal inhibitory concentrations (MIC)s that approach usually attainable blood and tissue levels and for which response rates may be lower than those for susceptible isolates. The intermediate category implies clinical efficacy in body sites where the drugs are physiologically concentrated (quinolones and -lactams in urine) or when a higher than-normal dosage of a drug can be used (lactams). Intermediate category also includes a buffer zone which should prevent small, uncontrolled technical factors from causing major discrepancies in interpretations, especially for drugs with narrow pharmacotoxicity margins. Resistant category implies that isolates are not inhibited by usually achievable concentrations of the antimicrobial agent with normal dosage schedules and/or demonstrate MIC/zone diameters that fall in the range where specific microbial resistance mechanisms are likely and that clinical efficacy against the isolate has not been reliably shown in treatment studies [29, 30]. In vitro definitions indicate that with susceptible isolates, growth of the bacterial strain is inhibited by an antibacterial agent concentration in the range found for wild-type strains. Resistant isolates, indicate that growth of the bacterial strain is inhibited by an antibacterial agent concentration higher than the range seen for wild-type strains; and wild type strains that harbor no acquired resistance mechanism to the antimicrobial under question, specifically no resistance attributable to (1) mutation, (2) acquisition of foreign DNA, (3) up-regulation of an efflux pump, (4) up-regulation of target production, or (5) any combination of these [29, 30]. Clinical definitions signify that susceptible bacterial strains are inhibited by a concentration of an antibacterial agent that is associated with a high likelihood of therapeutic success; intermediate bacterial isolates are inhibited by a concentration of an antibacterial agent that is associated with an uncertain therapeutic effect; and resistant bacterial isolates are inhibited by concentrations of an antibacterial agent that is associated with a high likelihood of therapeutic failure [30]. However, “high likelihood” is subjective and not clearly defined. The primary objective of this study is to use the clinical definition to correlate MIC of tulathromycin with clinical outcomes of BRD in DNS samples collected from feeder heifers at increased risk of developing BRD, to provide practitioners with better information to make interpretations for evaluating tulathromycin for the treatment or control of BRD.

The objective of MIC’s is the establishment of values to which other parameters, such as MIC distributions, phenotype and genotype resistance markers, pharmacodynamic (PD) end points, animal PD model studies, and clinical study outcomes, can be reliably integrated to predict improved clinical outcomes of antimicrobial therapies. This requires integration of robust epidemiological, PD and clinical data so that MICs have a reasonable level of reproducibility, however it is frequently quoted that the “error” associated with measuring MIC is “plus or minus one two-fold dilution” [30]. While this can work as a rule of thumb, results from so-called “tier 2 studies” described by the CLSI for establishing quality control ranges show that precision of MIC measurements can be less than or greater than this, depending on the organism-antibacterial combination [30]. Ultimately, veterinary specific interpretive criteria are established by the Veterinary Antimicrobial Susceptibility Testing Subcommittee of the CLSI using the best information available [30]. However, even when appropriate standards are used, inadequacies of antimicrobial susceptibility methods, can nonetheless limit association of clinical outcomes with antimicrobial therapies.

When evaluating a patient that has failed to respond to therapy, one must consider many factors that contribute to antibiotic failure. Doern [31] report satisfactory clinical predictive value (90–95%) for susceptibility tests in immunocompetent patients with monomicrobic bacterial infections when treated with a single antimicrobial agent administered parenterally where the penetration of the drug to the site of infection is predictable. Since BRD is not typically a monomicrobic infection and cattle with BRD are often immunocompromised, it would be unreasonable to expect 90–95% clinical predictive value with BRD antimicrobial susceptibility results. The 90/60 rule was derived from observations in human medicine that, in general, bacteria treated with antimicrobials to which the strain is sensitive will have a favorable therapeutic response in approximately 90% of the patients [31, 32]. On the other hand, when bacteria are resistant to the antimicrobial administered, despite the susceptibility result, approximately 60% of patients will respond to therapy. In veterinary medicine, we have little data to confirm or challenge the 90/60 rule and there is a lack of published information that superior clinical outcomes for BRD can be achieved by applying susceptibility results. So, the question remains: How well do tulathromycin susceptibility test results correlate with BRD clinical outcomes? Variable polymicrobial etiologies; correlation of pathogen with pathology or disease; classification of pathogen virulence; and unpredictable host-pathogen interactions are challenges that many BRD diagnostic test methods fail to account for [9]. These inadequacies, cause some to question the utility of antimicrobial susceptibility methods for selecting or evaluating BRD antimicrobial therapies.

Tulathromycin is the most commonly used antimicrobial for BRD and evidence from multiple meta-analyses, indicate tulathromycin as being one of, if not the most effective antimicrobial for BRD metaphylaxis and treatment [36–38]. Although the incidence of tulathromycin resistance has been reported as low, diagnostic labs do report resistant isolates of tulathromycin which can lead to using potentially less effective antimicrobial agents [39]. The primary objective of this study was to evaluate the association between tulathromycin susceptibility test results of *Mannheimia haemolytica (M*. *haemolytica)*, and *Pasteurella multocida (P*. *multocida)*, isolates derived from deep nasopharyngeal swabs (DNS) with tulathromycin metaphylaxis or 1^st^ BRD-treatment outcome in comingled, transported high-risk feeder heifers, during the first 42 days on feed, to assist practitioners with interpretation of DNS results. A secondary objective of the study was to assess the association of multiplex viral PCR results, including bovine viral diarrhea virus (BVDV), bovine parainfluenza-3 virus (bPIV-3), bovine herpes virus-1 (BHV-1), and bovine respiratory syncytial virus (BRSV), from nasal swabs taken at 1^st^ BRD-treatment with clinical outcome after the 1^st^ BRD-treatment, to facilitate practitioners with interpretation of PCR results from cattle that are at high risk of developing BRD and have received a modified live viral vaccine.

## Materials and methods

### Study population & research facilities

This prospective cross-sectional cohort study observed eleven truckloads (94–116/load) of heifers procured primarily from auction facilities in Alabama, but also Kentucky, and South-Central Texas, during the first 42 days on feed, following tulathromycin metaphylaxis and first treatment for BRD. Association of bacterial culture, tulathromycin susceptibility testing, and viral PCR was compared with tulathromycin metaphylaxis (day-0) and 1^st^ BRD-treatment outcomes. This high-risk BRD model was based on previous history of procuring heifers of similar age (6–9 months), weight (205–250 kg.), and origin from these livestock auctions. The goal was to purchase animals at “high-risk” (> 40%) of developing BRD within 30 days of arrival in the feedlot. This study was executed during springtime at a research feedlot located in the Texas Panhandle with management, facilities, and environment typical of most cattle feeding operations located in the central plains of the United States and Canada.

Mean body weight of 1031 enrolled heifers on day-0 of the study was 226 kg (498 lbs.), ranging between 144 kg (317 lbs.) and 292 kg (642 lbs.). Heifers from each truckload were allocated between March 5, 2015 and March 28, 2015, to pens of 20 animals with 90 to 120 square feet of pen space and 18 to 24 inches of bunk space. All cattle were housed in dirt floor pens with steel post and cable fencing with concrete fence-line bunks with aprons and float activated water troughs. Cattle were fed a total mixed starter ration consisting of 47.5% steam flaked corn, 23% ground alfalfa hay, 5% protein/mineral/vitamin supplement, 5% molasses, 4.5% cotton seed meal, and 15% cotton seed hulls. Feed was delivered with a mixer truck with load cells and was fed twice a day with amounts adjusted daily, so the cattle consumed all the feed by the next morning. Animals with signs of lameness or disease other than BRD which the investigating veterinarian expected would prevent the heifer from finishing the study were excluded before enrollment. Heifers (n = 1031) were enrolled (day-0) within 36 hours of arrival. The investigating veterinarian walked through each pen and scored each individual animal in each pen at approximately the same time each day for a period of 42 days using a clinical appearance score (CAS) system (S1 Appendix). Identification of all animals with a CAS>0 was recorded daily on written forms and observations on the last truckload of cattle ended on May 9, 2015.

Certified scales were calibrated each day before enrollment of cattle. Laboratory personnel were masked to the origin of samples and research personnel were masked to the laboratory results until the completion of the study. Institutional IACUC ethics committee reviewed and approved the study protocol prior to implementation. No animals were euthanized, and protocols were in place to promote animal wellness and mitigate suffering. Any animals identified with critically severe clinical signs (CAS 3), were to be given immediate emergency therapy and removed from the study. Any animal found to be moribund (CAS 4), was to be removed from the study and humanely euthanized per the American Veterinary Medical Association “Guidelines for Euthanasia of Animals,” 2013 Edition [40]. Products in the arrival and treatment protocols were administered per Beef Quality Assurance Guidelines (BQA) [41]. (S2 Appendix).

**Fig 1:**
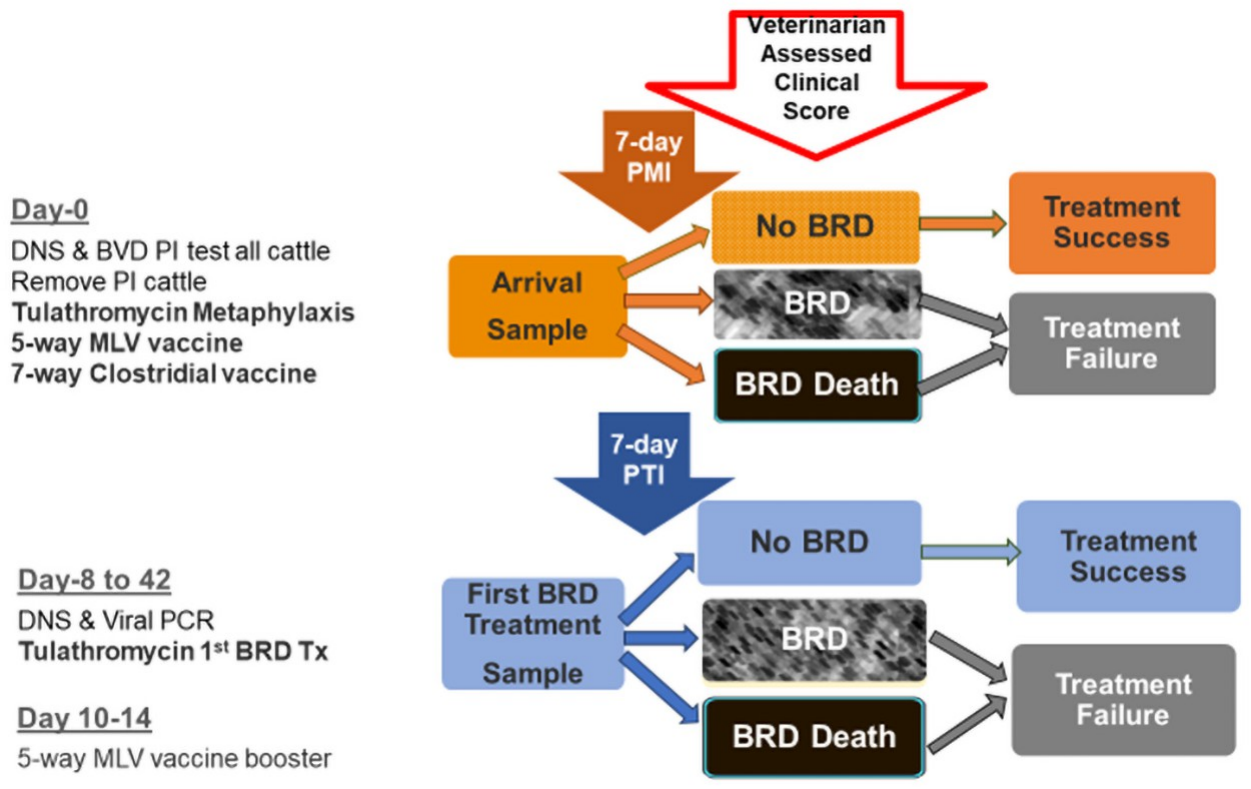
Study timeline. Nasopharyngeal swab, tulathromycin susceptibility, *M*. *haemolytica*, *P*. *multocida*, bovine respiratory disease, bacterial culture, viral PCR.

Animals were not eligible for additional treatment of BRD for a period of 7 days after 1^st^ BRD-treatment administration of tulathromycin (7-day PTI) unless, per protocol, animals that scored a CAS≥3 were given emergency treatment or were euthanized. All animals that died during the study were necropsied by the investigating veterinarian and lung samples were sent for bacterial culture, tulathromycin susceptibility, and further testing such as histopathology, immunohistochemistry, PCR, and/or viral isolation, was performed, only if deemed necessary by the investigating veterinarian, to determine a definitive diagnosis for death. A timeline of the study activities is illustrated in Fig 1.

### Sample collection & laboratory methods

After wiping the external nares with a clean paper towel to remove external debris, a sterile Jorgensen Labs double guarded culture swab was inserted into the ventral nasal meatus until the pharyngeal mucosa was contacted or a distance from the nares to the medial canthus of the eye was reached. Day-0 and 1^st^ BRD-treatment DNS specimens were labeled with the study number, date of collection, sample type, and animal identification and then shipped to an accredited research laboratory (Microbial Research Incorporated, Fort Collins, Colorado), for culture and identification of *Mannheimia haemolytica*, *Pasteurella multocida*, *Mycoplasma bovis (M*. *bovis)* and *Histophilus somni (H*. *somni)*. DNS samples were shipped in Amies transport medium with charcoal and packed with ice packs via overnight courier, except samples collected on Saturday and Sunday which were held at 2-8°C and shipped on Monday. Samples were processed on the date of receipt in the lab, except samples received on Saturday which were held at 2-8°C until the following business day. All samples were received at the lab in suitable condition and specimens were streaked for isolation on 5% sheep blood agar plates (BA) and modified Hayflick’s agar (HFA). The BA plates were incubated in 5 ±2% CO_2_ at 36 ±2°C overnight and the HFA plates were incubated up to 7 days at 36±2°C in 5±2% CO_2_. The incubated BA plates were observed for the presence of presumptive *H*. *somni*, *M*. *haemolytica*, and *P*. *multocida* colonies. HFA agar plates were observed for typical *Mycoplasma bovis (M*. *bovis)* colonies. Each colony with a presumptive identity of *H*. *somni*, *P*. *multocida*, or *M*. *haemolytica* was identified by Maldi Biotyper. Presumptive *M*. *bovis* colonies were purified by two serial passages on HFA. Presumptive colonies were then dienes stained and tested for inhibition by digitonin to identify the isolates as *Mycoplasma* species. Speciation of *Mycoplasma* isolates was performed using a validated PCR procedure to confirm an identification of *Mycoplasma bovis*.

Tulathromycin susceptibility, using minimal inhibitory concentration (MIC) broth microdilution technique, was determined on *M*. *haemolytica* and *P*. *multocida* isolates using one representative colony from each sample. Isolates were kept frozen at 2–8°C until tulathromycin MIC values for *M*. *haemolytica* and *P*. *multocida* were determined using plates prepared by the research laboratory on May 29, 2015 which contained doubling dilution concentrations of tulathromycin from 0.12–64 ug/mL. Positive and negative growth control wells were included for each dilution series. MIC tests were performed using Clinical & Laboratory Standards Institute (CLSI) procedures [30]. Cation adjusted Mueller Hinton broth (MHB) was used for *P*. *multocida* and *M*. *haemolytica* isolates. Plates were incubated aerobically at 36±2°C for 19.5 hours. The MHB quality control organisms, *Enterococcus faecalis* and *Staphylococcus aureus*, tested on each testing date, were incubated aerobically at 36±2°C for 19.0–19.5 hours. Only one isolate was tested from each sample unless presumptive identification of the isolate was not confirmed.

### 1^st^ BRD-treatment sampling

Animals were not eligible for 1^st^ BRD-treatment until the eighth day (7-day PMI) after metaphylactic tulathromycin administration. Starting on day-8, animals with a CAS-1 and a rectal temperature >39.7°C or a CAS ≥2 (regardless of rectal temperature) were eligible to receive 1^st^ BRD-treatment and were classified as treatment failures from the metaphylaxis tulathromycin administration. At 1^st^ BRD-treatment, a second DNS for culture and tulathromycin sensitivity testing along with a nasal swab for multiplex viral PCR was collected and tulathromycin was administered at label dosage with the heifer classified as a day-0 treatment failure. DNS samples were collected using the same method as day-0 and a nasal swab was collected from the nares after cleaning the external nares with a clean paper towel then swabbing the nares with a Culturette EZ^™^ swab. Following collection, nasal swabs were stored in BD Vacutainer SST venous blood collection tubes, labeled refrigerated and shipped with ice packs overnight to Texas A&M Diagnostic Laboratory, Amarillo, Texas for multiplex PCR testing.

Upon arrival at the laboratory, dry nasal swabs were suspended in minimal essential medium and then frozen until testing was performed as described. Nucleic acid was purified from the submitted swab sample using the MagMAX-96 Viral RNA isolation kit from ThermoFisher. Briefly, the swab was moistened in ~700uL 1X phosphate buffered saline, pH 8.0 and ~150uL utilized for nucleic acid extraction. Sample was combined with 20uL of magnetic bead mix 10uL lysis binding enhancer and 10uL RNA binding beads and 400uL lysis binding solution 200uL lysis binding concentrate, 1uL carrier RNA (1ug/ul), 1uL XIPC RNA (at 10,000 copies/uL), and 200uL 100% isopropanol in a 96-well deep-well plate which was labeled as the sample plate. Nucleic acid extraction was performed using a KingFisher 96 automated particular processor. The following plates were added to the KingFisher 96 for extraction: sample plate, wash solution 1 plate (300uL/well), wash solution 2 plate (300uL/well) and elution buffer plate (90uL/well). Following nucleic acid extraction, eluted nucleic acid was kept refrigerated prior to PCR setup. BVD, BRSV and bPIV-3 were screened via multiplex PCR, utilizing the ThermoFisher PathID Multiplex One Step RT-PCR kit according to the manufacturer’s instructions, along with primers and probes for detection of BVD, BRSV, bPVI-3 and XIPC (an exogenous internal control). Each reaction contained the following: 12.5uL 2X Multiplex RT-PCR buffer, 2.5uL 10X multiplex enzyme mix, 1uL 25X primer-probe mix (containing all oligonucleotides for detection of all four targets) and 1uL nuclease free water; 8uL of extracted nucleic acid was added to 17uL of MasterMix for a total 25uL reaction volume. RT-qPCR was performed using the Applied Biosystems 7500Fast instrument. Cycling parameters were as follows: 48°C for 10min (1 cycle), 95°C for 10min (1 cycle), and 40 cycles at 95°C for 15sec and 55°C for 45sec. Samples with a quantification cycle (Cq) ≤ 37.0 were considered positive for the above-mentioned targets. Detection of IBR was assessed in a separate PCR using the PathID qPCR MasterMix from ThermoFisher, along with primers and probes for the detection of IBR and XIPC. Each reaction contained the following: 12.5uL 2X PathID qPCR buffer, 1uL 25X primer-probe mix (containing the oligonucleotides for the detection of IBR and XIPC) and 3.5uL nuclease free water; 8uL of extracted nucleic acid was added to 17uL of MasterMix for a total 25uL reaction volume. Cycling parameters and quantification cycle cutoff were the same as for the multiplex assay above.

### Statistical analysis

Sample number of ≥45 tulathromycin resistant isolates of *M*. *haemolytica* estimation was based on 1000 heifers sampled on day-0 with a 30% incidence of *M*. *haemolytica* isolation and 1% incidence of tulathromycin resistance (3 isolates) and 400 heifers sampled at 1^st^ BRD-treatment with an incidence of 30% *M*. *haemolytica* isolation and 35% incidence of tulathromycin resistance (42 isolates). Adjustment of the 90/60 rule [31] was made to assume that >75% of susceptible isolates would have a favorable clinical outcome when tulathromycin was administered at label dosage and >50% of resistant isolates would fail to respond to tulathromycin therapy, due to increased prevalence of polymicrobial infections and immunocompromised cattle commonly found with BRD. Due to low or no tulathromycin resistance reported in the literature [39], the limiting factor of this study was expected to be the number of tulathromycin resistant isolates, therefore the protocol was designed to maximize the number of resistant isolates cultured by using a source of high-risk feeder cattle with a suspected history of tulathromycin resistance. Using tulathromycin for metaphylaxis and 1^st^ BRD-treatment was not to measure efficacy of the protocol but to limit potential interactions of antimicrobial treatments, increase the probability of tulathromycin resistant isolates and simplify statistical analysis for association of a single antimicrobial agent, (tulathromycin) with BRD clinical outcomes.

Randomization of individual cattle or lots of cattle was not necessary because all cattle received the same arrival protocol (S2 Appendix), (administered by individual body weight and label instructions), were observed and given a CAS by the same investigating veterinarian, and all samples were tested at the same laboratories. The purpose of the study was not to measure efficacy of tulathromycin protocols but rather measure statistical parameters of these tulathromycin susceptibility, bacterial isolation, and viral PCR diagnostic methods relative to clinical outcome.

Written forms were submitted to the statistician for verification and transfer to the software program, (SAS) for data analysis. Data was transferred from written forms, validated, stored, and analyzed in a centralized data management system; SAS version 9.3. Data was summarized in contingency tables and analyzed for Sensitivity (Se), Specificity (Sp), Positive Predictive Value (PPV), Negative Predictive Value (NPV), and Relative Risk of Treatment Failure (RRTF). Clinical outcome was observed for 42 days on each cohort of cattle and defined as ***Treatment Success*** if the animal did not have clinical signs of BRD (CAS-0) during the 42-day observation period, according to the CAS assessed daily by the investigating veterinarian. ***Treatment Failure*** was defined as an animal with clinical signs of BRD (CAS≥1) assessed by the investigating veterinarian using the CAS any time after the 7-day PMI/PTI or death due to BRD. Animals classified as treatment failures were given additional antibiotic treatment according to the protocol or euthanized (CAS≥3).

For analysis of Se, Sp, PPV, NPV, and RRTF for bacterial culture and multiplex PCR testing (S3 Appendix), ***True Positive*** was defined as animals with BRD according to positive bacterial culture or viral PCR (test +) and classification as treatment failures (died or needed further treatment per CAS) following the 7-day PMI or PTI. ***True Negative*** result for the same statistical tests was defined as animals negative for BRD pathogens, or viral PCR (test -) that were classified as treatment successes (needing no further treatment per CAS) during the 42-day study. ***False Positive*** results were defined as animals identified with BRD bacterial and/or viral pathogens (test +) that were subsequently classified as treatment successes at day-42 and ***False Negative*** results were defined as animals without evidence of BRD pathogens (test -) but classified as treatment failures following the 7-day PMI or PTI.

For analysis of Se, Sp, PPV, and NPV of tulathromycin susceptibility, CLSI established tulathromycin veterinary specific interpretive criteria^30^ for *M*. *haemolytica* and *P*. *multocida* were used to establish susceptible (≤ 16 ug/ml), intermediate (32 ug/ml), and resistant (≥ 64 ug/ml) isolates as classified in S4 Appendix. ***True Positive*** results were defined as cattle with non-resistant (MIC < 64ug/ml) *M*. *haemolytica* or *P*. *multocida* isolates that were classified as treatment successes following no subsequent identification of clinical signs of BRD by the investigating veterinarian during the 42-day study. ***True Negative*** tulathromycin susceptibility results were defined as any cattle with resistant (MIC ≥ 64ug/ml) *M*. *haemolytica* or *P*. *multocida* isolates classified as treatment failures after reoccurrence of clinical signs of BRD following the 7-day PMI/PTI or death due to BRD subsequent to tulathromycin treatment. ***False Positive*** tulathromycin susceptibility results were defined as all cattle with non-resistant (MIC < 64ug/ml) *M*. *haemolytica* or *P*. *multocida* isolates that were classified as treatment failures after identification of clinical signs of BRD by the investigating veterinarian following the 7-day PMI/PTI. ***False Negative*** tulathromycin susceptibility results were defined as any cattle with resistant (MIC ≥ 64ug/ml) *M*. *haemolytica* or *P*. *multocida* isolates that were classified as treatment successes with no identification of subsequent clinical signs of BRD by the investigating veterinarian during the 42-day study.

## Results

1031 head (eleven truckloads) of English, Continental, and crossbred feeder heifers presumed to be at high risk of developing BRD (>40% morbidity) due to auction origin, comingling, and transportation, were enrolled on day-0 of the study. Mean day-0 weights with standard deviations and minimum/maximum weights are listed on Table 1.

**Table 1:** Weight parameters of day-0 and 1^st^ BRD-treatment cohorts.

Cohort	Mean Day-0 weight	Standard Deviation	Minimum to Maximum weight
Day-0 Tx success	227 kg. (499 lbs.)	25 kg. (56 lbs.)	144–292 kg. (317–642 lbs.)
Day-0 Tx failure	225 kg. (495 lbs.)	22 kg. (49 lbs.)	154–288 kg. (340–633 lbs.)
1^st^ BRD-Tx success	227 kg. (499 lbs.)	24 kg. (52 lbs.)	154–288 kg. (340–633 lbs.)
1^st^ BRD-Tx failure	224 kg. (493 lbs.)	21 kg. (46 lbs.)	169–262 kg. (372–577 lbs.)

Three heifers were confirmed to be persistently infected with BVDV (with agreement of both labs), were removed from the study within 5 days, and their data was excluded along with data from two animals with protocol deviations, leaving 1026 animals in the study. After the initial 7-day PMI following the day-0 administration of tulathromycin, 401 heifers (41%) were identified by the investigating veterinarian as having clinical signs of BRD, thus classified as treatment failures of day-0 tulathromycin administration however two protocol deviations were identified and data from those two animals were excluded from the study resulting in 399 heifers in the 1^st^ BRD-treatment cohort. Mean day of 1^st^ BRD-treatment was 13 days, with a range per arrival date of 8–16 days. TFR for day-0 samples ranged from 30–49% and 1^st^ BRD-treatment samples ranged from 51–60%.

Data presented in Table 2 indicates that TFR was similar regardless if the heifer was positive for *M*. *haemolytica* and *P*. *multocida* or negative. Greater TFR was seen in heifers with culture positive (49%) *M*. *haemolytica* at day-0 versus culture negative (40%) and heifers without *M*. *bovis* (41%) at day-0 compared to heifers with *M*. *bovis* (30%) at day-0.

**Table 2:** High-risk feeder heifer treatment failure rate by culture status and timing of sample.

Treatment Failure Rate by Pathogen and Sample Period					
		Culture Positive		Culture Negative	
Pathogen	Sample Period	#Failures/ Total #	%Treatment Failure	#Failures/Total #	%Treatment Failure
*M*. *haemolytica*	Day-0	55/112	49	366/914	40
	1^St-^BRD Treatment	69/136	51	148/263	56
*P*. *multocida*	Day-0	46/108	43	375/918	41
	1^St-^BRD Treatment	17/31	55	200/368	54
*M*. *bovis*	Day-0	3/10	30	418/1016	41
	1^St-^BRD Treatment	83/146	57	134/253	53
*H*. *somni*	Day-0	3/7	43	418/1019	41
	1^St-^BRD Treatment	15/25	60	202/374	54

**Fig 2:**
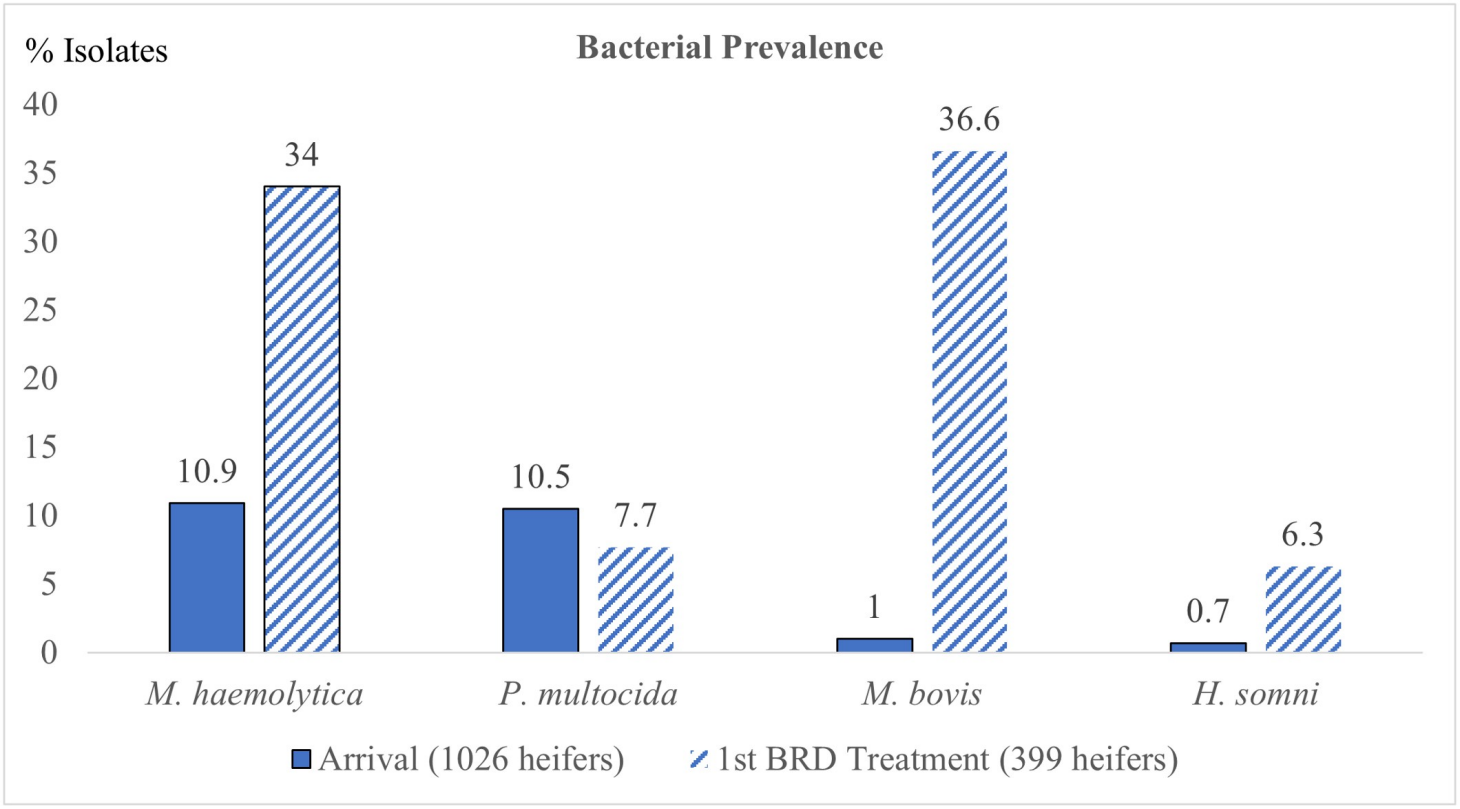
Prevalence of BRD pathogens from DNS in high-risk feeder heifers at day-0 and 1^st^ BRD-treatment. The y-axis is percent of isolates cultured and the numbers above the columns represent the number of animals with positive isolates.

No animals were treated on an emergency basis or euthanized because of debilitating clinical signs of BRD. Isolation rates for isolates of *M*. *haemolytica*, *P*. *multocida*, *H*. *somni*, and *M*. *bovis* collected from DNS on day-0 and 1^st^ BRD-treatment are summarized in Fig 2.

**Fig 3:**
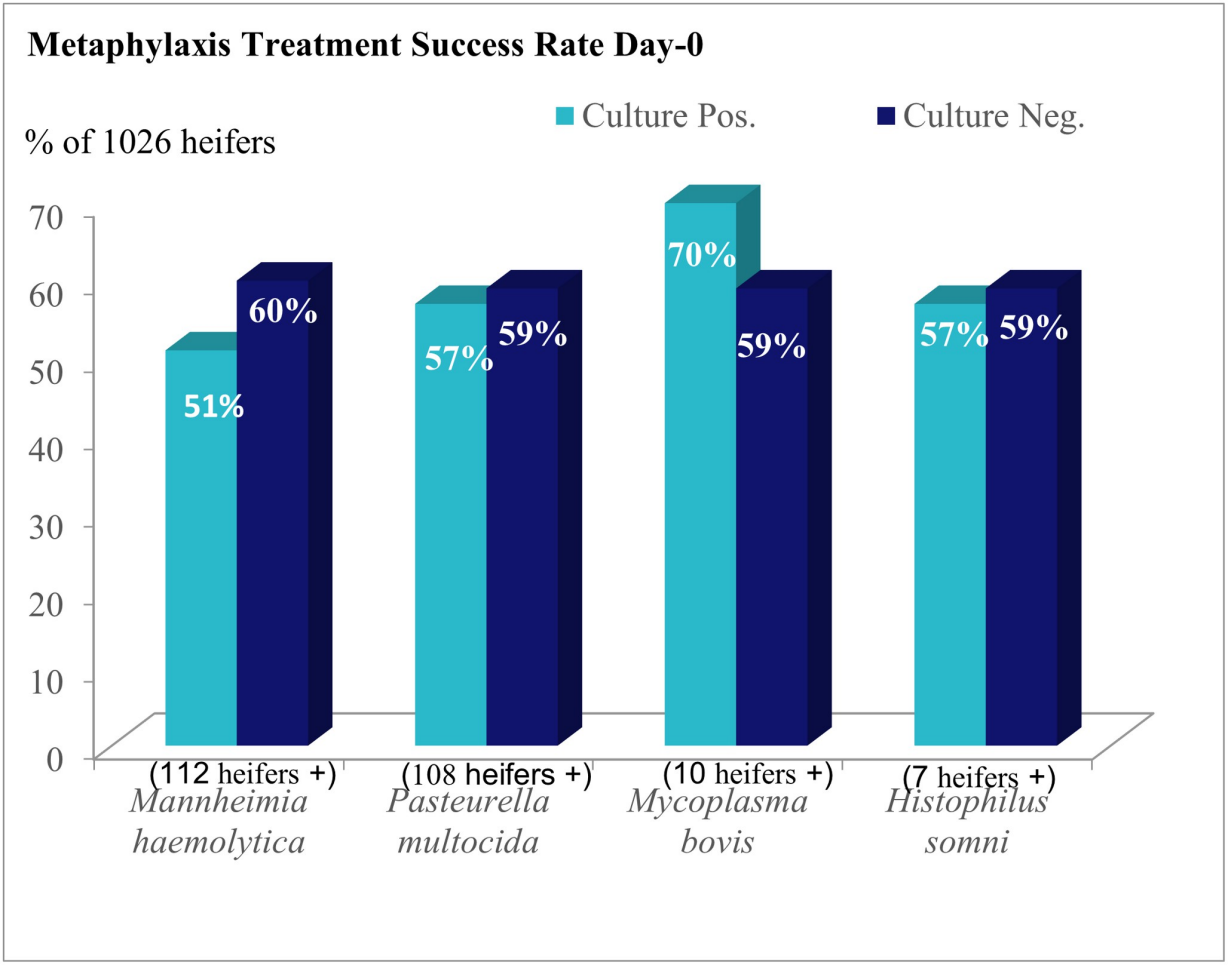
Metaphylaxis treatment success rate by culture of bacterial pathogen via nasopharyngeal swab at day-0.

Treatment success rates are illustrated in Fig 3. Over a hundred heifers were culture positive for *M*. *haemolytica* and *P*. *multocida* respectively however ten or less heifers were positive for *M*. *bovis* and *H*. *somni* on day-0. *M*. *haemolytica*, *H*. *somni*, and *M*. *bovis* had increased prevalence at 1^st^ BRD-treatment sampling but prevalence of *P*. *multocida* was similar at both sample periods.

**Fig 4:**
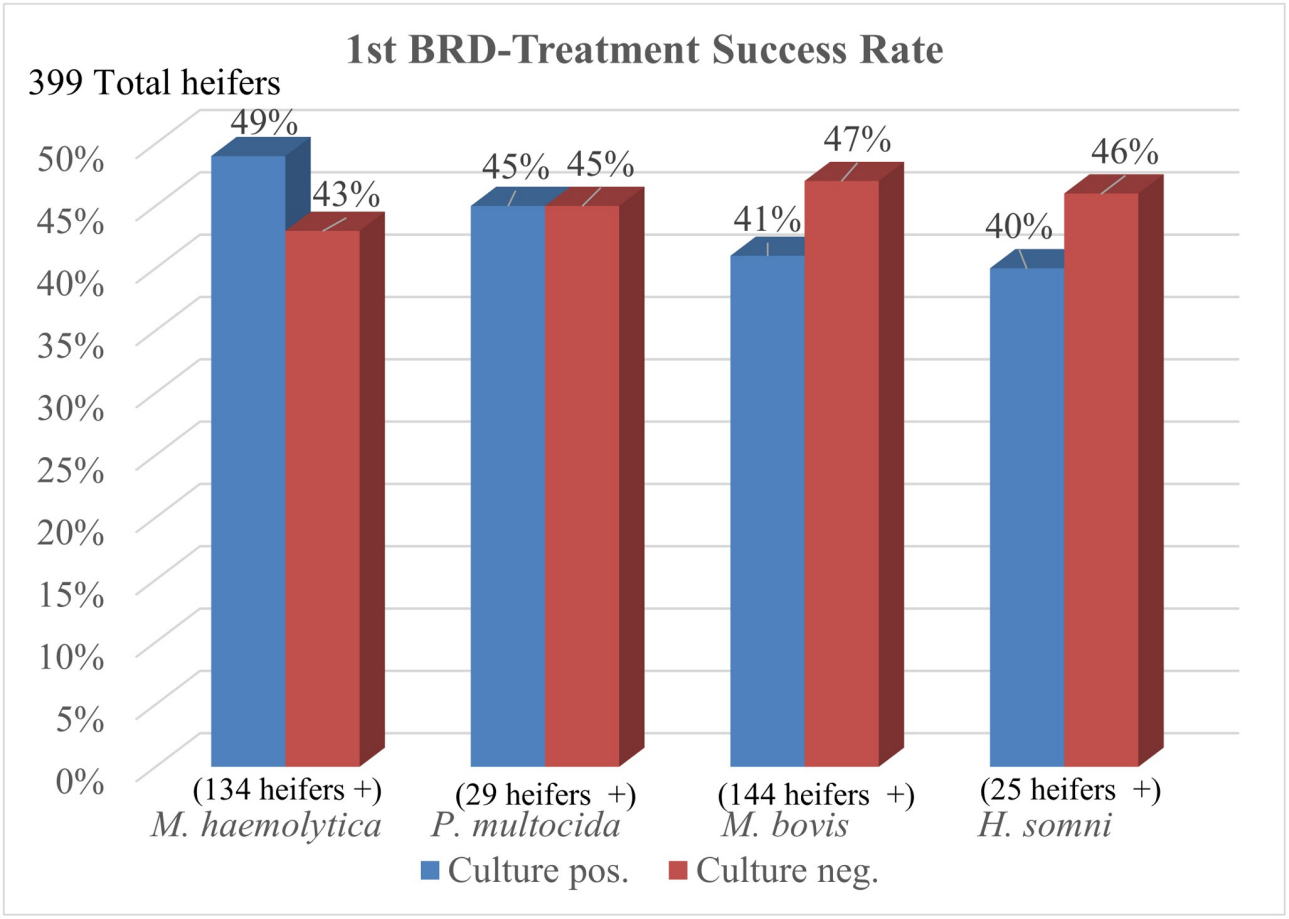
1^st^ BRD-treatment success rate by culture of bacterial pathogen via nasopharyngeal swab.

Fig 4 illustrates the treatment success rates for different BRD pathogens isolated from metaphylaxis treatment failures at the time of 1^st^ BRD-treatment. With a smaller number of heifers sampled, there were over one hundred positives for *M*. *haemolytica* and *M*. *bovis* respectively and at least 25 positive heifers for *P*. *multocida* and *H*. *somni*.

**Fig 5:**
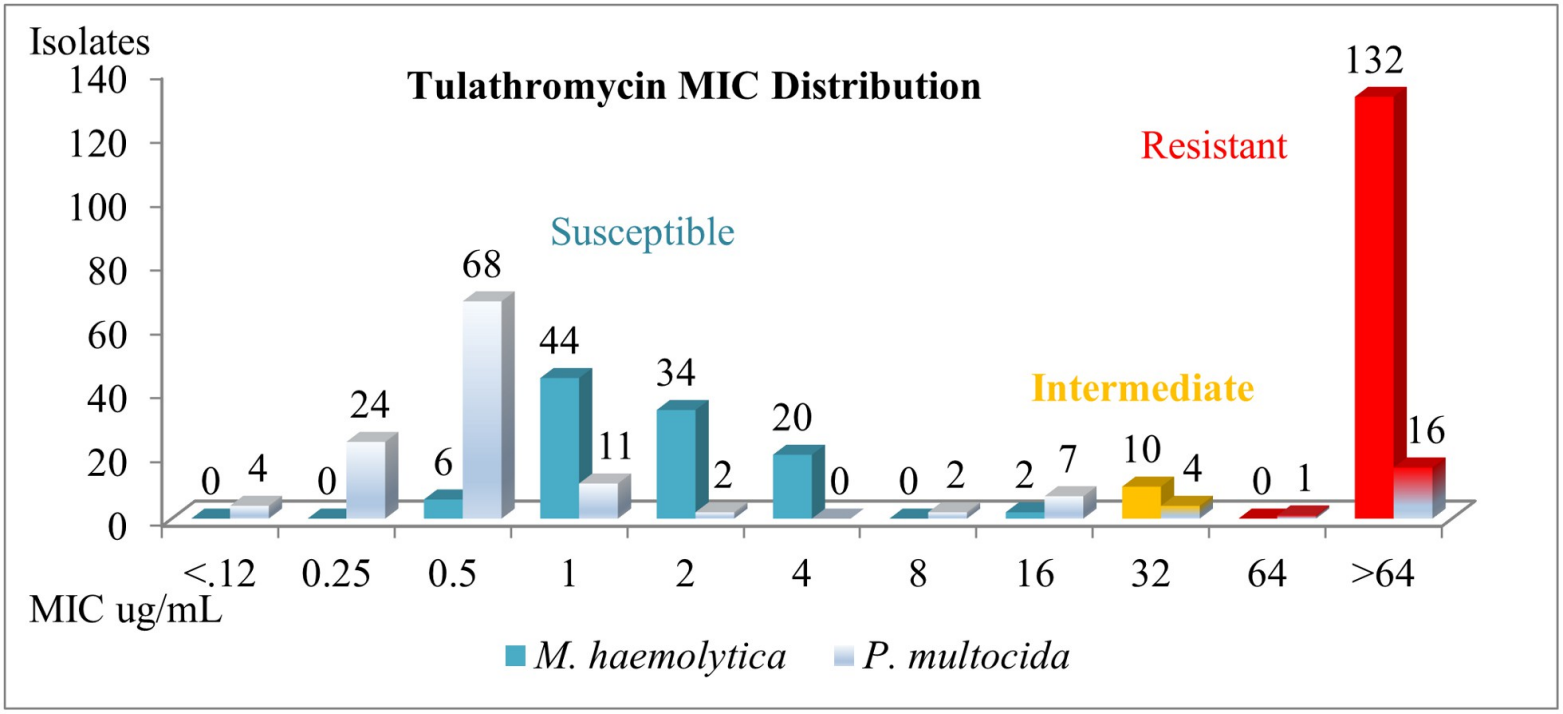
Tulathromycin mic frequency distribution of *M*. *haemolytica* and *P*. *multocida* isolates from all nasopharyngeal samples of high-risk feeder heifers.

Frequency distributions of MICs for all *M*. *haemolytica* and *P*. *multocida* isolates collected via DNS are summarized in Fig 5. Enough susceptible and resistant isolates of *M*. *haemolytica* and *P*. *multocida* were collected to provide meaningful analysis of the data. Day-0 and 1^st^ BRD-treatment isolates were combined in this histogram to save space but are analyzed separately for statistical parameters.

**Fig 6:**
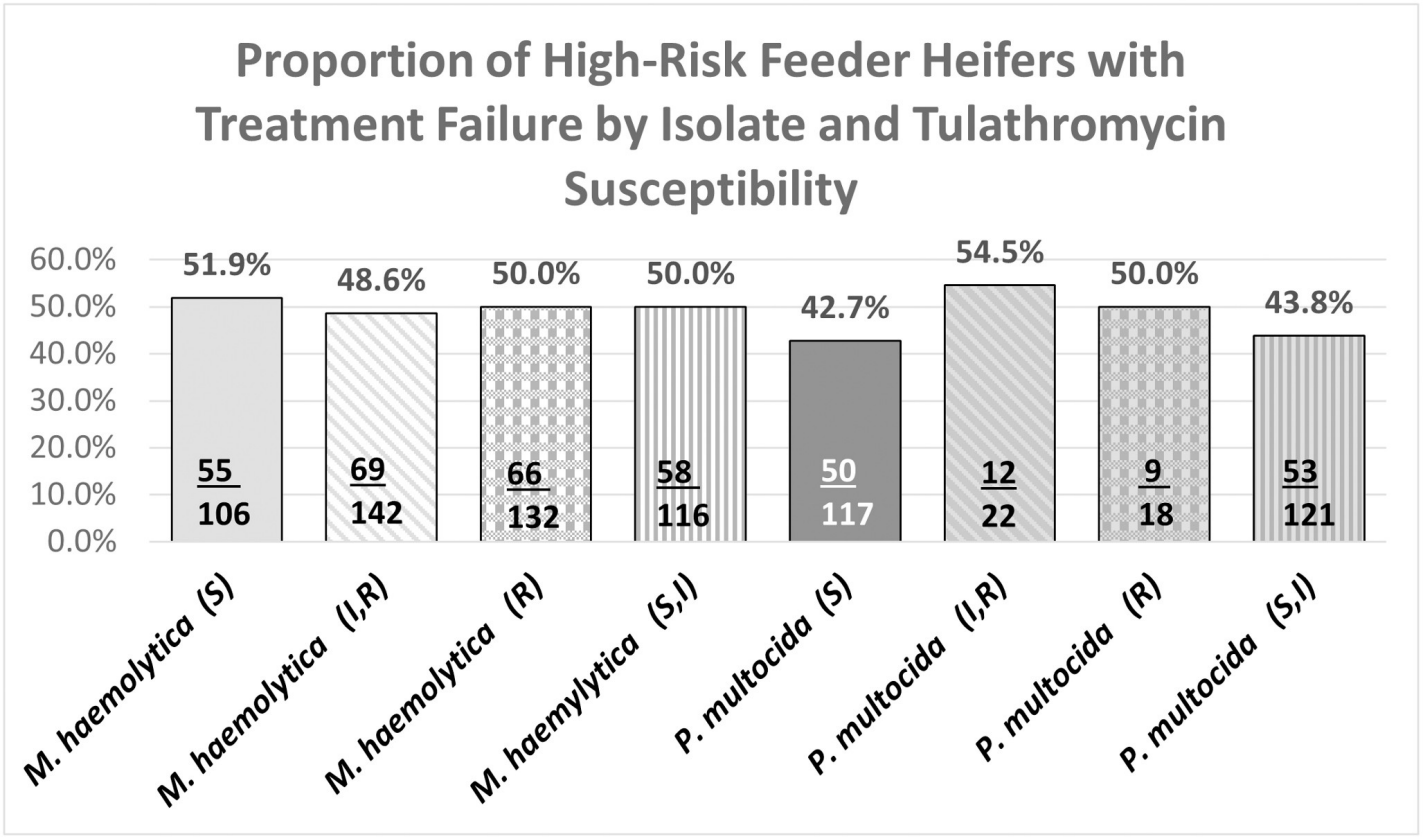
High-risk feeder heifer BRD treatment failure by bacterial isolate and tulathrymycin susceptibility.

Fig 6 illustrates no clinical difference in the TFR of *M*. *haemolytica* isolates classified as resistant or susceptible and only slightly lower TFR of susceptible P. *multocida isolates* compared to resistant isolates.

The TFR, (Table 3) for heifers with tulathromycin resistant *M*. *haemolytica* at 1^st^ BRD-treatment were lower (50%) than heifers with non-resistant *M*. *haemolytica* (66.7%) and heifers with *M*. *haemolytica* isolated at day-0 were very similar regardless of tulathromycin susceptibility. Heifers with tulathromycin susceptible *P*. *multocida* (41.7%) isolated at 1^st^ BRD-treatment had lower TFR than heifers with tulathromycin non-susceptible *P*. *multocida* (60%) however heifers with tulathromycin susceptible *M*. *haemolytica* at both day-0 (50.5%) and 1^st^ BRD-treatment (100%) had higher TFR than heifers with tulathromycin non-susceptible *M*. *haemolytica* at both day-0 (33.3%) and 1^st^ BRD-treatment (49.6%). There were insufficient numbers of heifers (NA) with tulathromycin resistant or non-susceptible *P*. *multocida* at day-0 for meaningful analysis.

**Table 3:** High-risk feeder heifer treatment failure rate by bacterial species and clsi tulathrymycin susceptibility classification.

Treatment Failure Rate (TFR)					
		Non-Resistant	Resistant	Non-Susceptible	Susceptible
Day-0	*M*. *haemolytica*	49.1%	50%	33.3%	50.5%
Day-0	*P*. *multocida*	42.9%	NA	NA	42.9%
1^st^ BRD- Treatment	*M*. *haemolytica*	66.7%	50%	49.6%	100%
1^st^ BRD- Treatment	*P*. *multocida*	50%	56.3%	60%	41.7%

CLSI MICs: *M*. *haemolytica* and *P*. *multocida*: susceptible, non-susceptible (>16 ug/ml), resistant (≥ 64 ug/ml), non-resistant (<64 ug/ml)

Table 4 lists parameters for bacterial isolates of DNS collected at day-0 or 1^st^ BRD-treatment from these heifers. Low sensitivity (ability of test to identify animals with treatment failure) was measured on both sample periods. High specificity (ability of test to identify animals with treatment success) was measured at both sample periods for most BRD isolates however, the PPV (proportion of animals with positive test and treatment failure) for both sample periods and NPV (proportion of animals with negative tests and treatment success) for both sample periods, indicate poor validity of isolation of *M*. *haemolytica*, *P*. *multocida*, *M*. *bovis or H*. *somni* from DNS at day-0 or 1^st^ BRD-treatment, for predicting BRD treatment outcomes.

**Table 4:** Se, Sp, PPV and NPV of *M*. *haemolytica*, *H*. *somni*, *M*. *bovis* and *P*. *multocida* isolates at day-0 and 1^st^ BRD-treatment.

Sample Time	Isolate	Se	Sp	PPV	NPV
**Day-0**	*M*. *haemolytica*	.11	.91	.49	.60
	*P*. *multocida*	.13	.90	.43	.59
	*H*. *somni*	.01	.99	.43	.59
	*M*. *bovis*	.01	.99	.30	.59
**1**^**st**^ **BRD-treatment**	*M*. *haemolytica*	.32	.63	.51	.43
	*P*. *multocida*	.08	.92	.55	.45
	*H*. *somni*	.07	.96	.60	.46
	*M*. *bovis*	.18	.66	.58	.47

Table 5 lists parameters of susceptible/non-susceptible or resistant/non-resistant isolates of *P*. *multocida* or *M*. *haemolytica* collected at day-0 or at 1^st^ BRD-treatment. NPV of cattle with *M*. *haemolytica* isolates not susceptible to tulathromycin on day-0 and PPV of cattle with *M*. *haemolytica* isolates susceptible to tulathromycin at 1^st^ BRD-treatment were 100% however, there was a small sample size in each cohort. NPV of heifers with *P*. *multocida* isolates not resistant to tulathromycin was 57% and predictive values for all other parameters were ≤51%. Comparing susceptible and non-susceptible isolates, high Se was measured for heifers with *M*. *haemolytica* on day-0 and when comparing resistant and non-resistant isolates, heifers with *M*. *haemolytica* and *P*. *multocida* isolates collected on day-0 had high Sp when comparing heifers with susceptible and non-susceptible *M*. *haemolytica* isolates and when comparing resistant and non-resistant *M*. *haemolytica* and *P*. *multocida* isolates. Parameters with NA were not analyzed due to zero heifers in at least one portion of the contingency table.

**Table 5:** Se, Sp, PPV, and NPV of tulathrymycin mic classification for *M*. *haemolytica* and *P*. *multocida* isolates collected from DNS samples from high-risk feeder heifers.

**Susceptible vs Non-Susceptible Isolates**					
**Sample Period**	**Isolate**	**PPV (S)**	**NPV (I&R)**	**Sensitivity (Failures)**	**Specificity (Successes)**
**Day-0**	*M*. *haemolytica*	.50	1.00	.98	.04
	*P*. *multocida*	.43	.42	1.00	.29
**1**^**st**^ **BRD-Treatment**	*M*. *haemolytica*	1.00	.50	.04	1.00
	*P*. *multocida*	.42	.40	.29	.53
**Resistant vs Non-Resistant Isolates**					
	**Isolate**	**PPV (R)**	**NPV (S&I)**	**Sensitivity (Successes)**	**Specificity (Failures)**
**Day-0**	*M*. *haemolytica*	NA	.51	NA	.98
	*P*. *multocida*	NA	.57	NA	.97
**1**^**st**^ **BRD-Treatment**	*M*. *haemolytica*	.50	.33	.94	.03
	*P*. *multocida*	.56	.50	.53	.53

Frequency distribution of multiplex PCR results from nasal swabs collected at 1^st^ BRD- treatment is summarized in Table 6. Prevalence of positive PCR results for these four common respiratory viruses was over 50% for BRSV, over one third for bPIV-3, and one fourth for BVDV in this study population of high-risk feeder cattle.

**Table 6:** Frequency of viral multiplex PCR from nasal secretions collected at 1^st^ BRD-treatment.

(399 heifers tested)	Positive PCR	%
**bPIV-3**	136	34
**BRSV**	216	54
**BVDV**	105	26
**BHV-1**	43	11

Table 7 shows that high-risk feeder heifers with positive viral PCR results from nasal swabs collected at 1^st^ BRD-treatment, had a higher percentage of treatment failure (ranging from 61% to 77%) compared to cattle that were negative to viral PCR at 1^st^ BRD-treatment (45 to 51%).

**Table 7:** Proportion of high-risk feeder heifers with treatment failure by viral multiplex PCR test.

Viral Pathogen (399 head)	Treatment Failure with Negative Viral PCR	Treatment Failure with Positive Viral PCR
**bPIV-3** (number of heifers)	49.4% (130)	62.5% (85)
**BRSV** (number of heifers)	45.4% (83)	61.1% (132)
**BVDV** (number of heifers)	48.8% (143)	67.9% (72)
**BHV-1** (number of heifers)	34.0% (181)	77.3% (34)

Data in Table 8 suggests acceptable to moderate PPV (proportion of test positive animals with treatment failure) for BHV-l (.77), BVDV (68), bPIV-3 (.62), and BRSV (.61) and satisfactory specificity (ability of test to correctly identify animals with treatment success) for BHV-1 (.99), BVDV (.78), and bPIV-3 (.71) although sensitivities (ability of test to correctly identify animals with treatment failure) for all viral pathogens were low. However, NPV for all BRD viral pathogens measured, were ≤ 51% which indicates poor validity of a negative viral PCR result to predict BRD treatment success.

**Table 8:** Se, Sp, PPV, NPV, and prevalence of viral PCR from nasal swab of high-risk heifers at 1^st^ BRD-treatment.

Microorganism	Sensitivity	Specificity	PPV	NPV	Prevalence
Bovine Herpes Virus-1	.15	.99	.77	.51	.11
Bovine Viral Diarrhea Virus	.33	.78	.68	.49	.26
Bovine Respiratory Syncytial Virus	.61	.45	.61	.45	.54
Parainfluenza Virus-3	.39	.71	.62	.49	.34

Data in Table 9 indicates, as the number of pathogens isolated per heifer increased, the proportion of cattle with treatment failure also increased. Greater frequency of one to three pathogens per animal was measured in this population with a median of two pathogens. Two thirds of the cattle showing signs of BRD at 1^st^ BRD-treatment, had >1 BRD pathogen isolated, illustrating prevalent polymicrobial involvement of BRD in these heifers.

**Table 9:** BRD treatment outcomes by number of BRD pathogens isolated at 1^st^ BRD-treatment.

# Pathogens	Total	%	# Failures	% Failures	# Success	% Success
0	45	11%	18	40%	27	60%
1	87	22%	42	48%	45	52%
2	125	32%	67	54%	58	46%
3	81	21%	50	62%	31	38%
4	42	11%	28	67%	14	33%
5	8	2%	8	100%	0	0%
≥6	11	3%	10	91%	1	9%
Total	399	100%	223	56%	176	44%

Table 10 summarizes the relative risk of treatment failure (RRTF) at 95% confidence intervals associated with a positive result for each of the diagnostic procedures analyzed in this study. A value <1.0 indicates less relative risk for treatment failure (greater probability of treatment success) and a value >1.0 indicates greater relative risk for treatment failure. 95% confidence intervals with lower and upper limits traversing 1.0 indicate uncertainty that the relative risk is increased or decreased. 95% confidence intervals mean that if the study is conducted multiple times with multiple sampling from the same population, 95% of the confidence intervals are expected to contain the true population mean [42]. Increased risk of treatment failure was associated with cattle having *M*. *haemolytica* isolates from DNS on day-0 and cattle with any positive viral PCR measured from nasal swabs, collected at 1^st^ BRD-treatment. No increased risks of treatment failure for any other parameters, were measured with 95% confidence in this study. Parameters with NA were not analyzed due to zero heifers in at least one portion of the contingency table.

**Table 10:** Relative risk of treatment failure for BRD diagnostic test methods from upper respiratory tract samples in high-risk feeder heifers.

Bacterial Isolates on Day-0	RRTF	95% Confidence Interval
*M*. *haemolytica*	1.23	1.00–1.51
*P*. *multocida*	1.03	.81–1.30
*H*. *somni*	.81	.26–2.52
*M*. *bovis*	.73	.28–1.89
**Resistant Isolates on Day-0**		
*M*. *haemolytica*	1.02	.25–4.13
*P*. *multocida*	NA	NA
**Susceptible Isolates on Day-0**		
*M*. *haemolytica*	1.51	.59–3.89
*P*. *multocida*	NA	NA
**Bacterial Isolates at 1**^**st**^ **BRD-treatment**		
*M*. *haemolytica*	.89	.73–1.09
*P*. *multocida*	1.01	.72–1.42
*H*. *somni*	1.01	.79–1.54
*M*. *bovis*	1.08	.90–1.30
**Resistant Isolates at 1**^**st**^ **BRD-treatment**		
*M*. *haemolytica*	.75	.42–1.35
*P*. *multocida*	1.12	.59–2.16
**Susceptible Isolates at 1**^**st**^ **BRD-treatment**		
*M*. *haemolytica*	NA	NA
*P*. *multocida*	.69	.32–1.48
**Viral PCR at 1**^**st**^ **BRD-treatment**		
BVDV	1.39	1.17–1.66
bPIV-3	1.26	1.06–1.51
BRSV	1.35	1.11–1.63
BVH-1	1.52	1.25–1.83
BVDV (-) & (Resistant) *M*. *haemolytica*	.62	.34–1.4
BVDV (+) & (Resistant) *M*. *haemolytica*	1.30	.32–5.38
bPIV-3 (-) & (Resistant) *M*. *haemolytica*	1.38	.27–6.94
bPIV-3 (+) & (Resistant) *M*. *haemolytica*	NA	NA
BRSV (-) & (Resistant) *M*. *haemolytica*	NA	NA
BRSV (+) & (Resistant) *M*. *haemolytica*	1.09	.40–2.97
BHV-1 (-) & (Resistant) *M*. *haemolytica*	.79	.38–1.66
BVDV (-) & (Resistant) *P*. *multocida*	.62	.34–1.4
BVDV (+) & (Resistant) *P*. *multocida*	1.30	.32–5.38
bPIV-3 (-) & (Resistant) *P*. *multocida*	1.38	.27–6.94
bPIV-3 (+) & (Resistant) *P*. *multocida*	NA	NA
BRSV (-) & (Resistant) *P*. *multocida*	NA	NA
BRSV (+) & (Resistant) *P*. *multocida*	1.09	.40–2.97
BHV-1 (-) & (Resistant) *P*. *multocida*	.79	.38–1.66
BHV-1 (+) & (Resistant) *P*. *multocida*	NA	NA
BVDV (-) & (Susceptible) *P*. *multocida*	.58	.24–1.39
BVDV (+) & (Susceptible) *P*. *multocida*	NA	NA
bPIV-3 (-) & (Susceptible) *P*. *multocida*	1.08	.40–2.92
bPIV-3 (+) & (Susceptible) *P*. *multocida*	.58	.20–1.63
BRSV (-) & (Susceptible) *P*. *multocida*	.59	.28–1.23
BRSV (+) & (Susceptible) *P*. *multocida*	NA	NA
BHV-1 (-) & (Susceptible) *P*. *multocida*	.73	.32–1.64
BHV-1 (+) & (Susceptible) *P*. *multocida*	NA	NA

## Discussion

Data from the current study indicates that results of DNS culture and tulathromycin susceptibility testing poorly predicts clinical outcome of tulathromycin administration at day-0 or 1^st^ BRD-treatment in heifers at high risk of BRD. Higher predictive values would make these diagnostic test methods more useful to practitioners however, the cost of testing in addition to inconsistent prevalence of any single BRD pathogen can nonetheless lead to less utility of these diagnostic methods. Isolation of *M*. *haemolytica* on day-0 was associated with an increased risk of BRD treatment failure; however, isolation of *M*. *haemolytica* at 1^st^ BRD-treatment or isolation of *P*. *multocida* either on day-0 or at 1^st^ BRD-treatment was not associated with a statistically predictable risk of BRD treatment outcome, which demonstrates the need to consider timing of a sample during the disease process when interpreting culture results. In spite of sample size of over one thousand animals at day-0 and almost four hundred animals sampled at 1^st^ BRD-treatment, tulathromycin susceptibility results failed to provide usefully predictive information with these sampling methods.

An increased RRTF associated with isolation of *M*. *haemolytica* on day-0 does not prove causation but rather association and since viral PCR on day-0 was not measured in this study, association of viral disease with isolation of *M*. *haemolytica* at day-0 is unknown, supporting further study of this association. Poor association of clinical outcomes to culture results in this study were similar to results reported by [43] who concluded that culture results for BRD pathogens have slightly better usefulness if combined with other diagnostic methods such as clinical signs however, in this study predictive values for bacterial culture were not better in the 1^st^ BRD-treatment cohort of cattle with clinical signs of BRD compared to day-0, healthy appearing cattle. An increased risk of treatment failure associated with isolation of *M*. *haemolytica* on day-0, has more utility with greater prevalence of *M*. *haemolytica* isolated due to more animals at risk but this creates an economic challenge because at least a proportion of the population of cattle has to be tested to estimate the prevalence of *M*. *haemolytica*. There would not be an economic advantage to identify cattle without *M*. *haemolytica* because decreased risk of treatment failure was not associated with negative *M*. *haemolytica* results.

Some investigators [11] assert that sampling the upper respiratory tract with DNS, leads to potential sampling errors and therefore only the lower respiratory tract should be sampled. As stated in the introduction, there has been conflicting information published on the association of culturing the upper respiratory tract and lower respiratory tract, implying additional research is needed to correlate alternative sampling methods with clinical outcomes of BRD [22–27]. Methods such as bronchoalveolar lavage, (BAL) and transtracheal aspirate, (TTA) to culture the lower respiratory tract in live cattle are available but not commonly used in the field due to lack of familiarization with the methods and potential contamination risks if implementation in feed yard environments.

Timsit [25] reported increased odds of BRD with culture of BRD bacterial pathogens from TTA samples, which could lead to greater adaptation of TTA sampling methods in the field. However, confidence in BAL or other sampling methods should be reserved until validation with evidence of adequate predictive value relative to clinical outcome. Unless the impacts of viral infections on the mucocilliary apparatus and other impacts on innate immune functions as well as influences of additional bacterial infections, e.g., *Mycoplasma bovis* or microbiome interactions and environments are accounted for, association of an in vitro test with clinical outcome of BRD may remain unreliable due to the polymicrobial complexity of BRD. Use of DNS can be questioned due to inconclusive evidence of association between upper respiratory tract and lower respiratory tract samples [22–27]. However, because DNS are more commonly performed in the field than other sampling methods such as TTA and BAL, there is greater external validity for DNS and this study presents valuable information to practitioners to assist in interpretation of DNS results.

Data in this study indicated, culture & tulathromycin susceptibility from DNS samples, were not highly reliable even when applying to the individual animal tested so applying individual animal results to a larger population of cattle for determining BRD treatment protocols, is even more problematic. Complexity of BRD, with inconsistent pathogen involvement, unpredictable pathogen virulence, variability in management and environmental risk factors, and irregular immune capabilities of cattle, leads to highly variable pathogen effects from case to case [2–5]. This study involved a sample size that is much larger than economically feasible to test, yet the unpredictable RRTF of resistant and susceptible isolates, did not yield reliable information to guide a decision to continue or not continue tulathromycin for BRD treatment or control based on culture and tulathromycin susceptibility results of *M*. *haemolytica* and *P*. *multocida* at day-0 or 1^st^ BRD-treatment.

One challenge with BRD is the lack of a single “gold standard test” for achieving a definitive diagnosis. The use of clinical signs such as lethargy, nasal discharge, anorexia, and dyspnea can be subjective and lessen the internal validity of the study. To address this, the investigating veterinarian who has extensive research and industry experience, assessed and scored all the cattle at approximately the same time each day, using the described CAS criteria. Presence (CAS1+ fever or CAS≥2) or absence (CAS = 0) of BRD clinical signs was used as the reference standard for statistical calculations. Using BRD clinical signs for the reference standard increases the external validity of the study because this method is the most common industry standard for diagnosing BRD compared to somewhat less subjective diagnostic tests such as thoracic ultrasound, auscultation, or blood tests.

Contrary to prevailing theory, cattle in this study with *M*. *haemolytica* or *P*. *multocida* isolates susceptible to tulathromycin were not associated with a higher likelihood of treatment success, and cattle with *M*. *haemolytica* or *P*. *multocida* isolates resistant to tulathromycin were not associated with a higher likelihood of treatment failure. In fact, cattle with tulathromycin susceptible *M*. *haemolytica* at day-0 and 1^st^ BRD-treatment had greater TFR than cattle with tulathromycin resistant *M*. *haemolytica* and heifers with tulathromycin resistant *M*. *haemolytica* had equal TFR at day-0 sampling and lower TFR at 1^st^ BRD-treatment sampling than heifers with tulathromycin non-resistant *M*. *haemolytica*. Failure of culture and sensitivity results to predict BRD clinical outcome in this study resembles results from Klement [34] who reported that antimicrobial susceptibility results of common bovine mastitis pathogens poorly predicted clinical outcome. Evidence in this study, refutes the paradigm that applying tulathromycin susceptibility results from DNS, to select BRD treatment or control, will result in superior BRD treatment outcomes [34]. McClary [44] reported similar results in a retrospective study comparing tilmicosin susceptibility results and clinical outcome from 1297 cattle with bacterial isolates of BRD pathogens in 16 controlled clinical trials where they determined the proportion of treatment failures attributed to *M*. *haemolytica* isolates categorized as resistant (MIC of tilmicosin, ≥ 32 μg/mL) or not susceptible (MIC of tilmicosin, ≥ 16 μg/mL), was 0.2% and 0.5%, respectively. Results of McClary [44] and the current study, indicate that contrary to common belief, most BRD treatment failures following tilmicosin or tulathromycin administration are likely not due to antimicrobial resistance. Factors that susceptibility testing fails to account for such as viral and bacterial co-infections; timing of treatment relative to disease occurrence; stress and management of cattle; and immune function of cattle with BRD are more common causes of treatment failures which would account for poor association of susceptibility results with clinical outcome.

Turnaround time for these diagnostic test methods, also creates challenges for application of results because delays and additional stress of handling cattle is required. Cattle with increased probability of having *M*. *haemolytica* due to management or environmental risks, (weaning, comingling, transportation, season, weather, etc.), are more likely to receive metaphylactic antimicrobial treatment due to increased risk of other bacterial pathogens which leads to less benefit of culturing high-risk cattle at arrival. Inadequate association of tulathromycin susceptibility testing from DNS collected at day-0 and 1^st^ treatment-BRD with clinical outcomes, provides evidence that relying on susceptibility testing to evaluate or predict tulathromycin efficacy in this population, is unreliable. This also emphasizes an important challenge of culture and antimicrobial susceptibility which is the need for more research of host models as a primary approach to understanding host–pathogen interactions in antibiotic resistance [33].

There are no CLSI standards pertaining to sample method, location, treatment versus metaphylaxis application, or timing of the disease process for BRD isolates so these factors need to be considered when interpreting susceptibility results as they may confound results. Results of the current study reinforce the idea that timing of the sample is important for interpreting the results of bacterial culture and susceptibility testing. *M*. *haemolytica* collected at day-0 was associated with lower treatment success and isolation of *M*. *haemolytica* at 1^st^ BRD-treatment was associated with greater treatment success, however, the opposite was seen from cattle with isolates of *M*. *bovis* at day-0 compared to 1^st^ BRD-treatment. Other investigators have shown differences in prevalence when sampling cattle at arrival and at different times of the feeding period [16, 43] as well as when sampling cattle showing signs of BRD versus cattle not showing signs of BRD [16, 26, 28] or cattle that have died due to BRD. Effect of variations in sample methodology and timing is uncertain and warrants further investigation.

Ersoy and colleagues [35] question the validity of using MHB, a rich laboratory medium that fails to mimic most aspects of host environments rather than media that account for pathogen conditions in the host [3]. They report that standard antimicrobial susceptibility testing failed to detect antibiotics that are in fact effective in vivo; and frequently identified antibiotics that were instead ineffective as further confirmed in mouse models of infection and sepsis. Conversely, antimicrobial susceptibility testing performed in media mimicking host environments, succeeded in identifying specific antibiotics that were effective in bacterial clearance and host survival, even though these same antibiotics failed in results using standard test media. Poor association of antimicrobial susceptibility testing with clinical signs found in this study and others [44], justifies further research and careful interpretation of antimicrobial susceptibility results.

One explanation for the lack of association of tulathromycin resistant isolates of *M*. *haemolytica* and *P*. *multocida* collected via DNS from cattle identified with clinical signs of BRD, with greater treatment failure rates, could be potential immunomodulatory effects of tulathromycin that have been reported by several investigators [45–48]. Failure of tulathromycin susceptibility results from DNS cultures of *M*. *haemolytica* and *P*. *multocida* to predict BRD clinical outcomes could also be due to poor association of bacterial isolates present in the upper respiratory tract and bacterial organisms causing disease in the lower respiratory tract [24–27]. This hypothesis needs further investigation as other researchers like DeRosa [22] reported good association of DNS and TTA in a small cohort of calves. Godinho [23] reported good association of DNS with lung lavage which better correlated with DNS isolates than lung swab or lung tissue homogenate. Harhay [28] reported genome sequences from isolates of DNS and BAL samples associated with antibiograms and all 16 genomes exhibited N6-adenine methylation at the GATC motif, while no other base modifications were detected however, antibiograms demonstrated variation in antimicrobial resistances between the sequenced isolates. Using genomic sequencing, Zeineldin [26] concluded, significant differences in the microbial community structure of the DNS and BAL samples indicated that a clear distinction exists between the microbiota at these sites. However, strong associations between the presence of several specific taxa in DNS samples and those from BAL supports the notion of the existence of a mutualistic inter-relationship between these biogeographically disparate microbial communities [26]. Doyle [27] reported good agreement of nasal swabs, DNS and BAL with TTA in dairy calves, diagnosed with clinical signs of BRD and lung lesions verified with thoracic ultrasound. Inconsistent research methods and results on association of bacterial isolates from the upper respiratory tract and lower respiratory tract warrant further research in this area especially with respect to clinical outcome. In addition, future studies, in large cohorts of animals, are needed to determine the role and clinical importance of the relationships of respiratory tract microbial communities with health, productivity, and probability of developing respiratory disease in growing cattle.

Since tulathromycin was administered at day-0, potential effect(s) of prior antimicrobial administration may have impacted 1^st^ BRD-treatment isolates. Timing of the 1^st^ BRD-treatment samples in this study were at least eight days after administration of day-0 tulathromycin with a mean of 13 days; however, pharmacokinetic properties of tulathromycin indicate therapeutic levels for up to 15 days [48–50]. Some of the 1^st^ BRD-treatment cattle may have had remaining levels of tulathromycin from the day-0 administration at the 1^st^ BRD-treatment sample time. This could confound bacterial culture or susceptibility results because tulathromycin concentrations might have been higher than dosages calculated for CLSI breakpoints [30] due to overlapping administration of tulathromycin. With greater concentrations of tulathromycin at 1^st^ BRD-treatment, greater treatment success would have been expected. If overlapping tulathromycin administrations resulted in better TSR for cattle with tulathromycin susceptible isolates, then the true association of tulathromycin susceptibility and treatment success would have been expected to be lower than measured in this study. RRTF for susceptible isolates should be <1.0 indicating a decreased risk of treatment failure (increased risk of treatment success) and greater levels of tulathromycin should have led to lower risk of treatment failure in tulathromycin susceptible isolates but even with potential greater levels of tulathromycin, the 95% confidence intervals were not <1. Greater tulathromycin concentrations at 1^st^ BRD-treatment due to potential overlap of day-0 and 1^st^ BRD-treatment, might have led to less treatment failures for resistant isolates which is a limiting factor of this study protocol. However, 7-day PMI/PTI periods were observed because they are more commonly practiced in the industry and therefore lend greater external validity to the study. Tulathromycin was used for both metaphylaxis and 1^st^ BRD-treatment to simplify the study protocol to a single antimicrobial agent and eliminate possible antimicrobial interactions. Using the same antimicrobial agent for metaphylaxis and 1^st^ BRD-treatment gives this study less external validity, since it is not a common practice. However, evidence to support contraindications is inconclusive or absent. CLSI breakpoint predictions [30] do not take into consideration previous antimicrobial therapy regardless if it is the same antimicrobial or a different antimicrobial so effects of previous antimicrobial therapy on subsequent MIC’s is uncertain.

Another limitation of this study was the low prevalence of resistant isolates to tulathromycin on day-0. The population of high-risk feeder heifers was selected to mitigate bias from variable proportions of steers and bulls and the risk of BRD associated with castration. This population was also selected because tulathromycin resistance was historically found in samples from previous cattle from these sources that also had prior history of poor TSR to tulathromycin, (assumed to be from increased prevalence of tulathromycin resistance). Regardless of low prevalence of tulathromycin resistance on day-0 in this population of high-risk feeder heifers, interpretation of poor association of tulathromycin resistance with 1^st^ BRD-treatment failure, is still meaningful because enough tulathromycin resistant isolates were identified to perform satisfactory statistical analysis in the 1^st^ BRD-treatment cohort. External validity of this study applies to association of these diagnostic test methods with clinical outcome following BRD tulathromycin metaphylaxis/treatment of high-risk feeder heifers purchased in auction facilities in the southeastern U.S. or south-central Texas and transported over eight hours to feed yard facilities in the central U.S.

Confounding of tulathromycin susceptibility testing from 1^st^ BRD-treatment samples may have resulted from day-0 tulathromycin therapy killing susceptible bacteria and leaving resistant bacteria to be cultured from the upper respiratory tract with absence of clinical signs of BRD or clinical signs of BRD due to other pathogens. Similar confounding can be expected, regardless of sample location or method or when sampling live or dead animals after antimicrobial treatment which makes interpretation of post-treatment antimicrobial susceptibility results problematic [51–53]. Culturing lung tissue collected at necropsy is a common submission to diagnostic labs because results can be better correlated with lesions at the site of infection although, treatment history with antimicrobials has still been shown to affect results [53]. A common (mis)-interpretation of antimicrobial susceptibility results is to interpret a post-treatment sample with an isolate that is resistant to the antimicrobial used for treatment and correlate treatment failure to antimicrobial resistance although, data from this study and McClary [44] would refute that assumption for tulathromycin and tilmicosin administration. If the antimicrobial used for treatment was totally effective, elimination of all susceptible bacterial pathogens should occur, leaving either no bacterial pathogens or only bacteria resistant to the antimicrobial used. Consequently, post-treatment isolation of susceptible isolates, would suggest less efficacy of the antimicrobial used for treatment and merit selection of a different antimicrobial for subsequent antimicrobial treatment and presence of resistant isolates would be associated with increased effectiveness of the antimicrobial therapy used.

Another limitation of bacterial culture and antimicrobial susceptibility testing is there is poor quantification of bacteria which may have led to poor association of BRD pathogens to clinical disease in this and other research [54]. Historically, characterization of the cattle microbiota has relied heavily on culture-dependent techniques which have mainly focused on the identification of major pathogens that can be easily cultured and susceptibility testing is typically only performed and reported on one representative colony from the sample submitted, which is a limitation of this study as well as antimicrobial susceptibility results from diagnostic laboratories. This may lead to misrepresentation of the bacterial pathogen because of strain variations in virulence, MICs, transmissibility, and other phenotypic or genotypic factors.

Similar to testing only one colony from a sample, culturing a small sample size of a subset of animals, (1–3 fatal BRD treatment failures, or cattle with BRD) and applying those result to a larger population (all cattle with BRD), has a high potential for bias and undesirable results due to the complex and variable etiology of BRD. Clinical outcome of treatment protocols for populations of cattle may also be confounded by prevalence of natural antimicrobial resistance in bacteria microbiomes or phenotypic expression of resistant genes following antimicrobial therapy [54].

In addition to sample populations, timing, location, methodology, and treatment history, sample shipping time and handling needs to be considered when interpreting or deciding to preform antimicrobial susceptibility. Samples in this study were handled in a typical manner and shipped to the laboratory overnight, although samples taken on the weekends had to be refrigerated and held for shipment for 1–2 days. This could have affected the number of isolates cultured however, over 70% of the samples were shipped the day they were collected and several cultures held over the weekend resulted in positive isolates so any impact of sample shipping/handling on the outcome of this study, should have been minimal but remains a constraining factor for the internal validity of the study.

Multiplex PCR data collected in this study, show increased risk of treatment failure with positive viral PCR swabs collected at 1^st^ BRD-treatment. Conversely, data from these high-risk feeder heifers, indicate negative viral PCR results collected at 1^st^ BRD-treatment, were not associated with lower risk of treatment failure. Therefore, utility of PCR testing at 1^st^ BRD-treatment would be dependent on a higher prevalence of viral pathogens however, BRD presents a challenge because viral pathogen prevalence is unpredictable without testing because sampling cohorts of cattle with low prevalence of viral pathogens would result in added expense with low return on investment.

A confounding effect of BVDV and BHV-1 viral PCR results in this study, was vaccination with a pentavalent MLV respiratory vaccine on day-0 and days 10–14. Revaccination was not in the initial protocol but due to high morbidity in the initial cohort, the investigating veterinarian and owner of the cattle requested an amendment to the protocol, allowing revaccination with the MLV pentavalent respiratory vaccine. Inability to discern vaccine virus from wild virus limits the diagnostic usefulness of multiplex PCR from nasal swabs collected at 1^st^ BRD-treatment, due to confounding from MLV vaccination [55]. Interpretation of viral BHV-1 and BVDV PCR results in this study and in general, is problematic due to uncertainty of whether viral nucleic acid detected is from live or inactive viruses or if the nucleic acid came from vaccine or wild viruses as well as whether it represents infection or not. However, Waltz [55] found that BRSV virus does not disseminate and replicate to the nasopharyngeal mucosa after vaccination with the SQ pentavalent MLV respiratory vaccine used in this study. Waltz [55] found no positive BRSV PCR results from DNS when sampled at 3,5,7,14,21,28,35, or 42 days after vaccination with the administration of the pentavalent BRD viral vaccine used in the current study and his study. Therefore, there is evidence that the specific pentavalent MLV respiratory vaccine used in this study would not present confounding for the BRSV positive samples. However, Waltz [55] did find 20% positive at 14 days and 10% positive at 21 days for BHV-1 and 30% positive at 7 days and 40% positive at 14 days for BVDV after vaccination with this MLV pentavalent respiratory vaccine, indicating systemic replication of BVDV and BHV-1 vaccine virus which would create confounding for BVDV and BHV-1 positive samples in the current study. Replication of bPIV-3 vaccine virus in naso-pharyngeal tissues after subcutaneous MLV vaccination is unknown. Since bPIV-3 has not been reported to replicate systemically, spread from a subcutaneous vaccination site to the naso-pharyngeal tissues would not be expected, indicating positive bPIV-3 PCR results at 1^st^ BRD-treatment in this study likely represent wild virus. The impact of removal of three BVDV persistently infected heifers at the beginning of the study is unknown however, an increased prevalence of BVDV at 1^st^ BRD-treatment might have been expected if the BVDV persistently infected heifers were allowed to remain with the cohorts throughout the 42-day study period. Obviously, some heifers in the study were exposed to the BVDV persistently infected heifers during transportation and early in the study period but the impact on BRD outcome and BVDV prevalence is uncertain due to confounding of modified live viral BVD vaccination.

RRTF was analyzed for each of the multiplex viral agents along with tulathromycin susceptibility of *M*. *haemolytica* and *P*. *multocida* isolates. No predictable risk of treatment failure was associated with any combination of BRD viral agent and isolates of *M*. *haemolytica* or *P*. *multocida* regardless of tulathromycin susceptibility although all of the tulathromycin susceptible *M*. *haemolytica* cohorts and various other cohorts had insufficient numbers of animals in at least one of the units of the contingency tables (NA) and several other viral/bacterial combinations analyzed had low numbers of animals that could affect the confidence intervals. Lack of predictable increased RRTF of any of the viral and *M*. *haemolytica* or *P*. *multocida* combinations, even with 54% prevalence of BRSV, illustrates the unpredictable polymicrobial nature of BRD and the challenge of using monomicrobial tests to evaluate BRD clinical outcome.

The meaningfulness of these viral PCR results should be interpreted considering both the RRTF and prevalence, i.e., RRTF was higher for BVD and BHV-1 but prevalence was higher for BRSV and bPIV-3 leading to similar overall risk of BRD treatment failure for all viral pathogens in this population. It is possible that 1^st^ BRD-treatment failures were more likely due to viral involvement rather than antimicrobial resistance in this study because of the association with increased risk of treatment failure for heifers with positive viral PCR but is not proof of causation. Further investigation is needed to more clearly define the association of vaccine and/or wild virus to better interpret results of BRD viral PCR testing.

Results of this study and McClary [44], suggest BRD treatment failures are more likely due to factors other than antimicrobial resistance. Poor association with clinical outcomes, indicates relying on bacterial culture and tulathromycin susceptibility testing from DNS as the primary driver in determining BRD tulathromycin metaphylaxis/treatment protocols would not be prudent. Considering the time, expertise, and expense involved in doing these diagnostic procedures and the failure of these diagnostic methods to predict the clinical outcome of BRD, (validated with lack of predictable risk of treatment failure, poor sensitivity, specificity, and predictive values), the dependability of DNS bacterial culture and tulathromycin susceptibility in *P*. *multocida* and *M*. *haemolytica* isolates for evaluating the effectiveness of tulathromycin for the control or treatment of BRD in high-risk heifers, is unreliable. Data from this study and McClary [44] coincide that developing successful BRD treatment protocols is not as simple as applying tulathromycin or tilmicosin susceptibility results.

Bearing in mind the discussed limitations and biases of these BRD diagnostic test methods, applying principles of evidence-based medicine such as information from controlled clinical trials or meta-analyses would provide more credible information for developing BRD treatment protocols and combating antimicrobial resistance compared to relying solely on MIC’s specific to monomicrobial pathogens [29].

## Conclusions

While inferences to the predictive values of susceptibility testing for other antibiotics cannot be drawn from this study, the results indicate that relying solely on the use of tulathromycin susceptibility testing for isolates of *M*. *haemolytica* and *P*. *multocida* derived from DNS collected on day-0 or 1^st^ BRD-treatment from high-risk feeder heifers, poorly predicted clinical outcome for tulathromycin metaphylaxis/treatment.

Statistical increased risk of treatment failure, at 95% confident intervals, was associated with evidence of viral genetic material through PCR testing of nasal secretions collected at 1^st^ BRD-treatment and culture presence of *M*. *haemolytica* from DNS collected on day-0. However, confounding from MLV vaccination leaves questions to the impact of BHV-1 and BVDV in this study and warrants further investigation. Se, Sp, PPV, and NPV for bacterial culture and tulathromycin susceptibility were inconsistent in this study indicating less utility for bacterial culture and tulathromycin susceptibility testing from DNS in high-risk heifers. These results and others’ [31, 44] indicate that BRD treatment failures are not commonly associated with antimicrobial resistance.

Potential limitations of using the results of bacterial culture and susceptibility for selecting antimicrobial agents for BRD treatment or control are: 1) bacteria isolated in the upper respiratory tract via DNS sampling may not match pathogens causing infection in the lower respiratory tract, 2) susceptibility results are specific to one bacterial pathogen from a representative colony that may or may not represent the infectious pathogens, especially considering the complex etiology of BRD, 3) some antibiotics such as tulathromycin can have in vivo effects besides inhibiting bacterial growth or killing bacteria such as enhancing innate immune function, 4) the animal’s immune function may be sufficiently robust to clear infections without antimicrobial therapy or immunocompromised to the level that no antimicrobial agents are effective.

Considering limitations of bacterial culture and antimicrobial susceptibility testing, better development of BRD treatment or control protocols might come from using information from sources such as randomized controlled clinical trials or meta-analyses, with better control of bias and confounding. Results of the current study indicate using bacterial culture and tulathromycin susceptibility testing of DNS from high-risk feeder heifers, for predicting the efficacy of tulathromycin for metaphylaxis/treatment of BRD, is unreliable.

## Supporting information

S1 Appendix Clinical Appearance Score (CAS). (DOCX)

S2 Appendix Arrival products and procedures. (DOCX)

S3 Appendix Analysis equations. (DOCX)

S4 Appendix (DOCX)

S1 File (PDF)

## Abbreviations

BA: blood agar
BAL: bronchoalveolar lavage
BHV-1: bovine herpes virus type-1
bPIV-3: bovine parainfluenza virus type-3
BQA: Beef Quality Assurance
BRD: bovine respiratory disease
BRSV: bovine respiratory syncytial virus
BVDV: bovine viral diarrhea virus
CAS: clinical appearance score
CLSI: Clinical and Laboratory Standards Institute
DNS: deep nasopharyngeal swab
*H*. *somni*: *Histophilus somni*
HFA: Hayflick’s agar
*M*. *bovis*: *Mycoplasma bovis*
*M*. *haemolytica*: *Mannheimia haemolytica*
MHB: Mueller Hinton broth
MIC: minimum inhibitory concentration
Multiplex: PCR, BVDV, BRSV, BHV-1, bPIV-3
NA: not analyzed due to insufficient numbers
NPV: Negative Predictive Value
*P*. *multocida*: *Pasteurella multocida*
PCR: polymerase chain reaction
PD: pharmacodynamic
PMI: post metaphylaxis interval
PPV: positive predictive value
PTI: post treatment interval
RRTF: relative risk for treatment failure
Se: sensitivity
Sp: specificity
TFR: treatment failure rate
TSR: treatment success rate
TTA: transtracheal aspirate
