# Supplementary material for: Limitations of bacterial culture, viral PCR, and tulathromycin susceptibility from upper respiratory tract samples in predicting clinical outcome of tulathromycin control or treatment of bovine respiratory disease in high-risk feeder heifers
Source: PLoS One. 2022 Feb 10;17(2):e0247213. doi: 10.1371/journal.pone.0247213 (PMC8830659; doi:10.1371/journal.pone.0247213)
Supplement: S1 Appendix — (DOCX) [file pone.0247213.s001.docx]

**S1 Appendix.**

**CLINICAL APPEARANCE SCORE (CAS).**

| **CAS Score** | **Appearance Descriptors** |
| --- | --- |
| **0**  No BRD | No BRD clinical signs; calf is healthy bright, alert, and responsive when approached. |
| **1**  Mild BRD | Calf looks ill until approached by observer (calf brightens up, moves readily and appears normal); if observer loses sight of calf and upon re-exam of the pen, they CANNOT readily locate and ID the calf as ill.  Mild depression; slower in movement but no signs of weakness; small amount of serous nasal discharge; slight ocular discharge. |
| **2**  Moderate BRD | Calf is obviously ill with BRD; the calf shows no change in appearance when approached by observer (does not brighten up and moves slowly or reluctantly). If observer loses sight of calf, upon re-exam of pen, they CAN easily locate and ID the calf as ill.  Moderate depression; signs of weakness or “knuckling” and calf may be reluctant to stand or move about pen; some shallowness apparent in left flank; considerable serous nasal discharge or moderate amount of mucopurulent nasal discharge; dyspnea or respiratory rate is increased; cough or coughing episodes are present. |
| **3**  Severe BRD | Calf is severely ill with BRD; the calf shows no change in appearance when approached by observer; abnormal respiration and/or depression present.  Severe depression; stumbling or moves with extreme prodding; obvious lack of fill in left flank signaling anorexia; head lowered or extended to facilitate breathing; may be open-mouthed breathing with considerable noise (expiratory grunts, moans); copious mucopurulent to purulent nasal discharge; cough or coughing episodes are present.  Administer emergency intervention therapy. |
| **4**  Moribund | Calf is moribund and near death – calf in general is not ambulatory, cannot rise from recumbency, and cannot acquire food or water. Very likely cannot be removed from pen for treatment without mechanical transport.  Euthanize humanely. |
