# Supplementary material for: Limitations of bacterial culture, viral PCR, and tulathromycin susceptibility from upper respiratory tract samples in predicting clinical outcome of tulathromycin control or treatment of bovine respiratory disease in high-risk feeder heifers
Source: PLoS One. 2022 Feb 10;17(2):e0247213. doi: 10.1371/journal.pone.0247213 (PMC8830659; doi:10.1371/journal.pone.0247213)
Supplement: S2 Appendix — (DOCX) [file pone.0247213.s002.docx]

**S2 Appendix.**

**ARRIVAL PRODUCTS AND PROCEDURES.**

Penta-valent BRD viral vaccine (Bovi-Shield GOLD^®^ 5) 2mL SQ neck

Hepta-valent Clostridial vaccine (UltraChoice^TM^ 7) 2mL SQ neck

Injectable Anthelmintic (Dectomax^®^ Injectable Solution) 200mcg/kg body weight SQ neck

Growth promoting implant (Synovex^®^ H) 1 dose SQ in center 1/3 of ear

Two ear notch samples BVDV Elisa testing

Two Identification Ear Tags (Allflex^®^) ear tag each ear

Tulathromycin injectable solution (Draxxin^®^) 2.5mg/kg body weight SQ
