# Supplementary material for: Limitations of bacterial culture, viral PCR, and tulathromycin susceptibility from upper respiratory tract samples in predicting clinical outcome of tulathromycin control or treatment of bovine respiratory disease in high-risk feeder heifers
Source: PLoS One. 2022 Feb 10;17(2):e0247213. doi: 10.1371/journal.pone.0247213 (PMC8830659; doi:10.1371/journal.pone.0247213)
Supplement: S3 Appendix — (DOCX) [file pone.0247213.s003.docx]

**S3 Appendix.**

**ANALYSIS EQUATIONS.**

**Contingency Table A** *True positive*  **B** *False Positive*

**C** *False Negative* **D** *True Negative*

**Bacterial Culture or Viral PCR**

**Sensitivity = A__**

**A + C**

= Number of animals with positive test classified as treatment failure

Number of animals classified as treatment failure

**Specificity = _ D___**

**D+B**

= Number of animals with negative test classified as treatment success

Number of animals classified as treatment success

**Positive Predictive Value =** __**A**__

**A + B**

= Number of animals with positive test classified as treatment failure

Number of animals with positive test

**Negative Predictive Value = ___ D__**

**D + C**

= Number of animals with negative test classified as treatment success

Number of animals with negative test

**RRTF = A/(A+B)**

**C/(C+D)**

= Number of animals with positive test classified as treatment failure

_______________Number of animals with positive test____________

Number of animals with negative test classified as treatment failure

Number of animals with negative test

**Susceptible vs. Non-susceptible**

**Sensitivity =** Number of animals with susceptible isolates classified as treatment successes

Number of animals that are classified as treatment successes

**Specificity =** Number of animals with non-susceptible isolates classified as treatment failures

Number of animals that are classified as treatment failures

**PPV =** Number of animals with susceptible isolates classified as treatment successes

Number of animals with susceptible isolates

**NPV =** Number of animals with non-susceptible isolates classified as treatment failures

Number of animals with non-susceptible isolates

**RRTF =** Number of animals with susceptible isolates classified as treatment failure

___________Number of animals with susceptible isolates_____________________

Number of animals with non-susceptible isolates classified as treatment failure

Number of animals with non-susceptible isolates

**Resistant vs. non-resistant**

**Sensitivity =** Number of animals with resistant isolates classified as treatment failures

Number of animals classified as treatment failures

**Specificity =** Number of animals with non-resistant isolates classified as treatment successes

Number of animals classified as treatment successes

**PPV =** Number of animals with resistant isolates classified as treatment failure

Number of animals with resistant isolates

**NPV =** Number of animals with non-resistant isolates classified treatment successes

Number of animals with non-resistant isolates.

**RRTF =** Number of animals with resistant isolates classified treatment failure

_________________Number of animals with resistant isolates______________

Number of animals with non-resistant isolates classified as treatment failure

Number of animals with non-resistant isolates
