# Supplementary material for: Limitations of bacterial culture, viral PCR, and tulathromycin susceptibility from upper respiratory tract samples in predicting clinical outcome of tulathromycin control or treatment of bovine respiratory disease in high-risk feeder heifers
Source: PLoS One. 2022 Feb 10;17(2):e0247213. doi: 10.1371/journal.pone.0247213 (PMC8830659; doi:10.1371/journal.pone.0247213)
Supplement: S4 Appendix — (DOCX) [file pone.0247213.s004.docx]

**S4 Appendix.**

| Antimicrobial Class | Antimicrobial Agent | Bacterial  Species | (S) | (I,R) | (S,I) | (R) |
| --- | --- | --- | --- | --- | --- | --- |
| Macrolide | Tulathromycin | *Mannheimia haemolytica*  *Pasteurella multocida* | <16ug/mL | >16ug/mL | <64ug/mL | >64ug/mL |
