# Supplementary material for: Limitations of bacterial culture, viral PCR, and tulathromycin susceptibility from upper respiratory tract samples in predicting clinical outcome of tulathromycin control or treatment of bovine respiratory disease in high-risk feeder heifers
Source: PLoS One. 2022 Feb 10;17(2):e0247213. doi: 10.1371/journal.pone.0247213 (PMC8830659; doi:10.1371/journal.pone.0247213)
Supplement: S1 File — (PDF) [file pone.0247213.s005.pdf]

PHASE IV ALL SPECIES STUDY BOVINE
STUDY: A131R-US-13-231
SUMMARY AND ANALYSIS

Addendum Section	Addendum Pages
PHASE IV ALL SPECIES STUDY BOVINE	
STUDY: A131R-US-13-231	
TREATMENT SUCCESS RATE	
SUMMARY BY ANIMAL	1-57
SUMMARY OF BACTERIAL ISOLATES	58-61
SUMMARY OF BACTERIAL ISOLATES BY TREATMENT SUCCESS	62-65
SUMMARY OF VIRAL EXPOSURE AT FIRST PULL	66-69
SUMMARY OF VIRAL EXPOSURE BY TREATMENT SUCCESS AT FIRST PULL	70-73
SUMMARY OF TIME OF ARRIVAL ON STUDY	74
SUMMARY OF TIME OF ARRIVAL ON STUDY BY TREATMENT SUCCESS	75
SUMMARY OF DAY 0 BODY WEIGHT BY TREATMENT SUCCESS	76
SUMMARY OF MIC BY ANIMAL	77-102
SUMMARY BY RESISTANCE OF BACTERIAL ISOLATES AND PERIOD	103
SENSITIVITY, SPECIFICITY, POSITIVE PREDICTIVE VALUE AND NEGATIVE PREDICTIVE VALUE	104
SUMMARY BY TIME OF ARRIVAL ON STUDY, RESISTANCE OF BACTERIAL ISOLATES AND PERIOD	105-107
SUMMARY BY VIRAL EXPOSURE AND RESISTANCE OF BACTERIAL ISOLATES AT FIRST PULL	108-111
SUMMARY BY SUSCEPTIBILITY OF BACTERIAL ISOLATES AND PERIOD	112
SENSITIVITY, SPECIFICITY, POSITIVE PREDICTIVE VALUE AND NEGATIVE PREDICTIVE VALUE	113
SUMMARY BY TIME OF ARRIVAL ON STUDY, SUSCEPTIBILITY OF BACTERIAL ISOLATES AND PERIOD	114-116
SUMMARY BY VIRAL EXPOSURE AND SUSCEPTIBILITY OF BACTERIAL ISOLATES AT FIRST PULL	117-120
SUMMARY OF MIC BY PERIOD	121

PHASE IV ALL SPECIES STUDY BOVINE
STUDY: A131R-US-13-231
TREATMENT SUCCESS RATE
SUMMARY BY ANIMAL

treatment=T01 test material=DRAXXIN period=Arrival

animal	day of study	day 0 date	day 0 weight (LBS)	histophilus somni	mannheimia haemolytica	mycoplasma bovis	pasteurella multocida	day of BRD	score	temperature (F)	day of removal	brd related	BRD mortality	treatment success [1]
1	0	06MAR15	512	N	N	N	N							YES
10	0	06MAR15	474	N	N	N	N							YES
100	0	13MAR15	508	N	N	N	N	9	1	103.7	13	BRD		NO
1000	0	28MAR15	466	N	N	N	N							YES
1001	0	28MAR15	521	N	N	N	N							YES
1002	0	28MAR15	436	N	N	N	N							YES
1003	0	28MAR15	490	N	N	N	N							YES
1004	0	28MAR15	540	N	N	N	N	19	2	106.3				NO
1005	0	28MAR15	565	N	N	N	N							YES
1006	0	28MAR15	450	N	N	N	N	12	1	104.7				NO
1007	0	28MAR15	478	N	N	N	N							YES
1008	0	28MAR15	515	N	N	N	N							YES
1009	0	28MAR15	471	N	N	N	N	25	2	106.0	28	BRD		NO
101	0	13MAR15	556	N	Y	N	N	10	1	105.5	18	BRD		NO
1010	0	28MAR15	483	N	N	N	N							YES
1011	0	28MAR15	487	N	Y	N	N	24	1	105.9				NO
1012	0	28MAR15	505	N	N	N	N							YES
1013	0	28MAR15	440	N	N	Y	N							YES
1014	0	28MAR15	488	N	N	N	N							YES
1015	0	28MAR15	480	N	N	N	N							YES
1016	0	28MAR15	468	N	Y	N	N	37	1	105.5	40	BRD		NO
1017	0	28MAR15	515	N	N	N	N							YES
1018	0	28MAR15	522	N	N	N	Y							YES
1019	0	28MAR15	536	N	N	N	N							YES
102	0	13MAR15	473	N	N	N	N	7	1	105.3				NO
1020	0	28MAR15	551	N	N	N	N							YES
1021	0	28MAR15	513	N	N	N	N							YES
1022	0	28MAR15	434	N	N	N	N							YES
1023	0	28MAR15	552	N	N	N	N							YES
1024	0	28MAR15	484	N	N	N	N							YES
1025	0	28MAR15	525	N	N	N	N							YES
1026	0	28MAR15	454	N	N	N	N							YES
1027	0	28MAR15	457	N	Y	N	Y							YES
1028	0	28MAR15	424	N	N	N	N							YES

DATA HAVE NOT BEEN VERIFIED

[1] Did not qualify for BRD re-treatment, not a BRD mortality and not removed for non-BRD reasons

PHASE IV ALL SPECIES STUDY BOVINE
STUDY: A131R-US-13-231
TREATMENT SUCCESS RATE
SUMMARY BY ANIMAL

treatment=T01 test material=DRAXXIN period=Arrival

animal	day of study	day 0 date	day 0 weight (LBS)	histophilus somni	mannheimia haemolytica	mycoplasma bovis	pasteurella multocida	day of BRD	score	temperature (F)	day of removal	brd related	BRD mortality	treatment success [1]
1029	0	28MAR15	442	N	N	N	N							YES
103	0	13MAR15	577	N	N	N	Y							YES
1030	0	28MAR15	533	N	N	N	N	11	1	105.6	19	BRD		NO
1031	0	28MAR15	429	N	N	N	N							YES
1032	0	28MAR15	470	N	N	N	N	14	1	106.2				NO
1033	0	28MAR15	487	Y	N	N	N	16	1	105.4				NO
1034	0	28MAR15	497	N	Y	N	N	37	1	103.6				NO
1035	0	28MAR15	456	N	N	N	N	11	1	104.3	18	BRD		NO
1036	0	28MAR15	439	N	N	N	N	8	1	106.5	14	BRD		NO
1037	0	28MAR15	437	N	N	N	N	11	1	105.9	14	BRD		NO
1038	0	28MAR15	471	N	N	N	N							YES
1039	0	28MAR15	446	N	N	N	N							YES
104	0	13MAR15	548	N	N	N	N	12	1	104.6				NO
1040	0	28MAR15	574	N	N	N	N							YES
105	0	13MAR15	523	N	N	N	N	7	1	104.9	15	BRD		NO
106	0	13MAR15	484	N	N	N	N							YES
107	0	13MAR15	518	N	N	N	N							YES
108	0	13MAR15	453	N	N	N	N	8	1	103.7	35	BRD		NO
109	0	13MAR15	440	N	N	N	N							YES
11	0	06MAR15	438	N	N	N	Y	10	1	103.5				NO
110	0	13MAR15	456	N	N	N	Y	9	1	104.9	17	BRD		NO
111	0	13MAR15	553	N	N	N	N							YES
112	0	13MAR15	551	N	Y	N	N	11	1	105.3				NO
113	0	13MAR15	543	N	N	N	N	12	1	107.0	19	BRD		NO
114	0	13MAR15	559	N	N	N	N	14	1	104.4				NO
115	0	13MAR15	493	N	Y	N	N	7	1	104.6	13	BRD		NO
116	0	13MAR15	455	N	N	N	N	7	1	103.6				NO
117	0	13MAR15	447	N	N	N	Y	12	1	103.6				NO
118	0	13MAR15	570	N	N	N	N	7	1	106.4				NO
119	0	13MAR15	545	N	N	N	Y							YES
12	0	06MAR15	447	N	N	N	N	7	1	103.5				NO
120	0	13MAR15	507	N	N	N	N	15	1	108.5				NO
121	0	13MAR15	460	N	N	N	N							YES
122	0	13MAR15	514	N	N	N	N							YES

DATA HAVE NOT BEEN VERIFIED

[1] Did not qualify for BRD re-treatment, not a BRD mortality and not removed for non-BRD reasons

PHASE IV ALL SPECIES STUDY BOVINE
STUDY: A131R-US-13-231
TREATMENT SUCCESS RATE
SUMMARY BY ANIMAL

treatment=T01 test material=DRAXXIN period=Arrival

animal	day of study	day 0 date	day 0 weight (LBS)	histophilus somni	mannheimia haemolytica	mycoplasma bovis	pasteurella multocida	day of BRD	score	temperature (F)	day of removal	brd related	BRD mortality	treatment success [1]
123	0	13MAR15	549	N	N	N	N							YES
124	0	13MAR15	536	N	N	N	N	28	1	103.5				NO
125	0	13MAR15	469	N	N	N	N							YES
126	0	13MAR15	450	N	N	N	N							YES
127	0	13MAR15	440	N	N	N	N	12	1	104.5	39	BRD		NO
128	0	13MAR15	510	N	Y	N	N	14	1	105.2	23	BRD		NO
129	0	13MAR15	525	N	N	N	N							YES
13	0	06MAR15	502	N	N	N	N							YES
130	0	13MAR15	590	N	N	N	N	11	1	105.4	13	BRD		NO
131	0	13MAR15	524	N	N	N	Y							YES
132	0	13MAR15	533	N	N	N	N	14	1	106.3	34	BRD		NO
133	0	13MAR15	532	N	N	N	N	11	1	107.1				NO
134	0	13MAR15	482	N	N	N	N							YES
135	0	13MAR15	474	N	N	N	N							YES
136	0	13MAR15	549	N	N	N	Y	10	1	103.9				NO
137	0	13MAR15	540	N	N	N	N	12	1	105.0	26	BRD		NO
138	0	13MAR15	452	N	N	N	N							YES
139	0	13MAR15	498	N	N	N	N	12	1	105.8	17	BRD		NO
14	0	06MAR15	536	N	N	N	N							YES
140	0	13MAR15	476	N	Y	N	N	12	1	104.5	28	BRD		NO
141	0	13MAR15	539	N	N	N	N	11	1	103.5	40	BRD		NO
142	0	13MAR15	462	N	N	N	N	12	1	103.5				NO
143	0	13MAR15	445	N	N	N	N							YES
144	0	13MAR15	514	N	Y	N	N	10	1	105.1				NO
145	0	13MAR15	565	N	N	N	N							YES
146	0	13MAR15	467	N	N	N	N	13	1	104.2				NO
147	0	13MAR15	502	N	N	N	N	17	2	105.2				NO
148	0	13MAR15	563	N	N	N	N	7	1	103.6	16	BRD		NO
149	0	13MAR15	510	N	Y	N	N	12	1	103.5				NO
15	0	06MAR15	530	N	N	Y	N							YES
151	0	13MAR15	550	N	N	N	N	7	1	104.0				NO
152	0	13MAR15	444	N	N	N	N							YES
153	0	13MAR15	523	N	N	N	N							YES
154	0	13MAR15	533	N	N	N	N	13	1	106.1				NO

DATA HAVE NOT BEEN VERIFIED

[1] Did not qualify for BRD re-treatment, not a BRD mortality and not removed for non-BRD reasons

PHASE IV ALL SPECIES STUDY BOVINE
STUDY: A131R-US-13-231
TREATMENT SUCCESS RATE
SUMMARY BY ANIMAL

treatment=T01 test material=DRAXXIN period=Arrival

animal	day of study	day 0 date	day 0 weight (LBS)	histophilus somni	mannheimia haemolytica	mycoplasma bovis	pasteurella multocida	day of BRD	score	temperature (F)	day of removal	brd related	BRD mortality	treatment success [1]
155	0	13MAR15	475	N	N	N	N	13	1	103.7				NO
156	0	13MAR15	431	N	N	N	N	8	1	103.8				NO
158	0	13MAR15	512	N	Y	N	N							YES
159	0	13MAR15	541	N	N	N	N	11	1	106.4	36	BRD		NO
16	0	06MAR15	460	N	N	N	N							YES
160	0	13MAR15	549	N	Y	N	Y	7	1	103.5	14	BRD		NO
161	0	13MAR15	560	N	N	N	Y							YES
162	0	13MAR15	503	N	N	N	N							YES
163	0	13MAR15	499	N	Y	N	N	11	1	106.2	18	BRD		NO
164	0	13MAR15	429	N	N	N	Y	12	1	103.7	20	BRD		NO
165	0	13MAR15	501	N	Y	Y	N	8	1	104.7	17	BRD		NO
166	0	13MAR15	568	N	Y	N	N	8	1	104.7	16	BRD		NO
167	0	13MAR15	569	N	Y	Y	N	18	1	106.5				NO
168	0	13MAR15	480	N	N	N	N							YES
169	0	13MAR15	548	N	N	N	N	13	1	104.7				NO
17	0	06MAR15	486	N	N	N	N	14	2	101.3				NO
170	0	13MAR15	487	N	N	N	N							YES
171	0	13MAR15	524	N	Y	N	N	10	1	104.0	16	BRD		NO
172	0	13MAR15	448	N	N	N	N	9	1	103.9	18	BRD		NO
173	0	13MAR15	520	N	Y	Y	N							YES
174	0	13MAR15	436	N	N	N	Y	12	1	104.0				NO
175	0	13MAR15	546	N	N	N	N	8	1	105.5	11	BRD		NO
176	0	13MAR15	450	N	N	N	N							YES
177	0	13MAR15	507	N	N	N	Y	8	1	104.8	30	BRD		NO
178	0	13MAR15	479	N	Y	N	N							YES
179	0	13MAR15	542	N	N	N	Y	17	1	106.0	35	BRD		NO
18	0	06MAR15	505	N	Y	N	N							YES
180	0	13MAR15	552	N	N	N	N							YES
181	0	13MAR15	478	N	N	N	N	17	1	108.2				NO
182	0	13MAR15	511	N	N	N	N							YES
183	0	13MAR15	500	N	Y	N	N							YES
184	0	13MAR15	517	N	N	N	N	7	1	104.5	13	BRD		NO
185	0	13MAR15	534	N	N	N	N							YES
186	0	13MAR15	490	N	N	N	N							YES

DATA HAVE NOT BEEN VERIFIED

[1] Did not qualify for BRD re-treatment, not a BRD mortality and not removed for non-BRD reasons

PHASE IV ALL SPECIES STUDY BOVINE
STUDY: A131R-US-13-231
TREATMENT SUCCESS RATE
SUMMARY BY ANIMAL

treatment=T01 test material=DRAXXIN period=Arrival

animal	day of study	day 0 date	day 0 weight (LBS)	histophilus somni	mannheimia haemolytica	mycoplasma bovis	pasteurella multocida	day of BRD	score	temperature (F)	day of removal	brd related	BRD mortality	treatment success [1]
187	0	13MAR15	471	N	N	N	N	7	1	104.7				NO
188	0	13MAR15	540	N	N	N	N	34	1	105.0				NO
189	0	13MAR15	508	N	Y	N	N							YES
19	0	06MAR15	465	N	N	N	N							YES
190	0	13MAR15	556	N	Y	N	N							YES
191	0	13MAR15	436	N	N	N	N	13	1	103.8	21	BRD		NO
192	0	13MAR15	474	N	N	N	N	7	1	105.0	10	BRD		NO
193	0	13MAR15	486	N	N	N	N							YES
194	0	13MAR15	515	N	N	N	N							YES
195	0	13MAR15	546	N	N	N	N	14	1	105.0				NO
196	0	13MAR15	535	N	N	N	N							YES
197	0	13MAR15	527	N	N	N	N							YES
198	0	13MAR15	489	N	Y	N	N							YES
199	0	13MAR15	554	N	N	N	Y							YES
2	0	06MAR15	436	N	Y	N	N							YES
20	0	06MAR15	438	N	N	N	Y	11	1	103.7	24	BRD		NO
200	0	13MAR15	477	N	N	N	N	40	1	104.6				NO
201	0	13MAR15	470	N	N	N	N	7	1	104.7				NO
202	0	13MAR15	575	N	N	N	N	18	1	104.0				NO
203	0	13MAR15	496	N	Y	N	N	25	2	104.6	30	BRD		NO
204	0	13MAR15	616	N	N	N	N							YES
205	0	13MAR15	475	N	Y	N	N							YES
206	0	13MAR15	476	N	N	N	N							YES
207	0	13MAR15	514	N	N	N	N							YES
208	0	13MAR15	507	N	N	N	N							YES
209	0	13MAR15	545	N	Y	N	N	7	1	103.8	12	BRD		NO
21	0	06MAR15	436	N	N	N	N							YES
210	0	13MAR15	426	N	N	N	N							YES
211	0	13MAR15	512	N	Y	N	Y							YES
212	0	13MAR15	483	N	N	N	N	13	1	106.2				NO
213	0	13MAR15	546	N	N	N	N	25	2	106.0				NO
214	0	13MAR15	475	N	N	N	Y	11	1	106.3				NO
215	0	13MAR15	482	N	N	N	N	7	1	104.0	19	BRD		NO
216	0	13MAR15	530	N	N	N	N							YES

DATA HAVE NOT BEEN VERIFIED

[1] Did not qualify for BRD re-treatment, not a BRD mortality and not removed for non-BRD reasons

treatment=T01 test material=DRAXXIN period=Arrival

animal	day of study	day 0 date	day 0 weight (LBS)	histophilus somni	mannheimia haemolytica	mycoplasma bovis	pasteurella multocida	day of BRD	score	temperature (F)	day of removal	brd related	BRD mortality	treatment success [1]
217	0	13MAR15	516	N	N	N	N	7	1	103.5				NO
218	0	13MAR15	473	N	N	N	N							YES
219	0	13MAR15	584	N	N	N	N							YES
22	0	06MAR15	466	N	N	N	N	13	1	104.6	20	BRD		NO
220	0	13MAR15	478	N	N	N	N							YES
221	0	13MAR15	486	N	N	N	N	19	2	105.3				NO
222	0	13MAR15	490	N	N	Y	Y	8	1	105.6				NO
223	0	13MAR15	473	N	N	N	N							YES
224	0	13MAR15	540	N	N	N	N							YES
225	0	13MAR15	494	N	Y	N	N							YES
226	0	13MAR15	525	N	N	N	N							YES
227	0	13MAR15	499	N	N	N	N	18	1	103.5				NO
228	0	13MAR15	442	N	N	N	N							YES
229	0	13MAR15	542	N	N	N	Y							YES
23	0	06MAR15	570	N	N	N	N							YES
230	0	13MAR15	570	N	N	N	Y							YES
231	0	13MAR15	461	N	N	N	N							YES
232	0	13MAR15	508	N	N	N	N	14	1	104.0	20	BRD		NO
233	0	13MAR15	450	N	N	N	N							YES
234	0	13MAR15	482	N	N	N	N	20	1	104.3	29	BRD		NO
235	0	13MAR15	416	N	N	N	N							YES
236	0	13MAR15	531	N	N	N	N							YES
237	0	13MAR15	547	N	Y	N	N							YES
238	0	13MAR15	446	N	N	N	Y	7	1	103.5	17	BRD		NO
239	0	13MAR15	448	N	N	N	N	14	2	104.0				NO
24	0	06MAR15	562	N	N	N	N	7	1	103.5	12	BRD		NO
240	0	13MAR15	570	N	N	N	N							YES
241	0	13MAR15	473	N	N	N	N							YES
242	0	13MAR15	458	N	N	N	N							YES
243	0	13MAR15	427	N	N	N	N							YES
244	0	13MAR15	507	N	Y	N	N							YES
245	0	13MAR15	546	N	N	N	N							YES
246	0	13MAR15	558	N	N	N	N							YES
247	0	13MAR15	534	N	N	N	N	8	1	103.5	27	BRD		NO

DATA HAVE NOT BEEN VERIFIED

[1] Did not qualify for BRD re-treatment, not a BRD mortality and not removed for non-BRD reasons

PHASE IV ALL SPECIES STUDY BOVINE
STUDY: A131R-US-13-231
TREATMENT SUCCESS RATE
SUMMARY BY ANIMAL

treatment=T01 test material=DRAXXIN period=Arrival

animal	day of study	day 0 date	day 0 weight (LBS)	histophilus somni	mannheimia haemolytica	mycoplasma bovis	pasteurella multocida	day of BRD	score	temperature (F)	day of removal	brd related	BRD mortality	treatment success [1]
248	0	13MAR15	532	N	Y	N	N	17	2	107.0				NO
249	0	13MAR15	491	N	N	N	N							YES
25	0	06MAR15	568	N	N	N	N							YES
250	0	13MAR15	500	N	N	N	N	25	2	106.5				NO
251	0	13MAR15	508	N	N	N	N							YES
252	0	13MAR15	457	N	N	N	Y							YES
253	0	13MAR15	503	N	N	N	Y							YES
254	0	13MAR15	480	N	N	N	N							YES
255	0	13MAR15	450	N	N	N	N	18	1	105.5				NO
256	0	13MAR15	540	N	N	N	N	29	1	104.5				NO
257	0	13MAR15	568	N	N	N	N							YES
258	0	13MAR15	480	N	N	N	N	18	1	106.2	28	BRD		NO
259	0	13MAR15	510	N	N	N	N							YES
26	0	06MAR15	502	N	N	N	N	7	1	104.6				NO
260	0	13MAR15	557	N	N	N	N							YES
261	0	13MAR15	574	N	N	N	N							YES
262	0	13MAR15	500	N	N	N	N	12	2	104.7	19	BRD		NO
263	0	13MAR15	512	N	Y	N	N							YES
264	0	13MAR15	533	N	N	N	N	21	1	107.0	28	BRD		NO
265	0	13MAR15	435	N	N	N	N	20	1	106.1	30	BRD		NO
266	0	13MAR15	552	N	N	N	N	21	1	105.5				NO
268	0	13MAR15	560	N	Y	N	N	18	1	104.5				NO
269	0	13MAR15	556	N	N	N	N							YES
27	0	06MAR15	477	N	N	N	N							YES
270	0	13MAR15	485	N	N	N	N							YES
271	0	13MAR15	527	N	N	N	N	21	1	105.8	28	BRD		NO
272	0	13MAR15	463	N	N	N	Y	33	1	105.2				NO
273	0	13MAR15	511	N	N	N	N							YES
274	0	13MAR15	556	N	N	N	N							YES
275	0	13MAR15	492	N	N	N	N	23	1	106.6	25	BRD		NO
276	0	13MAR15	501	N	N	N	Y							YES
277	0	13MAR15	480	N	N	N	N	19	1	106.3				NO
278	0	13MAR15	564	N	N	N	N	21	1	104.0				NO
279	0	13MAR15	539	N	N	N	N							YES

DATA HAVE NOT BEEN VERIFIED

[1] Did not qualify for BRD re-treatment, not a BRD mortality and not removed for non-BRD reasons

PHASE IV ALL SPECIES STUDY BOVINE
STUDY: A131R-US-13-231
TREATMENT SUCCESS RATE
SUMMARY BY ANIMAL

treatment=T01 test material=DRAXXIN period=Arrival

animal	day of study	day 0 date	day 0 weight (LBS)	histophilus somni	mannheimia haemolytica	mycoplasma bovis	pasteurella multocida	day of BRD	score	temperature (F)	day of removal	brd related	BRD mortality	treatment success [1]
28	0	06MAR15	506	N	N	N	Y							YES
280	0	13MAR15	591	N	N	N	N	17	1	106.4	24	BRD		NO
281	0	13MAR15	521	N	N	N	N							YES
282	0	13MAR15	501	N	N	N	N							YES
283	0	13MAR15	510	N	Y	N	Y							YES
284	0	13MAR15	536	N	N	N	N							YES
285	0	13MAR15	577	N	N	N	N							YES
286	0	13MAR15	576	N	N	N	Y							YES
287	0	13MAR15	577	N	Y	N	N	17	1	105.7				NO
288	0	13MAR15	453	N	N	N	N	21	1	106.4				NO
289	0	13MAR15	406	N	Y	N	N							YES
29	0	06MAR15	503	N	N	N	N	11	1	104.6				NO
290	0	13MAR15	463	N	N	N	N							YES
291	0	20MAR15	480	N	N	N	N	13	1	105.6	20	BRD		NO
292	0	20MAR15	441	N	Y	N	N	12	1	103.7	24	BRD		NO
293	0	20MAR15	493	N	N	N	N							YES
294	0	20MAR15	493	N	Y	N	N							YES
295	0	20MAR15	470	N	N	N	N	7	1	104.9	22	BRD		NO
296	0	20MAR15	456	N	N	N	N							YES
297	0	20MAR15	463	N	N	N	N							YES
298	0	20MAR15	558	N	N	N	N							YES
299	0	20MAR15	445	N	N	N	N							YES
3	0	06MAR15	470	N	N	N	N	13	1	103.9				NO
30	0	06MAR15	524	N	N	N	N	7	2	102.6	12	BRD		NO
300	0	20MAR15	455	N	N	N	N	19	1	104.6	35	BRD		NO
301	0	20MAR15	510	N	N	N	N	7	2	103.3	15	BRD		NO
302	0	20MAR15	487	N	N	N	N	7	1	104.9				NO
303	0	20MAR15	508	N	N	N	N							YES
305	0	20MAR15	478	N	N	N	N							YES
306	0	20MAR15	478	N	N	N	N							YES
307	0	20MAR15	420	N	N	N	N	7	1	103.5				NO
308	0	20MAR15	520	N	N	N	N							YES
309	0	20MAR15	444	N	N	N	N	7	1	104.7				NO
31	0	06MAR15	527	N	N	N	N							YES

DATA HAVE NOT BEEN VERIFIED

[1] Did not qualify for BRD re-treatment, not a BRD mortality and not removed for non-BRD reasons

PHASE IV ALL SPECIES STUDY BOVINE
STUDY: A131R-US-13-231
TREATMENT SUCCESS RATE
SUMMARY BY ANIMAL

treatment=T01 test material=DRAXXIN period=Arrival

animal	day of study	day 0 date	day 0 weight (LBS)	histophilus somni	mannheimia haemolytica	mycoplasma bovis	pasteurella multocida	day of BRD	score	temperature (F)	day of removal	brd related	BRD mortality	treatment success [1]
310	0	20MAR15	457	N	N	N	N							YES
311	0	20MAR15	405	N	N	N	N	12	1	106.7	19	BRD		NO
312	0	20MAR15	484	N	N	N	N	18	1	107.0				NO
313	0	20MAR15	423	N	N	N	Y							YES
314	0	20MAR15	441	N	N	N	Y							YES
315	0	20MAR15	425	N	N	N	N	25	1	104.3				NO
316	0	20MAR15	471	N	N	N	N							YES
317	0	20MAR15	463	N	N	N	N							YES
318	0	20MAR15	455	N	N	N	N	25	1	104.8	32	BRD		NO
319	0	20MAR15	439	N	N	N	N	11	1	103.6	18	BRD		NO
32	0	06MAR15	475	N	N	N	N							YES
320	0	20MAR15	459	N	N	N	Y	14	1	107.0				NO
321	0	20MAR15	489	N	N	N	N							YES
322	0	20MAR15	455	N	N	N	N							YES
323	0	20MAR15	430	N	N	N	N	14	1	104.2				NO
324	0	20MAR15	562	N	N	N	N							YES
325	0	20MAR15	506	N	N	N	N							YES
326	0	20MAR15	482	N	N	N	N							YES
327	0	20MAR15	502	N	N	N	N	7	1	104.6				NO
328	0	20MAR15	451	N	N	N	N							YES
329	0	20MAR15	465	N	N	N	N	15	1	103.5				NO
33	0	06MAR15	510	N	N	N	N	12	1	104.7	21	BRD		NO
330	0	20MAR15	454	N	N	N	Y	8	1	106.1	12	BRD		NO
331	0	20MAR15	458	N	N	N	N							YES
332	0	20MAR15	450	N	N	N	N	9	1	104.1				NO
333	0	20MAR15	426	N	N	N	N							YES
334	0	20MAR15	510	N	N	N	N							YES
335	0	20MAR15	480	N	N	N	N							YES
336	0	20MAR15	494	N	N	N	N							YES
337	0	20MAR15	457	N	N	N	Y							YES
338	0	20MAR15	502	N	N	N	N							YES
339	0	20MAR15	475	N	N	N	Y							YES
34	0	06MAR15	529	N	N	N	N	8	1	107.3	15	BRD		NO
340	0	20MAR15	496	N	N	N	Y							YES

DATA HAVE NOT BEEN VERIFIED

[1] Did not qualify for BRD re-treatment, not a BRD mortality and not removed for non-BRD reasons

PHASE IV ALL SPECIES STUDY BOVINE
STUDY: A131R-US-13-231
TREATMENT SUCCESS RATE
SUMMARY BY ANIMAL

treatment=T01 test material=DRAXXIN period=Arrival

animal	day of study	day 0 date	day 0 weight (LBS)	histophilus somni	mannheimia haemolytica	mycoplasma bovis	pasteurella multocida	day of BRD	score	temperature (F)	day of removal	brd related	BRD mortality	treatment success [1]
341	0	20MAR15	471	N	N	N	N	10	1	103.6	16	BRD		NO
342	0	20MAR15	459	N	N	N	N	7	1	103.8				NO
343	0	20MAR15	557	N	N	N	N	18	1	104.2	26	BRD		NO
344	0	20MAR15	468	N	N	N	N							YES
345	0	20MAR15	552	N	N	N	N							YES
346	0	20MAR15	422	N	N	N	N	13	1	104.4	23	BRD		NO
347	0	20MAR15	496	N	N	N	N							YES
348	0	20MAR15	509	N	N	N	N							YES
349	0	20MAR15	441	N	N	N	N	16	2	106.4	27	BRD		NO
35	0	06MAR15	495	N	N	N	N	16	1	104.2				NO
350	0	20MAR15	471	N	N	N	N	21	2	106.7				NO
352	0	20MAR15	435	N	N	N	Y							YES
353	0	20MAR15	457	N	N	N	N	19	1	105.3				NO
354	0	20MAR15	459	N	N	N	N							YES
355	0	20MAR15	475	N	N	N	N							YES
356	0	20MAR15	404	N	N	N	N	7	1	103.8	18	BRD		NO
357	0	20MAR15	429	N	N	N	N	11	1	106.6	15	BRD	YES	NO
358	0	20MAR15	482	N	N	N	N							YES
359	0	20MAR15	484	N	N	N	N							YES
36	0	06MAR15	530	N	N	N	N							YES
360	0	20MAR15	436	N	N	N	N							YES
361	0	20MAR15	456	N	N	N	Y							YES
362	0	20MAR15	506	N	N	N	N							YES
363	0	20MAR15	465	N	Y	N	N							YES
364	0	20MAR15	507	N	N	N	N							YES
365	0	20MAR15	457	N	N	N	N	19	1	105.1	27	BRD		NO
366	0	20MAR15	434	N	Y	N	N	10	1	107.4	17	BRD		NO
367	0	20MAR15	474	N	N	N	N	14	1	104.6	17	BRD		NO
368	0	20MAR15	448	N	N	N	N							YES
369	0	20MAR15	507	N	N	N	N	15	1	105.3				NO
37	0	06MAR15	564	N	N	N	N	10	1	105.6				NO
370	0	20MAR15	452	N	N	N	N	12	1	106.8	16	BRD		NO
371	0	20MAR15	513	N	N	N	N							YES
372	0	20MAR15	478	N	N	N	Y							YES

DATA HAVE NOT BEEN VERIFIED

[1] Did not qualify for BRD re-treatment, not a BRD mortality and not removed for non-BRD reasons

PHASE IV ALL SPECIES STUDY BOVINE
STUDY: A131R-US-13-231
TREATMENT SUCCESS RATE
SUMMARY BY ANIMAL

treatment=T01 test material=DRAXXIN period=Arrival

animal	day of study	day 0 date	day 0 weight (LBS)	histophilus somni	mannheimia haemolytica	mycoplasma bovis	pasteurella multocida	day of BRD	score	temperature (F)	day of removal	brd related	BRD mortality	treatment success [1]
373	0	20MAR15	449	N	N	N	N	20	1	104.7				NO
374	0	20MAR15	475	N	N	N	N	13	1	107.3	15	BRD	YES	NO
375	0	20MAR15	401	N	N	N	N							YES
376	0	20MAR15	450	N	N	N	N							YES
377	0	20MAR15	436	N	N	N	N	23	1	106.4				NO
378	0	20MAR15	495	N	N	N	N	10	1	104.8	17	BRD		NO
379	0	20MAR15	475	N	N	N	N	10	1	104.6	16	BRD		NO
38	0	06MAR15	464	N	Y	N	N	11	1	103.9				NO
380	0	20MAR15	411	N	N	N	Y	15	1	106.3	18	BRD		NO
381	0	20MAR15	466	N	N	N	N							YES
382	0	20MAR15	460	N	N	N	N							YES
383	0	20MAR15	483	N	N	N	N	9	1	103.7				NO
384	0	20MAR15	478	N	N	N	N	8	1	103.8				NO
385	0	20MAR15	484	N	N	N	N	9	1	105.1				NO
386	0	20MAR15	546	N	N	N	N	12	1	103.8				NO
387	0	20MAR15	526	N	N	N	N							YES
388	0	20MAR15	471	N	N	N	N	15	1	104.8	19	BRD		NO
389	0	20MAR15	586	N	N	N	N							YES
39	0	06MAR15	542	N	N	N	N	12	1	104.9	29	BRD		NO
390	0	20MAR15	573	N	N	N	N	16	1	106.0				NO
391	0	20MAR15	597	N	N	N	Y	14	1	107.0	21	BRD		NO
392	0	20MAR15	583	N	N	N	N							YES
393	0	20MAR15	538	N	N	N	N							YES
394	0	20MAR15	510	N	N	N	N							YES
395	0	20MAR15	614	N	N	N	N	7	1	103.7				NO
396	0	20MAR15	536	N	N	N	N	19	1	103.7				NO
397	0	20MAR15	500	N	N	N	Y							YES
398	0	20MAR15	481	N	N	N	N							YES
399	0	20MAR15	551	N	N	N	Y							YES
4	0	06MAR15	501	N	N	N	N							YES
40	0	06MAR15	506	N	N	N	Y							YES
400	0	20MAR15	530	N	N	N	N							YES
401	0	20MAR15	517	N	N	N	N							YES
402	0	20MAR15	642	N	N	N	N							YES

DATA HAVE NOT BEEN VERIFIED

[1] Did not qualify for BRD re-treatment, not a BRD mortality and not removed for non-BRD reasons

PHASE IV ALL SPECIES STUDY BOVINE
STUDY: A131R-US-13-231
TREATMENT SUCCESS RATE
SUMMARY BY ANIMAL

treatment=T01 test material=DRAXXIN period=Arrival

animal	day of study	day 0 date	day 0 weight (LBS)	histophilus somni	mannheimia haemolytica	mycoplasma bovis	pasteurella multocida	day of BRD	score	temperature (F)	day of removal	brd related	BRD mortality	treatment success [1]
403	0	20MAR15	540	N	N	N	N							YES
404	0	20MAR15	550	N	N	N	N							YES
405	0	20MAR15	532	N	Y	N	N							YES
406	0	20MAR15	533	N	N	N	N	17	2	106.7				NO
407	0	20MAR15	537	N	N	N	N	11	1	103.5				NO
408	0	20MAR15	527	N	N	N	N							YES
409	0	20MAR15	518	N	N	N	N							YES
41	0	06MAR15	467	N	N	N	N							YES
410	0	20MAR15	505	N	N	N	N	10	1	104.1	17	BRD		NO
411	0	20MAR15	538	N	N	N	N	12	2	104.9				NO
412	0	20MAR15	500	N	N	N	N							YES
413	0	20MAR15	567	N	N	N	N							YES
415	0	20MAR15	521	N	N	N	N	10	1	104.7	17	BRD		NO
416	0	20MAR15	489	N	N	N	N	13	1	107.1				NO
417	0	20MAR15	515	N	N	N	N	7	1	103.5	15	BRD		NO
418	0	20MAR15	511	N	N	N	N							YES
419	0	20MAR15	511	N	N	N	Y							YES
42	0	06MAR15	478	N	N	N	N							YES
420	0	20MAR15	552	N	N	N	N							YES
421	0	20MAR15	570	N	N	N	N							YES
422	0	20MAR15	514	N	N	N	N	15	1	104.2	18	BRD		NO
423	0	20MAR15	560	N	N	N	N							YES
424	0	20MAR15	526	N	N	N	N							YES
425	0	20MAR15	556	N	N	N	Y							YES
426	0	20MAR15	528	N	N	N	N							YES
427	0	20MAR15	550	N	N	N	N							YES
428	0	20MAR15	515	N	N	N	N	14	1	104.4				NO
429	0	20MAR15	522	N	N	N	N							YES
43	0	06MAR15	518	N	N	N	N							YES
430	0	20MAR15	571	N	N	N	N							YES
431	0	20MAR15	537	N	N	N	N	19	1	103.9				NO
432	0	20MAR15	526	N	N	N	N	8	1	103.8				NO
433	0	20MAR15	546	N	N	N	N	12	1	103.9	15	BRD		NO
434	0	20MAR15	500	N	N	N	Y							YES

DATA HAVE NOT BEEN VERIFIED

[1] Did not qualify for BRD re-treatment, not a BRD mortality and not removed for non-BRD reasons

PHASE IV ALL SPECIES STUDY BOVINE
STUDY: A131R-US-13-231
TREATMENT SUCCESS RATE
SUMMARY BY ANIMAL

treatment=T01 test material=DRAXXIN period=Arrival

animal	day of study	day 0 date	day 0 weight (LBS)	histophilus somni	mannheimia haemolytica	mycoplasma bovis	pasteurella multocida	day of BRD	score	temperature (F)	day of removal	brd related	BRD mortality	treatment success [1]
435	0	20MAR15	586	N	N	N	N							YES
436	0	20MAR15	537	N	N	N	N	19	1	106.1				NO
437	0	20MAR15	544	N	Y	N	Y	11	1	106.1	30	BRD		NO
438	0	20MAR15	573	N	N	N	N							YES
439	0	20MAR15	552	N	N	N	N	7	1	106.7				NO
44	0	06MAR15	450	N	N	N	N	10	1	104.5	19	BRD		NO
440	0	20MAR15	508	N	N	N	N	7	1	103.5	20	BRD		NO
441	0	20MAR15	506	N	N	N	N	19	1	105.8				NO
442	0	20MAR15	472	N	N	N	N							YES
443	0	20MAR15	578	N	N	N	N							YES
444	0	20MAR15	494	N	Y	N	N							YES
445	0	20MAR15	551	N	N	N	N	12	1	105.6				NO
446	0	20MAR15	490	N	N	N	N							YES
447	0	20MAR15	515	N	N	N	N	12	1	104.5	20	BRD		NO
448	0	20MAR15	533	N	N	N	N	10	1	103.8	15	BRD		NO
449	0	20MAR15	560	N	Y	N	N							YES
45	0	06MAR15	430	N	N	N	N	7	2	103.1	20	BRD		NO
450	0	20MAR15	541	N	N	N	N	11	1	103.6	18	BRD		NO
451	0	20MAR15	512	N	N	N	N							YES
452	0	20MAR15	530	N	N	N	Y	23	1	106.0	25	BRD		NO
453	0	20MAR15	538	N	N	N	N	7	1	103.9				NO
454	0	20MAR15	523	N	N	N	N	11	1	104.1	18	BRD		NO
455	0	20MAR15	544	N	N	N	N							YES
456	0	20MAR15	548	N	N	N	N	10	1	105.1	15	BRD		NO
457	0	20MAR15	518	N	N	N	N							YES
458	0	20MAR15	552	N	N	N	N	12	1	105.2	20	BRD		NO
459	0	20MAR15	612	N	N	N	N							YES
46	0	06MAR15	470	N	N	N	Y	7	2	103.5				NO
460	0	20MAR15	508	N	N	N	N	24	1	104.3				NO
461	0	20MAR15	543	N	N	N	N	9	1	104.0				NO
462	0	20MAR15	524	N	N	N	N	15	1	104.9				NO
463	0	20MAR15	587	N	N	N	N	15	1	103.9				NO
464	0	20MAR15	497	N	N	N	Y							YES
465	0	20MAR15	482	N	N	N	N							YES

DATA HAVE NOT BEEN VERIFIED

[1] Did not qualify for BRD re-treatment, not a BRD mortality and not removed for non-BRD reasons

PHASE IV ALL SPECIES STUDY BOVINE
STUDY: A131R-US-13-231
TREATMENT SUCCESS RATE
SUMMARY BY ANIMAL

treatment=T01 test material=DRAXXIN period=Arrival

animal	day of study	day 0 date	day 0 weight (LBS)	histophilus somni	mannheimia haemolytica	mycoplasma bovis	pasteurella multocida	day of BRD	score	temperature (F)	day of removal	brd related	BRD mortality	treatment success [1]
466	0	20MAR15	491	N	N	N	N							YES
467	0	20MAR15	560	N	N	N	N	21	2	103.6				NO
468	0	20MAR15	495	N	N	N	N							YES
469	0	23MAR15	432	N	N	N	N	8	1	103.7	17	BRD		NO
47	0	06MAR15	496	N	N	N	N							YES
470	0	23MAR15	497	N	N	N	N	15	1	106.5	18	BRD		NO
471	0	23MAR15	529	Y	N	N	N	16	2	106.1	18	BRD		NO
472	0	23MAR15	514	N	Y	N	N							YES
473	0	23MAR15	450	N	Y	N	N	11	1	104.1	18	BRD		NO
474	0	23MAR15	502	N	N	N	Y	12	1	104.9				NO
475	0	23MAR15	484	N	N	N	N							YES
476	0	23MAR15	532	N	Y	N	N	10	1	106.9	15	BRD		NO
477	0	23MAR15	445	N	N	N	N	8	1	103.7	18	BRD		NO
478	0	23MAR15	555	N	N	N	Y	11	1	103.8	32	BRD		NO
479	0	23MAR15	538	N	N	N	N							YES
48	0	06MAR15	488	N	N	N	N							YES
480	0	23MAR15	565	N	N	N	N							YES
481	0	23MAR15	568	N	N	N	Y							YES
482	0	23MAR15	538	N	N	N	N							YES
483	0	23MAR15	496	N	N	N	N							YES
484	0	23MAR15	537	N	N	N	N	14	1	105.7				NO
485	0	23MAR15	504	N	Y	N	N	7	1	104.1	16	BRD		NO
486	0	23MAR15	484	N	N	N	N	15	1	106.9	18	BRD		NO
487	0	23MAR15	579	N	Y	N	N	36	1	103.5	40	BRD		NO
488	0	23MAR15	430	N	Y	N	N	10	1	105.0	22	BRD		NO
489	0	23MAR15	503	N	N	N	N	7	1	105.3	15	BRD		NO
49	0	06MAR15	484	N	N	N	N	21	1	104.0				NO
490	0	23MAR15	516	N	N	N	Y							YES
491	0	23MAR15	482	N	N	N	N	7	1	103.9	22	BRD		NO
492	0	23MAR15	537	N	N	N	N	10	1	105.6	14	BRD		NO
493	0	23MAR15	505	N	Y	N	N	9	1	106.1	17	BRD		NO
494	0	23MAR15	428	N	N	N	N	7	1	103.8	19	BRD		NO
495	0	23MAR15	517	N	Y	N	Y							YES
496	0	23MAR15	542	N	N	N	N	12	1	103.6				NO

DATA HAVE NOT BEEN VERIFIED

[1] Did not qualify for BRD re-treatment, not a BRD mortality and not removed for non-BRD reasons

PHASE IV ALL SPECIES STUDY BOVINE
STUDY: A131R-US-13-231
TREATMENT SUCCESS RATE
SUMMARY BY ANIMAL

treatment=T01 test material=DRAXXIN period=Arrival

animal	day of study	day 0 date	day 0 weight (LBS)	histophilus somni	mannheimia haemolytica	mycoplasma bovis	pasteurella multocida	day of BRD	score	temperature (F)	day of removal	brd related	BRD mortality	treatment success [1]
497	0	23MAR15	439	N	N	N	N	9	1	104.2	20	BRD		NO
498	0	23MAR15	559	N	N	N	N							YES
499	0	23MAR15	420	N	N	N	N	7	1	104.3	16	BRD		NO
5	0	06MAR15	472	N	N	N	Y	10	1	103.5	20	BRD		NO
50	0	06MAR15	526	N	N	N	N							YES
500	0	23MAR15	495	N	Y	N	N							YES
501	0	23MAR15	551	N	N	N	N	12	1	105.7				NO
502	0	23MAR15	476	N	N	N	N							YES
503	0	23MAR15	512	N	N	N	Y	24	1	106.3				NO
504	0	23MAR15	463	N	N	N	N							YES
505	0	23MAR15	486	N	Y	N	Y	7	1	105.3	15	BRD		NO
506	0	23MAR15	539	N	N	N	N	15	1	104.8				NO
507	0	23MAR15	540	N	N	N	N							YES
508	0	23MAR15	485	N	N	N	Y	15	1	106.2	18	BRD		NO
509	0	23MAR15	457	N	N	N	N	8	1	104.3	21	BRD		NO
51	0	06MAR15	514	N	N	N	N	10	1	103.9	17	BRD		NO
510	0	23MAR15	575	N	N	N	N	16	2	106.0	24	BRD		NO
511	0	23MAR15	485	N	N	N	N	11	1	104.0	16	BRD		NO
512	0	23MAR15	573	N	N	N	Y	10	1	103.8				NO
513	0	23MAR15	527	N	N	N	Y	21	1	103.6				NO
514	0	23MAR15	471	N	N	N	N							YES
515	0	23MAR15	540	N	Y	N	N							YES
516	0	23MAR15	493	N	N	N	N							YES
517	0	23MAR15	480	N	N	N	N	8	1	104.9	15	BRD		NO
518	0	23MAR15	518	N	N	N	N	10	1	104.5				NO
519	0	23MAR15	506	N	N	N	N	15	1	106.5				NO
52	0	06MAR15	509	N	N	N	N	10	2		19	BRD		NO
520	0	23MAR15	545	N	N	N	N	7	1	103.5	15	BRD		NO
521	0	23MAR15	459	N	N	N	N							YES
522	0	23MAR15	478	N	N	N	Y	9	1	103.8	27	BRD		NO
523	0	23MAR15	446	N	N	N	N							YES
524	0	23MAR15	500	N	N	N	N	15	1	104.9	22	BRD		NO
525	0	23MAR15	537	N	N	N	Y							YES
526	0	23MAR15	568	N	N	N	N	12	1	104.0				NO

DATA HAVE NOT BEEN VERIFIED

[1] Did not qualify for BRD re-treatment, not a BRD mortality and not removed for non-BRD reasons

PHASE IV ALL SPECIES STUDY BOVINE
STUDY: A131R-US-13-231
TREATMENT SUCCESS RATE
SUMMARY BY ANIMAL

treatment=T01 test material=DRAXXIN period=Arrival

animal	day of study	day 0 date	day 0 weight (LBS)	histophilus somni	mannheimia haemolytica	mycoplasma bovis	pasteurella multocida	day of BRD	score	temperature (F)	day of removal	brd related	BRD mortality	treatment success [1]
527	0	23MAR15	517	N	N	N	N							YES
528	0	23MAR15	577	N	N	N	N							YES
529	0	23MAR15	495	N	N	N	N							YES
53	0	06MAR15	547	N	N	N	N							YES
530	0	23MAR15	507	N	N	N	N							YES
531	0	23MAR15	490	N	Y	N	Y	15	1	106.2	22	BRD		NO
532	0	23MAR15	496	N	N	N	N	13	1	105.9				NO
533	0	23MAR15	481	N	N	N	N							YES
534	0	23MAR15	500	N	N	N	N							YES
535	0	23MAR15	454	N	N	N	N							YES
536	0	23MAR15	518	N	N	N	N							YES
537	0	23MAR15	491	N	N	N	N	17	2	106.3	22	BRD		NO
538	0	23MAR15	517	N	N	N	N							YES
539	0	23MAR15	527	N	N	N	N							YES
54	0	06MAR15	511	N	N	N	N							YES
540	0	23MAR15	520	N	N	N	N							YES
541	0	23MAR15	502	N	Y	N	N	12	1	105.6	19	BRD		NO
542	0	23MAR15	473	N	Y	N	N	19	1	103.6				NO
543	0	23MAR15	510	N	Y	N	N	11	1	104.6	19	BRD		NO
544	0	23MAR15	515	N	N	N	N							YES
545	0	23MAR15	484	N	N	N	N							YES
546	0	23MAR15	493	N	N	N	N							YES
547	0	23MAR15	433	N	N	N	N	36	1	103.6				NO
548	0	23MAR15	480	N	N	N	N	11	1	104.0	19	BRD		NO
549	0	23MAR15	469	N	N	N	N	14	1	106.3	29	BRD		NO
55	0	06MAR15	470	N	N	N	Y	10	1	105.9				NO
550	0	23MAR15	542	N	N	N	N	26	1	105.7				NO
551	0	23MAR15	490	N	Y	N	Y	36	1	105.2				NO
552	0	23MAR15	469	N	N	N	N	8	1	104.4	15	BRD		NO
553	0	23MAR15	543	N	Y	N	N							YES
554	0	23MAR15	493	N	N	N	Y							YES
555	0	23MAR15	570	N	N	N	N							YES
556	0	23MAR15	577	N	Y	N	N	7	1	106.7				NO
557	0	23MAR15	542	N	Y	N	N							YES

DATA HAVE NOT BEEN VERIFIED

[1] Did not qualify for BRD re-treatment, not a BRD mortality and not removed for non-BRD reasons

treatment=T01 test material=DRAXXIN period=Arrival

animal	day of study	day 0 date	day 0 weight (LBS)	histophilus somni	mannheimia haemolytica	mycoplasma bovis	pasteurella multocida	day of BRD	score	temperature (F)	day of removal	brd related	BRD mortality	treatment success [1]
558	0	23MAR15	602	N	N	N	N							YES
559	0	23MAR15	489	N	N	N	N							YES
56	0	06MAR15	514	N	N	N	N							YES
560	0	23MAR15	555	N	N	N	N							YES
561	0	23MAR15	569	N	Y	N	N	20	1	105.6				NO
562	0	23MAR15	642	N	N	N	N							YES
563	0	23MAR15	601	N	N	N	Y							YES
564	0	23MAR15	481	N	N	N	N	11	2	102.1				NO
565	0	23MAR15	621	N	N	N	N							YES
566	0	23MAR15	524	N	N	N	N							YES
567	0	23MAR15	527	N	N	N	N	13	1	105.4				NO
568	0	23MAR15	522	N	N	N	N							YES
569	0	23MAR15	593	N	N	N	N							YES
57	0	06MAR15	496	N	N	N	N	9	2	103.7	15	BRD		NO
570	0	23MAR15	633	N	N	N	N	10	1	104.3				NO
571	0	23MAR15	501	N	N	N	N	16	1	104.4				NO
572	0	23MAR15	624	N	N	N	N							YES
574	0	23MAR15	514	N	N	N	Y							YES
575	0	23MAR15	526	N	N	N	N							YES
576	0	23MAR15	563	N	Y	N	N	7	1	103.8				NO
577	0	23MAR15	563	N	N	N	N							YES
578	0	23MAR15	505	N	Y	N	N							YES
579	0	23MAR15	578	N	Y	N	Y							YES
58	0	06MAR15	557	N	N	N	Y							YES
580	0	23MAR15	571	N	N	N	N							YES
581	0	23MAR15	571	N	N	N	N							YES
582	0	23MAR15	554	N	Y	N	N	29	1	106.1				NO
583	0	23MAR15	566	N	N	N	N	8	1	103.6	20	BRD		NO
584	0	23MAR15	582	N	N	Y	N							YES
585	0	23MAR15	540	N	N	N	N	16	1	107.0	27	BRD		NO
586	0	23MAR15	490	N	N	N	N							YES
587	0	23MAR15	583	N	N	N	N							YES
588	0	23MAR15	488	N	Y	N	Y							YES
589	0	23MAR15	578	N	N	N	N							YES

DATA HAVE NOT BEEN VERIFIED

[1] Did not qualify for BRD re-treatment, not a BRD mortality and not removed for non-BRD reasons

treatment=T01 test material=DRAXXIN period=Arrival

animal	day of study	day 0 date	day 0 weight (LBS)	histophilus somni	mannheimia haemolytica	mycoplasma bovis	pasteurella multocida	day of BRD	score	temperature (F)	day of removal	brd related	BRD mortality	treatment success [1]
59	0	06MAR15	499	N	N	N	Y							YES
590	0	23MAR15	599	N	N	N	N							YES
591	0	23MAR15	504	N	N	N	N							YES
592	0	23MAR15	561	N	N	N	N							YES
593	0	23MAR15	580	N	N	N	Y							YES
594	0	23MAR15	607	N	N	N	N							YES
595	0	23MAR15	619	N	Y	N	Y	16	1	104.1				NO
596	0	23MAR15	600	N	N	N	N							YES
597	0	23MAR15	535	N	Y	N	Y							YES
598	0	23MAR15	581	N	N	N	N							YES
599	0	23MAR15	602	N	N	N	N							YES
6	0	06MAR15	513	N	N	N	N							YES
60	0	06MAR15	482	N	N	N	N							YES
600	0	23MAR15	578	N	N	N	N							YES
601	0	23MAR15	554	N	N	N	Y	8	1	103.5	31	BRD		NO
602	0	23MAR15	565	N	N	N	N							YES
603	0	23MAR15	515	N	N	N	N	7	1	104.8	19	BRD		NO
604	0	23MAR15	564	N	N	N	N							YES
605	0	23MAR15	488	N	N	N	N							YES
606	0	23MAR15	572	N	Y	N	Y							YES
607	0	23MAR15	538	N	N	N	Y							YES
608	0	23MAR15	540	N	N	N	Y							YES
609	0	23MAR15	586	N	Y	N	N							YES
61	0	06MAR15	541	N	N	N	N							YES
610	0	23MAR15	498	N	N	N	N							YES
611	0	23MAR15	580	N	N	N	N							YES
612	0	23MAR15	549	N	Y	N	N							YES
613	0	23MAR15	571	N	N	N	N							YES
614	0	23MAR15	580	N	Y	N	N							YES
615	0	23MAR15	510	N	N	N	N	7	1	103.7	16	BRD		NO
616	0	23MAR15	546	N	Y	N	N							YES
617	0	23MAR15	600	N	Y	N	N	28	1	105.1				NO
618	0	23MAR15	516	N	Y	N	N	7	1	104.8				NO
619	0	23MAR15	588	N	Y	N	N	16	1	104.7	24	BRD		NO

DATA HAVE NOT BEEN VERIFIED

[1] Did not qualify for BRD re-treatment, not a BRD mortality and not removed for non-BRD reasons

treatment=T01 test material=DRAXXIN period=Arrival

animal	day of study	day 0 date	day 0 weight (LBS)	histophilus somni	mannheimia haemolytica	mycoplasma bovis	pasteurella multocida	day of BRD	score	temperature (F)	day of removal	brd related	BRD mortality	treatment success [1]
62	0	06MAR15	444	N	N	N	N	24	2	103.4				NO
620	0	23MAR15	547	N	Y	N	N							YES
621	0	23MAR15	595	N	N	N	N							YES
622	0	23MAR15	570	N	N	N	N	13	1	103.5				NO
623	0	23MAR15	591	N	Y	N	N							YES
624	0	23MAR15	548	N	N	N	Y							YES
625	0	23MAR15	497	N	N	N	N	11	1	104.0				NO
626	0	23MAR15	490	N	N	N	Y	16	1	104.3				NO
627	0	23MAR15	510	N	N	N	N							YES
628	0	23MAR15	599	N	N	N	N							YES
629	0	23MAR15	560	N	N	N	N							YES
63	0	06MAR15	407	N	N	N	N							YES
630	0	23MAR15	550	N	Y	N	N	24	1	104.2				NO
631	0	23MAR15	568	N	Y	N	N	7	1	104.6	14	BRD		NO
632	0	23MAR15	558	N	N	N	Y							YES
633	0	23MAR15	613	N	N	N	N							YES
634	0	23MAR15	534	N	Y	N	N							YES
635	0	23MAR15	557	N	N	N	N							YES
636	0	23MAR15	573	N	N	N	N	8	1	104.5				NO
637	0	23MAR15	526	N	Y	N	N							YES
638	0	23MAR15	550	N	N	N	N							YES
639	0	23MAR15	566	N	N	N	N							YES
64	0	06MAR15	484	N	N	N	N	10	2	103.1				NO
640	0	23MAR15	505	N	N	N	N							YES
641	0	23MAR15	588	N	Y	N	N							YES
642	0	23MAR15	490	N	N	N	N							YES
643	0	23MAR15	538	N	N	N	N							YES
644	0	25MAR15	402	N	N	N	N	15	1	104.0	22	BRD		NO
645	0	25MAR15	429	N	N	N	N	14	1	103.5				NO
646	0	25MAR15	394	N	N	N	N							YES
647	0	25MAR15	317	N	N	N	N							YES
648	0	25MAR15	480	N	N	N	N							YES
649	0	25MAR15	440	N	N	N	N							YES
65	0	06MAR15	503	N	N	N	Y	11	1	103.6	17	BRD		NO

DATA HAVE NOT BEEN VERIFIED

[1] Did not qualify for BRD re-treatment, not a BRD mortality and not removed for non-BRD reasons

PHASE IV ALL SPECIES STUDY BOVINE
STUDY: A131R-US-13-231
TREATMENT SUCCESS RATE
SUMMARY BY ANIMAL

treatment=T01 test material=DRAXXIN period=Arrival

animal	day of study	day 0 date	day 0 weight (LBS)	histophilus somni	mannheimia haemolytica	mycoplasma bovis	pasteurella multocida	day of BRD	score	temperature (F)	day of removal	brd related	BRD mortality	treatment success [1]
650	0	25MAR15	341	N	N	N	N							YES
651	0	25MAR15	410	N	N	N	N	16	1	104.9				NO
652	0	25MAR15	423	N	N	N	N							YES
653	0	25MAR15	372	N	N	N	N	13	1	106.3	20	BRD		NO
654	0	25MAR15	399	N	N	N	N	14	1	105.7	16	BRD		NO
655	0	25MAR15	393	N	N	N	N							YES
656	0	25MAR15	445	N	N	N	N	16	1	105.6	35	BRD		NO
657	0	25MAR15	411	N	N	N	N							YES
658	0	25MAR15	425	N	N	N	N							YES
659	0	25MAR15	428	N	N	N	N							YES
66	0	06MAR15	545	N	N	N	N	7	2	105.1	9	BRD		NO
660	0	25MAR15	430	N	N	N	N							YES
661	0	25MAR15	443	N	N	N	N							YES
662	0	25MAR15	422	N	N	N	N							YES
663	0	25MAR15	428	Y	N	N	N							YES
664	0	25MAR15	404	N	N	N	N							YES
665	0	25MAR15	437	N	N	N	Y	20	1	103.5				NO
666	0	25MAR15	425	N	N	N	N							YES
667	0	25MAR15	436	N	N	N	N							YES
668	0	25MAR15	422	N	N	N	N							YES
669	0	25MAR15	405	N	Y	N	N							YES
67	0	06MAR15	546	N	N	N	N							YES
670	0	25MAR15	370	N	N	N	N							YES
671	0	25MAR15	468	N	N	N	N							YES
672	0	25MAR15	450	N	N	N	N							YES
673	0	25MAR15	418	N	N	N	N							YES
674	0	25MAR15	422	N	N	N	N							YES
675	0	25MAR15	455	N	N	N	N	16	1	105.8				NO
676	0	25MAR15	462	N	N	N	N							YES
677	0	25MAR15	423	N	N	N	N							YES
678	0	25MAR15	346	N	N	N	N							YES
679	0	25MAR15	471	N	N	N	N							YES
68	0	06MAR15	590	N	Y	N	N							YES
680	0	25MAR15	378	N	N	N	N	16	1	104.5				NO

DATA HAVE NOT BEEN VERIFIED

[1] Did not qualify for BRD re-treatment, not a BRD mortality and not removed for non-BRD reasons

PHASE IV ALL SPECIES STUDY BOVINE
STUDY: A131R-US-13-231
TREATMENT SUCCESS RATE
SUMMARY BY ANIMAL

treatment=T01 test material=DRAXXIN period=Arrival

animal	day of study	day 0 date	day 0 weight (LBS)	histophilus somni	mannheimia haemolytica	mycoplasma bovis	pasteurella multocida	day of BRD	score	temperature (F)	day of removal	brd related	BRD mortality	treatment success [1]
681	0	25MAR15	430	N	N	N	N							YES
682	0	25MAR15	414	N	N	N	N							YES
683	0	25MAR15	465	N	N	N	N							YES
684	0	25MAR15	355	N	N	N	N							YES
685	0	25MAR15	427	Y	N	N	N							YES
686	0	25MAR15	479	N	N	N	N							YES
687	0	25MAR15	418	Y	N	N	Y							YES
688	0	25MAR15	485	N	N	N	N							YES
689	0	25MAR15	427	N	N	N	Y							YES
69	0	06MAR15	497	N	Y	N	N	10	1	105.3	18	BRD		NO
690	0	25MAR15	403	N	N	N	N							YES
691	0	25MAR15	422	N	N	N	N							YES
692	0	25MAR15	413	N	N	N	N	17	1	103.8				NO
693	0	25MAR15	433	N	N	N	N	15	1	104.0	22	BRD		NO
694	0	25MAR15	407	N	Y	N	N							YES
695	0	25MAR15	428	N	N	N	N	13	1	104.8				NO
696	0	25MAR15	384	N	N	N	N							YES
697	0	25MAR15	436	N	N	N	N							YES
698	0	25MAR15	448	N	N	N	N							YES
699	0	25MAR15	385	N	N	N	N							YES
7	0	06MAR15	481	N	N	N	N	7	2	104.2				NO
70	0	06MAR15	540	N	N	N	N	10	1	103.6				NO
700	0	25MAR15	454	N	N	N	N							YES
701	0	25MAR15	426	N	N	N	N							YES
702	0	25MAR15	382	N	N	N	N							YES
703	0	25MAR15	426	N	N	N	N							YES
704	0	25MAR15	390	N	N	N	N							YES
705	0	25MAR15	400	N	N	N	N	22	1	103.8				NO
706	0	25MAR15	424	N	N	N	N	15	1	107.9	22	BRD		NO
707	0	25MAR15	402	N	N	N	N							YES
708	0	25MAR15	407	N	N	N	N	16	1	106.2				NO
709	0	25MAR15	452	N	N	N	N							YES
71	0	06MAR15	538	N	N	N	N							YES
710	0	25MAR15	398	N	N	N	N							YES

DATA HAVE NOT BEEN VERIFIED

[1] Did not qualify for BRD re-treatment, not a BRD mortality and not removed for non-BRD reasons

treatment=T01 test material=DRAXXIN period=Arrival

animal	day of study	day 0 date	day 0 weight (LBS)	histophilus somni	mannheimia haemolytica	mycoplasma bovis	pasteurella multocida	day of BRD	score	temperature (F)	day of removal	brd related	BRD mortality	treatment success [1]
711	0	25MAR15	430	N	N	N	Y							YES
712	0	25MAR15	355	N	N	N	N	22	1	104.9				NO
713	0	25MAR15	409	N	N	N	N	15	1	107.0	22	BRD		NO
714	0	25MAR15	395	Y	N	N	N							YES
715	0	25MAR15	449	N	N	N	N							YES
716	0	25MAR15	408	N	Y	N	N							YES
717	0	25MAR15	493	N	N	N	N							YES
718	0	25MAR15	451	N	N	N	N							YES
72	0	06MAR15	484	N	N	N	N	11	1	104.4	17	BRD		NO
720	0	25MAR15	426	N	N	N	N							YES
721	0	25MAR15	412	N	N	N	N	15	1	107.7	22	BRD		NO
722	0	25MAR15	378	N	N	N	N							YES
723	0	25MAR15	380	N	N	N	N							YES
724	0	25MAR15	380	N	N	N	N	35	1	105.3				NO
725	0	25MAR15	373	N	N	N	N							YES
726	0	25MAR15	415	N	N	N	N							YES
727	0	25MAR15	401	N	N	N	N							YES
728	0	25MAR15	394	N	N	N	N	12	1	105.8	21	BRD		NO
729	0	25MAR15	371	N	N	N	Y							YES
73	0	06MAR15	508	N	N	N	N	12	1	104.9	19	BRD		NO
730	0	25MAR15	363	N	N	N	N							YES
731	0	25MAR15	410	N	N	N	N							YES
733	0	25MAR15	382	N	N	N	Y							YES
734	0	25MAR15	458	N	N	N	N							YES
735	0	25MAR15	449	N	N	N	N							YES
736	0	25MAR15	431	N	N	N	N							YES
737	0	25MAR15	340	N	N	N	N	14	1	105.8				NO
738	0	25MAR15	410	N	N	N	N							YES
739	0	25MAR15	364	N	N	N	N							YES
74	0	06MAR15	420	N	N	N	Y	7	1	103.9				NO
740	0	25MAR15	428	N	N	N	N							YES
741	0	25MAR15	443	N	N	N	N							YES
742	0	25MAR15	440	N	N	N	N							YES
743	0	25MAR15	424	N	Y	Y	N							YES

DATA HAVE NOT BEEN VERIFIED

[1] Did not qualify for BRD re-treatment, not a BRD mortality and not removed for non-BRD reasons

PHASE IV ALL SPECIES STUDY BOVINE
STUDY: A131R-US-13-231
TREATMENT SUCCESS RATE
SUMMARY BY ANIMAL

treatment=T01 test material=DRAXXIN period=Arrival

animal	day of study	day 0 date	day 0 weight (LBS)	histophilus somni	mannheimia haemolytica	mycoplasma bovis	pasteurella multocida	day of BRD	score	temperature (F)	day of removal	brd related	BRD mortality	treatment success [1]
744	0	25MAR15	440	N	N	N	N							YES
745	0	25MAR15	450	N	N	N	N	14	1	106.6				NO
746	0	25MAR15	432	N	N	N	Y							YES
747	0	25MAR15	442	N	N	N	N							YES
748	0	25MAR15	434	N	N	N	N	14	1	105.1				NO
749	0	25MAR15	443	N	N	N	N							YES
75	0	06MAR15	534	N	N	N	N							YES
750	0	25MAR15	460	N	N	N	N							YES
751	0	25MAR15	400	N	N	N	N							YES
752	0	25MAR15	434	N	N	N	N	14	1	106.7	24	BRD		NO
753	0	25MAR15	404	N	N	N	N	9	1	105.7				NO
754	0	25MAR15	349	N	N	N	N	18	1	106.7				NO
755	0	25MAR15	403	N	N	N	N							YES
757	0	25MAR15	425	N	N	N	N							YES
758	0	25MAR15	435	N	N	N	N							YES
759	0	25MAR15	429	N	N	N	N							YES
760	0	26MAR15	511	N	N	N	N	13	1	105.1	34	BRD		NO
761	0	26MAR15	498	N	N	N	N							YES
762	0	26MAR15	466	N	N	N	N							YES
763	0	26MAR15	503	N	N	Y	N							YES
764	0	26MAR15	514	N	N	N	N							YES
765	0	26MAR15	504	N	N	N	Y	13	1	106.8				NO
766	0	26MAR15	485	N	N	N	N	18	1	105.6				NO
767	0	26MAR15	508	N	N	N	Y							YES
768	0	26MAR15	456	N	N	N	N							YES
769	0	26MAR15	475	N	N	N	N	30	1	104.8				NO
77	0	06MAR15	485	N	N	N	N							YES
770	0	26MAR15	525	N	Y	N	N							YES
771	0	26MAR15	492	N	N	N	N	10	1	103.5				NO
772	0	26MAR15	558	N	N	N	N	18	1	105.7	25	BRD		NO
773	0	26MAR15	454	N	N	N	N	10	1	104.5				NO
774	0	26MAR15	498	N	N	N	N							YES
775	0	26MAR15	466	N	N	N	N	8	1	106.5	12	BRD		NO
776	0	26MAR15	492	N	N	N	N							YES

DATA HAVE NOT BEEN VERIFIED

[1] Did not qualify for BRD re-treatment, not a BRD mortality and not removed for non-BRD reasons

PHASE IV ALL SPECIES STUDY BOVINE
STUDY: A131R-US-13-231
TREATMENT SUCCESS RATE
SUMMARY BY ANIMAL

treatment=T01 test material=DRAXXIN period=Arrival

animal	day of study	day 0 date	day 0 weight (LBS)	histophilus somni	mannheimia haemolytica	mycoplasma bovis	pasteurella multocida	day of BRD	score	temperature (F)	day of removal	brd related	BRD mortality	treatment success [1]
777	0	26MAR15	519	N	N	N	N							YES
778	0	26MAR15	569	N	N	N	N							YES
779	0	26MAR15	467	N	N	N	N	13	1	104.5	20	BRD		NO
78	0	06MAR15	481	N	N	N	N							YES
780	0	26MAR15	483	N	N	N	N							YES
781	0	26MAR15	483	N	N	N	N							YES
782	0	26MAR15	432	N	N	N	N							YES
783	0	26MAR15	505	N	N	N	N	14	2	107.3	21	BRD		NO
784	0	26MAR15	509	N	N	N	N							YES
785	0	26MAR15	464	N	N	N	N							YES
786	0	26MAR15	499	N	N	N	N	17	2	105.9	33	BRD		NO
787	0	26MAR15	464	N	N	N	N							YES
788	0	26MAR15	569	N	N	N	N							YES
789	0	26MAR15	574	N	N	N	N							YES
79	0	06MAR15	500	N	N	N	N							YES
790	0	26MAR15	448	N	N	N	N							YES
791	0	26MAR15	518	N	N	N	N							YES
792	0	26MAR15	507	N	N	N	N	10	1	104.3	15	BRD		NO
793	0	26MAR15	532	N	N	N	N	9	1	105.8				NO
794	0	26MAR15	462	N	N	N	Y							YES
795	0	26MAR15	535	N	N	N	N	16	2	106.9				NO
796	0	26MAR15	439	N	Y	N	N							YES
797	0	26MAR15	635	N	N	N	N							YES
798	0	26MAR15	545	N	N	N	N	8	1	105.0	24	BRD		NO
799	0	26MAR15	500	N	N	N	N	11	2	105.2	18	BRD		NO
8	0	06MAR15	522	N	N	N	N	14	1	104.6				NO
80	0	06MAR15	497	N	N	N	N	7	1	103.6	12	BRD		NO
800	0	26MAR15	510	N	N	N	N	12	1	104.7				NO
801	0	26MAR15	580	N	N	N	N							YES
802	0	26MAR15	487	N	N	N	N	7	1	104.8				NO
803	0	26MAR15	511	N	N	N	N							YES
804	0	26MAR15	562	N	N	N	N							YES
805	0	26MAR15	474	N	N	N	N	9	1	103.5	21	BRD		NO
806	0	26MAR15	482	N	N	N	N	11	1	105.6	19	BRD		NO

DATA HAVE NOT BEEN VERIFIED

[1] Did not qualify for BRD re-treatment, not a BRD mortality and not removed for non-BRD reasons

PHASE IV ALL SPECIES STUDY BOVINE
STUDY: A131R-US-13-231
TREATMENT SUCCESS RATE
SUMMARY BY ANIMAL

treatment=T01 test material=DRAXXIN period=Arrival

animal	day of study	day 0 date	day 0 weight (LBS)	histophilus somni	mannheimia haemolytica	mycoplasma bovis	pasteurella multocida	day of BRD	score	temperature (F)	day of removal	brd related	BRD mortality	treatment success [1]
807	0	26MAR15	520	N	N	N	N	7	1	104.9	14	BRD		NO
808	0	26MAR15	470	N	Y	N	Y							YES
809	0	26MAR15	513	N	N	N	N	13	1	104.0	17	BRD		NO
81	0	06MAR15	558	N	N	N	N							YES
810	0	26MAR15	628	N	N	N	N							YES
811	0	26MAR15	520	N	N	N	N							YES
812	0	26MAR15	514	N	N	N	N							YES
813	0	26MAR15	506	N	N	N	N							YES
814	0	26MAR15	500	N	N	N	N	26	1	106.1	33	BRD		NO
815	0	26MAR15	515	N	N	N	N	7	1	104.2	14	BRD		NO
816	0	26MAR15	510	N	N	N	N							YES
817	0	26MAR15	424	N	N	N	N							YES
818	0	26MAR15	450	N	N	N	N	8	1	105.7	13	BRD		NO
819	0	26MAR15	423	N	N	N	N	8	1	104.9	17	BRD		NO
82	0	06MAR15	541	N	N	N	N							YES
820	0	26MAR15	476	N	N	N	N							YES
821	0	26MAR15	418	N	N	N	N							YES
822	0	26MAR15	436	N	N	N	N							YES
823	0	26MAR15	507	N	N	N	N							YES
824	0	26MAR15	474	N	N	N	N	8	1	103.6	19	BRD		NO
825	0	26MAR15	539	N	N	N	N							YES
826	0	26MAR15	515	N	N	N	N							YES
827	0	26MAR15	493	N	N	N	N							YES
828	0	26MAR15	485	N	N	N	N							YES
829	0	26MAR15	493	N	N	N	N	13	1	105.8	15	BRD		NO
83	0	06MAR15	540	N	Y	N	N	11	1	103.6				NO
830	0	26MAR15	435	N	N	N	N							YES
831	0	26MAR15	455	N	N	N	N							YES
832	0	26MAR15	446	N	N	N	N	13	1	106.2	21	BRD		NO
834	0	26MAR15	520	N	N	N	N	7	1	104.8				NO
836	0	26MAR15	443	N	N	N	N	19	1	105.4	28	BRD		NO
837	0	26MAR15	541	N	N	N	N							YES
838	0	26MAR15	501	N	N	N	N	14	1	104.6	21	BRD		NO
839	0	26MAR15	467	N	N	N	N							YES

DATA HAVE NOT BEEN VERIFIED

[1] Did not qualify for BRD re-treatment, not a BRD mortality and not removed for non-BRD reasons

PHASE IV ALL SPECIES STUDY BOVINE
STUDY: A131R-US-13-231
TREATMENT SUCCESS RATE
SUMMARY BY ANIMAL

treatment=T01 test material=DRAXXIN period=Arrival

animal	day of study	day 0 date	day 0 weight (LBS)	histophilus somni	mannheimia haemolytica	mycoplasma bovis	pasteurella multocida	day of BRD	score	temperature (F)	day of removal	brd related	BRD mortality	treatment success [1]
84	0	06MAR15	460	N	Y	N	Y	7	2	103.6				NO
840	0	26MAR15	577	N	N	N	N							YES
841	0	26MAR15	510	N	N	N	N							YES
842	0	26MAR15	442	N	N	N	N	12	1	107.6				NO
843	0	26MAR15	636	N	N	N	N							YES
844	0	26MAR15	598	N	N	N	N							YES
845	0	26MAR15	480	N	N	N	N							YES
846	0	26MAR15	595	N	N	N	N							YES
847	0	26MAR15	437	N	N	N	N							YES
848	0	26MAR15	499	N	N	N	Y							YES
849	0	27MAR15	566	N	Y	N	N							YES
850	0	27MAR15	523	N	N	N	N							YES
851	0	27MAR15	482	N	N	N	N							YES
852	0	27MAR15	515	N	N	N	N	12	1	104.6	33	BRD		NO
853	0	27MAR15	528	N	N	N	N	15	2	105.1				NO
854	0	27MAR15	539	N	N	N	N	7	1	103.6				NO
855	0	27MAR15	522	N	N	N	N	32	1	103.7				NO
856	0	27MAR15	530	N	N	N	N	7	1	103.9				NO
857	0	27MAR15	556	N	N	N	N							YES
858	0	27MAR15	567	N	N	N	N							YES
859	0	27MAR15	523	N	N	N	N							YES
86	0	06MAR15	418	N	Y	N	N	13	1	104.9	19	BRD		NO
860	0	27MAR15	540	N	N	N	N							YES
861	0	27MAR15	508	N	N	Y	N							YES
863	0	27MAR15	535	N	N	N	N							YES
864	0	27MAR15	499	N	N	N	N							YES
865	0	27MAR15	464	N	N	N	N	11	1	105.8	18	BRD		NO
866	0	27MAR15	595	N	N	N	N							YES
867	0	27MAR15	523	N	N	N	N	9	1	107.8	14	BRD		NO
868	0	27MAR15	560	N	N	N	N	7	1	105.1	10	BRD	YES	NO
869	0	27MAR15	458	N	N	N	N							YES
87	0	06MAR15	444	N	Y	N	N	19	1	103.5				NO
870	0	27MAR15	492	N	N	N	N							YES
871	0	27MAR15	593	N	N	N	N							YES

DATA HAVE NOT BEEN VERIFIED

[1] Did not qualify for BRD re-treatment, not a BRD mortality and not removed for non-BRD reasons

treatment=T01 test material=DRAXXIN period=Arrival

animal	day of study	day 0 date	day 0 weight (LBS)	histophilus somni	mannheimia haemolytica	mycoplasma bovis	pasteurella multocida	day of BRD	score	temperature (F)	day of removal	brd related	BRD mortality	treatment success [1]
872	0	27MAR15	534	N	N	N	Y	7	1	104.2				NO
873	0	27MAR15	538	N	N	N	N	10	1	105.5	12	BRD		NO
874	0	27MAR15	563	N	N	N	N	7	1	106.3	10	BRD		NO
875	0	27MAR15	519	N	N	N	Y							YES
876	0	27MAR15	538	N	N	N	N	25	1	104.8	40	BRD		NO
877	0	27MAR15	542	N	N	N	N	9	1	104.1	19	BRD		NO
878	0	27MAR15	475	N	N	N	N	18	1	105.0				NO
879	0	27MAR15	471	N	Y	N	N							YES
88	0	06MAR15	479	N	N	N	N	18	1	106.8	20	BRD		NO
880	0	27MAR15	547	N	N	N	N							YES
881	0	27MAR15	510	N	N	N	N							YES
882	0	27MAR15	509	N	N	N	N							YES
883	0	27MAR15	481	N	N	N	N							YES
884	0	27MAR15	501	N	N	N	N							YES
885	0	27MAR15	524	N	N	N	N							YES
886	0	27MAR15	578	N	N	N	N							YES
887	0	27MAR15	534	N	N	N	N							YES
888	0	27MAR15	483	N	N	N	N	12	1	104.0	19	BRD		NO
889	0	27MAR15	560	N	N	N	N							YES
89	0	06MAR15	479	N	N	N	Y	7	2	102.4				NO
890	0	27MAR15	527	N	N	N	N							YES
891	0	27MAR15	594	N	N	N	N							YES
892	0	27MAR15	546	N	Y	N	N							YES
893	0	27MAR15	554	N	N	N	N	26	2	104.9	33	BRD		NO
894	0	27MAR15	538	N	N	N	N	25	1	105.3	37	BRD		NO
895	0	27MAR15	486	N	N	N	N	40	1	103.6				NO
896	0	27MAR15	582	N	N	N	N	32	1	103.7	40	BRD		NO
897	0	27MAR15	532	N	N	N	N							YES
898	0	27MAR15	519	N	N	N	N	8	1	103.8				NO
899	0	27MAR15	501	N	N	N	N							YES
9	0	06MAR15	526	N	N	N	N	7	1	104.2	17	BRD		NO
90	0	06MAR15	414	N	N	N	N	7	1	103.5				NO
900	0	27MAR15	576	N	N	N	N							YES
901	0	27MAR15	548	N	N	N	N	10	1	104.0				NO

DATA HAVE NOT BEEN VERIFIED

[1] Did not qualify for BRD re-treatment, not a BRD mortality and not removed for non-BRD reasons

PHASE IV ALL SPECIES STUDY BOVINE
STUDY: A131R-US-13-231
TREATMENT SUCCESS RATE
SUMMARY BY ANIMAL

treatment=T01 test material=DRAXXIN period=Arrival

animal	day of study	day 0 date	day 0 weight (LBS)	histophilus somni	mannheimia haemolytica	mycoplasma bovis	pasteurella multocida	day of BRD	score	temperature (F)	day of removal	brd related	BRD mortality	treatment success [1]
902	0	27MAR15	571	N	N	N	N							YES
903	0	27MAR15	545	N	N	N	N							YES
904	0	27MAR15	528	N	N	N	N	11	2	105.5	17	BRD		NO
905	0	27MAR15	534	N	N	N	N							YES
906	0	27MAR15	511	N	N	N	N							YES
907	0	27MAR15	575	N	N	N	N	33	1	106.2				NO
908	0	27MAR15	565	N	N	N	N							YES
909	0	27MAR15	531	N	N	N	N							YES
91	0	06MAR15	437	N	N	N	N							YES
910	0	27MAR15	498	N	Y	N	N							YES
911	0	27MAR15	540	N	N	N	N							YES
912	0	27MAR15	506	N	Y	N	N	10	1	104.3	18	BRD		NO
913	0	27MAR15	458	N	N	N	N	7	1	105.4				NO
914	0	27MAR15	540	N	N	N	N							YES
915	0	27MAR15	441	N	N	N	N	20	1	106.4	37	BRD		NO
916	0	27MAR15	500	N	N	N	N							YES
917	0	27MAR15	525	N	N	N	Y	27	1	103.5				NO
918	0	27MAR15	558	N	N	N	N	12	1	104.3	21	BRD		NO
919	0	27MAR15	528	N	N	N	N							YES
92	0	06MAR15	440	N	N	N	N							YES
920	0	27MAR15	587	N	N	N	N							YES
921	0	27MAR15	439	N	N	N	N							YES
922	0	27MAR15	477	N	N	N	N	12	2	103.8	14	BRD		NO
923	0	27MAR15	502	N	N	N	N							YES
924	0	27MAR15	514	N	N	N	N							YES
925	0	27MAR15	495	N	N	N	N							YES
926	0	27MAR15	428	N	N	N	N							YES
927	0	27MAR15	560	N	N	N	N							YES
928	0	27MAR15	613	N	N	N	N							YES
929	0	27MAR15	559	N	N	N	N							YES
93	0	06MAR15	517	N	N	N	N							YES
930	0	27MAR15	590	N	N	N	N							YES
931	0	27MAR15	510	N	N	N	N	29	1	106.5				NO
932	0	27MAR15	540	N	N	N	N	9	1	103.7	23	BRD		NO

DATA HAVE NOT BEEN VERIFIED

[1] Did not qualify for BRD re-treatment, not a BRD mortality and not removed for non-BRD reasons

PHASE IV ALL SPECIES STUDY BOVINE
STUDY: A131R-US-13-231
TREATMENT SUCCESS RATE
SUMMARY BY ANIMAL

treatment=T01 test material=DRAXXIN period=Arrival

animal	day of study	day 0 date	day 0 weight (LBS)	histophilus somni	mannheimia haemolytica	mycoplasma bovis	pasteurella multocida	day of BRD	score	temperature (F)	day of removal	brd related	BRD mortality	treatment success [1]
933	0	27MAR15	540	N	N	N	N							YES
934	0	27MAR15	518	N	N	N	N	11	1	106.7				NO
935	0	27MAR15	576	N	N	N	N							YES
936	0	27MAR15	482	N	N	N	N							YES
937	0	27MAR15	546	N	N	N	N							YES
938	0	27MAR15	506	N	N	N	N							YES
939	0	27MAR15	462	N	N	N	N							YES
94	0	06MAR15	452	N	N	N	Y							YES
940	0	27MAR15	507	N	N	N	N							YES
941	0	27MAR15	550	N	N	N	N							YES
942	0	28MAR15	449	N	N	N	N	11	1	105.7	13	BRD		NO
943	0	28MAR15	465	N	N	N	N							YES
944	0	28MAR15	440	N	N	N	N	11	1	104.5				NO
945	0	28MAR15	470	N	N	N	N	13	2	106.6				NO
946	0	28MAR15	539	N	N	N	N	39	1	103.5				NO
947	0	28MAR15	477	N	Y	N	N	11	1	104.7	13	BRD		NO
948	0	28MAR15	551	N	N	N	N							YES
949	0	28MAR15	433	N	N	N	N	11	1	105.1	20	BRD		NO
95	0	13MAR15	475	N	N	N	N							YES
950	0	28MAR15	454	N	N	N	N	12	2	107.7	19	BRD		NO
951	0	28MAR15	448	N	N	N	N	11	1	104.3	21	BRD		NO
952	0	28MAR15	462	N	Y	N	N							YES
953	0	28MAR15	488	N	N	N	N	7	1	106.6	18	BRD		NO
954	0	28MAR15	511	N	N	N	N							YES
955	0	28MAR15	472	N	N	N	N							YES
956	0	28MAR15	509	N	N	N	N	12	1	107.2	15	BRD		NO
957	0	28MAR15	447	N	N	N	N							YES
958	0	28MAR15	456	N	N	N	N	10	1	105.6	19	BRD		NO
959	0	28MAR15	465	N	N	N	N							YES
96	0	13MAR15	542	N	N	N	N							YES
960	0	28MAR15	511	N	N	N	N							YES
961	0	28MAR15	418	N	N	N	Y							YES
962	0	28MAR15	446	N	N	N	N	17	1	106.3				NO
963	0	28MAR15	445	N	N	N	N							YES

DATA HAVE NOT BEEN VERIFIED

[1] Did not qualify for BRD re-treatment, not a BRD mortality and not removed for non-BRD reasons

STUDY: A131R-US-13-231
TREATMENT SUCCESS RATE
SUMMARY BY ANIMAL

treatment=T01 test material=DRAXXIN period=Arrival

animal	day of study	day 0 date	day 0 weight (LBS)	histophilus somni	mannheimia haemolytica	mycoplasma bovis	pasteurella multocida	day of BRD	score	temperature (F)	day of removal	brd related	BRD mortality	treatment success [1]
964	0	28MAR15	438	N	N	N	N	31	1	104.0				NO
965	0	28MAR15	487	N	N	N	N							YES
966	0	28MAR15	448	N	N	N	N							YES
967	0	28MAR15	460	N	N	N	N							YES
968	0	28MAR15	425	N	N	N	N							YES
969	0	28MAR15	441	N	N	N	N							YES
97	0	13MAR15	515	N	N	N	N	7	1	103.5				NO
970	0	28MAR15	465	N	N	N	N	37	1	103.9				NO
971	0	28MAR15	480	N	N	N	N							YES
972	0	28MAR15	489	N	N	N	N	17	1	105.1	27	BRD		NO
973	0	28MAR15	417	N	N	N	N	14	1	105.6	21	BRD		NO
974	0	28MAR15	467	N	N	N	N							YES
975	0	28MAR15	507	N	N	N	N	9	1	103.7	16	BRD		NO
976	0	28MAR15	587	N	N	N	N							YES
977	0	28MAR15	430	N	N	N	N							YES
978	0	28MAR15	519	N	N	N	N							YES
979	0	28MAR15	429	N	Y	N	N							YES
98	0	13MAR15	533	N	N	N	N	10	1	104.4	17	BRD		NO
980	0	28MAR15	522	N	N	N	N							YES
981	0	28MAR15	424	N	N	N	N							YES
982	0	28MAR15	513	N	N	N	N	8	1	104.5	12	BRD		NO
983	0	28MAR15	475	N	N	N	N	16	1	105.1				NO
984	0	28MAR15	463	N	N	N	N							YES
985	0	28MAR15	520	N	N	N	N							YES
986	0	28MAR15	530	N	N	N	N							YES
987	0	28MAR15	431	N	N	N	N	21	1	105.1				NO
988	0	28MAR15	561	N	N	N	N							YES
989	0	28MAR15	382	N	N	N	N	8	1	107.2	13	BRD		NO
99	0	13MAR15	482	N	N	N	N							YES
990	0	28MAR15	516	N	Y	N	N	16	1	106.9	18	BRD		NO
991	0	28MAR15	464	N	N	N	N							YES
992	0	28MAR15	471	N	N	N	N							YES
993	0	28MAR15	593	N	N	N	Y	7	1	104.4				NO
994	0	28MAR15	454	N	N	N	N							YES

DATA HAVE NOT BEEN VERIFIED

[1] Did not qualify for BRD re-treatment, not a BRD mortality and not removed for non-BRD reasons

PHASE IV ALL SPECIES STUDY BOVINE
STUDY: A131R-US-13-231
TREATMENT SUCCESS RATE
SUMMARY BY ANIMAL

treatment=T01 test material=DRAXXIN period=Arrival

animal	day of study	day 0 date	day 0 weight (LBS)	histophilus somni	mannheimia haemolytica	mycoplasma bovis	pasteurella multocida	day of BRD	score	temperature (F)	day of removal	brd related	BRD mortality	treatment success [1]
995	0	28MAR15	536	N	N	N	N							YES
996	0	28MAR15	536	N	N	N	N	10	1	103.6				NO
997	0	28MAR15	494	N	N	N	N							YES
998	0	28MAR15	417	N	N	N	N							YES
999	0	28MAR15	484	N	N	N	N	12	1	106.3	19	BRD		NO

DATA HAVE NOT BEEN VERIFIED

[1] Did not qualify for BRD re-treatment, not a BRD mortality and not removed for non-BRD reasons

PHASE IV ALL SPECIES STUDY BOVINE
STUDY: A131R-US-13-231
TREATMENT SUCCESS RATE
SUMMARY BY ANIMAL

treatment=T01 test material=DRAXXIN period=First Pull

animal	day of study	day 0 date	day 0 weight (LBS)	histophilus somni	mannheimia haemolytica	mycoplasma bovis	pasteurella multocida	Bovine Parainfluenza 3 (qPCR-BPI3)	Bovine Respiratory Syncytial Virus (qPCR-BRSV-PCR)	Bovine Viral Diarrhea (qPCR-BVD-PCR)
100	9	13MAR15	508	N	N	N	N	Positive	Positive	Positive
1004	19	28MAR15	540	N	Y	P	N	Negative	Negative	Negative
1006	12	28MAR15	450	N	Y	N	N	Negative	Positive	Negative
1009	25	28MAR15	471	N	N	P	N	Positive	Negative	Negative
101	10	13MAR15	556	N	Y	N	N	Positive	Positive	Positive
1011	24	28MAR15	487	N	Y	P	N	Negative	Negative	Negative
1016	37	28MAR15	468	N	Y		Y	Negative	Positive	Negative
102	7	13MAR15	473	N	N	Y	N	Positive	Positive	Positive
1030	11	28MAR15	533	N	Y	N	N	Positive	Positive	Positive
1032	14	28MAR15	470	N	Y	N	N	Negative	Positive	Negative
1033	16	28MAR15	487	N	Y	N	N	Negative	Negative	Negative
1034	37	28MAR15	497	N	N		N	Negative	Positive	Negative
1035	11	28MAR15	456	N	N	N	N	Positive	Positive	Negative
1036	8	28MAR15	439	N	N	N	N	Negative	Negative	Positive
1037	11	28MAR15	437	N	N	N	N	Positive	Negative	Negative
104	12	13MAR15	548	N	Y	Y	N	Negative	Negative	Negative
105	7	13MAR15	523	N	N	N	N	Positive	Positive	Positive
108	8	13MAR15	453	N	N	Y	N	Positive	Positive	Negative
11	10	06MAR15	438	N	N	N	N	Negative	Positive	Negative
110	9	13MAR15	456	N	N	N	N	Positive	Positive	Positive
112	11	13MAR15	551	N	N	Y	Y	Positive	Negative	Positive
113	12	13MAR15	543	N	N	N	N	Positive	Negative	Positive
114	14	13MAR15	559	N	N	N	N	Negative	Positive	Negative
115	7	13MAR15	493	N	N	N	N	Positive	Positive	Positive
117	12	13MAR15	447	N	N	Y	N	Negative	Negative	Positive
118	7	13MAR15	570	N	N	N	N	Positive	Positive	Positive
12	7	06MAR15	447	N	N	N	N	Positive	Negative	Negative
120	15	13MAR15	507	N	N	N	N	Negative	Negative	Negative
124	28	13MAR15	536	N	N	P	N	Negative	Negative	Negative
127	12	13MAR15	440	N	N	Y	N	Positive	Positive	Positive
128	14	13MAR15	510	N	N	Y	N	Negative	Negative	Negative
130	11	13MAR15	590	N	Y	Y	N	Positive	Negative	Positive

DATA HAVE NOT BEEN VERIFIED

[1] Did not qualify for BRD re-treatment, not a BRD mortality and not removed for non-BRD reasons

treatment=T01 test material=DRAXXIN period=First Pull

animal	Infectious Bovine Rhinotracheitis (qPCR-IBR-BHV)	day of BRD	score	temperature (F)	day of removal	brd related	BRD mortality	treatment success [1]
100	Negative				13	BRD		NO
1004	Negative							YES
1006	Negative							YES
1009	Negative				28	BRD		NO
101	Positive	18	1	103.5	18	BRD		NO
1011	Negative							YES
1016	Negative	40	3	102.4	40	BRD		NO
102	Negative							YES
1030	Negative	19	1	105.5	19	BRD		NO
1032	Negative							YES
1033	Negative							YES
1034	Negative							YES
1035	Negative				18	BRD		NO
1036	Negative				14	BRD		NO
1037	Negative				14	BRD		NO
104	Negative							YES
105	Negative				15	BRD		NO
108	Negative				35	BRD		NO
11	Negative							YES
110	Positive				17	BRD		NO
112	Negative							YES
113	Negative				19	BRD		NO
114	Negative							YES
115	Negative				13	BRD		NO
117	Negative							YES
118	Negative							YES
12	Negative							YES
120	Negative							YES
124	Negative							YES
127	Positive				39	BRD		NO
128	Negative				23	BRD		NO
130	Negative	13	3	105.9	13	BRD		NO

DATA HAVE NOT BEEN VERIFIED

[1] Did not qualify for BRD re-treatment, not a BRD mortality and not removed for non-BRD reasons

PHASE IV ALL SPECIES STUDY BOVINE
STUDY: A131R-US-13-231
TREATMENT SUCCESS RATE
SUMMARY BY ANIMAL

treatment=T01 test material=DRAXXIN period=First Pull

animal	day of study	day 0 date	day 0 weight (LBS)	histophilus somni	mannheimia haemolytica	mycoplasma bovis	pasteurella multocida	Bovine Parainfluenza 3 (qPCR-BPI3)	Bovine Respiratory Syncytial Virus (qPCR-BRSV-PCR)	Bovine Viral Diarrhea (qPCR-BVD-PCR)
132	14	13MAR15	533	N	N	N	N	Positive	Negative	Positive
133	11	13MAR15	532	N	Y	Y	N	Positive	Positive	Positive
137	12	13MAR15	540	N	N	Y	N	Positive	Positive	Positive
139	12	13MAR15	498	N	N	N	N	Positive	Positive	Positive
140	12	13MAR15	476	N	N	Y	N	Positive	Negative	Negative
141	11	13MAR15	539	N	Y	Y	N	Positive	Negative	Positive
142	12	13MAR15	462	N	Y	Y	N	Negative	Negative	Positive
144	10	13MAR15	514	N	N	Y	N	Positive	Positive	Positive
146	13	13MAR15	467	N	N	N	N	Positive	Negative	Negative
147	17	13MAR15	502	N	N	Y	N	Negative	Negative	Negative
148	7	13MAR15	563	N	N	N	N	Positive	Positive	Negative
149	12	13MAR15	510	N	N	N	N	Negative	Negative	Positive
151	7	13MAR15	550	N	N	Y	N	Positive	Positive	Positive
156	8	13MAR15	431	N	N	N	N	Positive	Positive	Positive
159	11	13MAR15	541	Y	Y	Y	N	Negative	Positive	Positive
160	7	13MAR15	549	Y	N	Y	N	Positive	Positive	Positive
163	11	13MAR15	499	N	Y	Y	N	Positive	Positive	Positive
164	12	13MAR15	429	N	N	N	Y	Positive	Positive	Negative
165	8	13MAR15	501	N	N	N	N	Positive	Positive	Positive
166	8	13MAR15	568	N	N	N	N	Positive	Positive	Positive
167	18	13MAR15	569	N	Y	Y	N	Negative	Positive	Negative
169	13	13MAR15	548	N	N	Y	N	Negative	Negative	Positive
17	14	06MAR15	486	N	N	Y	N	Positive	Positive	Positive
171	10	13MAR15	524	N	N	Y	N	Positive	Positive	Negative
172	9	13MAR15	448	N	N	N	N	Positive	Positive	Positive
174	12	13MAR15	436	N	N	Y	N	Positive	Negative	Negative
175	8	13MAR15	546	N	N	N	N	Positive	Positive	Positive
177	8	13MAR15	507	N	N	Y	N	Positive	Positive	Positive
179	17	13MAR15	542	N	Y	Y	N	Negative	Negative	Negative
181	17	13MAR15	478	N	Y	Y	N	Negative	Negative	Negative
184	7	13MAR15	517	N	Y	Y	N	Positive	Positive	Negative
187	7	13MAR15	471	N	N	N	N	Positive	Positive	Positive

DATA HAVE NOT BEEN VERIFIED

[1] Did not qualify for BRD re-treatment, not a BRD mortality and not removed for non-BRD reasons

treatment=T01 test material=DRAXXIN period=First Pull

animal	Infectious Bovine Rhinotracheitis (qPCR-IBR-BHV)	day of BRD	score	temperature (F)	day of removal	brd related	BRD mortality	treatment success [1]
132	Negative				34	BRD		NO
133	Positive							YES
137	Negative				26	BRD		NO
139	Negative				17	BRD		NO
140	Negative				28	BRD		NO
141	Negative	40	1	106.0	40	BRD		NO
142	Negative							YES
144	Negative							YES
146	Negative							YES
147	Negative							YES
148	Negative				16	BRD		NO
149	Negative							YES
151	Negative							YES
156	Negative							YES
159	Positive	36	2	105.1	36	BRD		NO
160	Negative				14	BRD		NO
163	Negative	18	2	106.2	18	BRD		NO
164	Negative	19	2		20	BRD		NO
165	Positive				17	BRD		NO
166	Negative				16	BRD		NO
167	Negative							YES
169	Negative							YES
17	Negative							YES
171	Negative				16	BRD		NO
172	Negative				18	BRD		NO
174	Negative							YES
175	Positive				11	BRD		NO
177	Positive				30	BRD		NO
179	Negative	35	1	103.5	35	BRD		NO
181	Negative							YES
184	Negative	13	3	107.3	13	BRD		NO
187	Positive							YES

DATA HAVE NOT BEEN VERIFIED

[1] Did not qualify for BRD re-treatment, not a BRD mortality and not removed for non-BRD reasons

PHASE IV ALL SPECIES STUDY BOVINE
STUDY: A131R-US-13-231
TREATMENT SUCCESS RATE
SUMMARY BY ANIMAL

treatment=T01 test material=DRAXXIN period=First Pull

animal	day of study	day 0 date	day 0 weight (LBS)	histophilus somni	mannheimia haemolytica	mycoplasma bovis	pasteurella multocida	Bovine Parainfluenza 3 (qPCR-BPI3)	Bovine Respiratory Syncytial Virus (qPCR-BRSV-PCR)	Bovine Viral Diarrhea (qPCR-BVD-PCR)
188	34	13MAR15	540	N	N	N	N	Negative	Negative	Negative
191	13	13MAR15	436	N	N	N	N	Negative	Negative	Positive
192	7	13MAR15	474	N	N	N	N	Positive	Positive	Positive
195	14	13MAR15	546	N	N	N	N	Negative	Positive	Negative
20	11	06MAR15	438	N	N	Y	N	Negative	Positive	Positive
200	40	13MAR15	477	Y	Y	N	Y	Negative	Negative	Negative
201	7	13MAR15	470	N	Y	N	N	Positive	Positive	Negative
202	18	13MAR15	575	N	Y	Y	N	Positive	Positive	Negative
203	25	13MAR15	496	N	N	N	N	Negative	Positive	Negative
209	7	13MAR15	545	N	N	N	N	Positive	Positive	Negative
212	13	13MAR15	483	N	Y	Y	N	Negative	Negative	Negative
213	25	13MAR15	546	N	N	N	N	Negative	Negative	Negative
214	11	13MAR15	475	N	N	N	N	Negative	Positive	Positive
215	7	13MAR15	482	N	N	N	N	Positive	Positive	Negative
217	7	13MAR15	516	N	N	P	N	Positive	Positive	Negative
22	13	06MAR15	466	N	N	Y	N	Positive	Positive	Positive
221	19	13MAR15	486	N	Y	N	N	Positive	Negative	Negative
222	8	13MAR15	490	N	Y	Y	N	Positive	Positive	Negative
227	18	13MAR15	499	N	N	Y	N	Negative	Positive	Negative
232	14	13MAR15	508	N	Y	Y	N	Positive	Positive	Negative
234	20	13MAR15	482	N	N	N	N	Negative	Positive	Negative
238	7	13MAR15	446	N	N	N	N	Positive	Positive	Negative
24	7	06MAR15	562	N	N	N	N	Negative	Negative	Positive
247	8	13MAR15	534	N	N	N	N	Positive	Positive	Positive
248	17	13MAR15	532	N	Y	N	N	Negative	Positive	Negative
250	25	13MAR15	500	N	Y	P	Y	Negative	Negative	Negative
255	18	13MAR15	450	N	Y	N	N	Positive	Positive	Negative
256	29	13MAR15	540	N	N	N	N	Negative	Negative	Negative
258	18	13MAR15	480	N	N	Y	Y	Negative	Positive	Negative
26	7	06MAR15	502	N	N	N	N	Negative	Negative	Positive
262	12	13MAR15	500	N	N	N	N	Negative	Negative	Positive
264	21	13MAR15	533	Y	Y	P	N	Negative	Negative	Negative

DATA HAVE NOT BEEN VERIFIED

[1] Did not qualify for BRD re-treatment, not a BRD mortality and not removed for non-BRD reasons

PHASE IV ALL SPECIES STUDY BOVINE
STUDY: A131R-US-13-231
TREATMENT SUCCESS RATE
SUMMARY BY ANIMAL

treatment=T01 test material=DRAXXIN period=First Pull

animal	Infectious Bovine Rhinotracheitis (qPCR-IBR-BHV)	day of BRD	score	temperature (F)	day of removal	brd related	BRD mortality	treatment success [1]
188	Negative							YES
191	Negative				21	BRD		NO
192	Negative				10	BRD		NO
195	Negative							YES
20	Positive				24	BRD		NO
200	Negative							YES
201	Negative							YES
202	Negative							YES
203	Negative				30	BRD		NO
209	Negative				12	BRD		NO
212	Negative							YES
213	Negative							YES
214	Positive							YES
215	Negative				19	BRD		NO
217	Negative							YES
22	Negative				20	BRD		NO
221	Negative							YES
222	Negative							YES
227	Negative							YES
232	Negative	20	3	105.0	20	BRD		NO
234	Positive				29	BRD		NO
238	Negative				17	BRD		NO
24	Negative				12	BRD		NO
247	Positive				27	BRD		NO
248	Negative							YES
250	Negative							YES
255	Negative							YES
256	Negative							YES
258	Negative	28	1	103.7	28	BRD		NO
26	Positive							YES
262	Negative				19	BRD		NO
264	Negative	28	3	105.4	28	BRD		NO

DATA HAVE NOT BEEN VERIFIED

[1] Did not qualify for BRD re-treatment, not a BRD mortality and not removed for non-BRD reasons

PHASE IV ALL SPECIES STUDY BOVINE
STUDY: A131R-US-13-231
TREATMENT SUCCESS RATE
SUMMARY BY ANIMAL

treatment=T01 test material=DRAXXIN period=First Pull

animal	day of study	day 0 date	day 0 weight (LBS)	histophilus somni	mannheimia haemolytica	mycoplasma bovis	pasteurella multocida	Bovine Parainfluenza 3 (qPCR-BPI3)	Bovine Respiratory Syncytial Virus (qPCR-BRSV-PCR)	Bovine Viral Diarrhea (qPCR-BVD-PCR)
265	20	13MAR15	435	N	N	N	Y	Positive	Negative	Negative
266	21	13MAR15	552	N	Y	N	N	Negative	Negative	Negative
268	18	13MAR15	560	N	Y	Y	N	Negative	Negative	Negative
271	21	13MAR15	527	N	Y	P	N	Negative	Negative	Negative
272	33	13MAR15	463	Y	N	P	N	Negative	Negative	Negative
275	23	13MAR15	492	N	N	N	N	Negative	Negative	Negative
277	19	13MAR15	480	N	Y	N	N	Negative	Positive	Negative
278	21	13MAR15	564	N	N	N	N	Negative	Negative	Negative
280	17	13MAR15	591	N	N	N	N	Negative	Positive	Negative
287	17	13MAR15	577	N	N	N	N	Positive	Positive	Negative
288	21	13MAR15	453	N	N	P	N	Negative	Negative	Negative
291	13	20MAR15	480	N	Y	P	N	Positive	Positive	Positive
292	12	20MAR15	441	N	N	N	N	Negative	Negative	Negative
295	7	20MAR15	470	N	N	N	N	Positive	Negative	Negative
3	13	06MAR15	470	N	N	Y	N	Positive	Positive	Positive
30	7	06MAR15	524	N	N	N	N	Negative	Negative	Positive
300	19	20MAR15	455	N	N	N	N	Positive	Positive	Negative
301	7	20MAR15	510	N	N	N	N	Negative	Positive	Negative
302	7	20MAR15	487	N	N	N	N	Negative	Negative	Negative
309	7	20MAR15	444	N	N	N	N	Negative	Positive	Negative
311	12	20MAR15	405	N	Y	Y	N	Negative	Positive	Negative
312	18	20MAR15	484	N	Y	N	N	Negative	Negative	Negative
315	25	20MAR15	425	N	N	P	Y	Negative	Negative	Negative
318	25	20MAR15	455	N	Y	P	Y	Negative	Negative	Negative
319	11	20MAR15	439	N	N	N	N	Positive	Positive	Negative
320	14	20MAR15	459	N	N	N	N	Positive	Positive	Negative
323	14	20MAR15	430	N	Y	P	N	Negative	Positive	Negative
327	7	20MAR15	502	N	N	N	N	Negative	Positive	Negative
329	15	20MAR15	465	N	Y	N	N	Negative	Positive	Negative
33	12	06MAR15	510	N	N	N	N	Negative	Positive	Positive
330	8	20MAR15	454	N	N	N	N	Negative	Positive	Negative
332	9	20MAR15	450	N	N	N	N	Negative	Positive	Negative

DATA HAVE NOT BEEN VERIFIED

[1] Did not qualify for BRD re-treatment, not a BRD mortality and not removed for non-BRD reasons

PHASE IV ALL SPECIES STUDY BOVINE
STUDY: A131R-US-13-231
TREATMENT SUCCESS RATE
SUMMARY BY ANIMAL

treatment=T01 test material=DRAXXIN period=First Pull

animal	Infectious Bovine Rhinotracheitis (qPCR-IBR-BHV)	day of BRD	score	temperature (F)	day of removal	brd related	BRD mortality	treatment success [1]
265	Negative	30	1	104.0	30	BRD		NO
266	Positive							YES
268	Negative							YES
271	Negative	28	2	105.1	28	BRD		NO
272	Negative							YES
275	Negative				25	BRD		NO
277	Negative							YES
278	Negative							YES
280	Negative				24	BRD		NO
287	Negative							YES
288	Negative							YES
291	Positive	20	2	104.2	20	BRD		NO
292	Negative				24	BRD		NO
295	Negative				22	BRD		NO
3	Negative							YES
30	Positive				12	BRD		NO
300	Negative				35	BRD		NO
301	Negative				15	BRD		NO
302	Negative							YES
309	Negative							YES
311	Negative	19	2	105.7	19	BRD		NO
312	Negative							YES
315	Negative							YES
318	Negative	32	1	103.8	32	BRD		NO
319	Negative				18	BRD		NO
320	Negative							YES
323	Negative							YES
327	Negative							YES
329	Negative							YES
33	Positive				21	BRD		NO
330	Negative				12	BRD		NO
332	Negative							YES

DATA HAVE NOT BEEN VERIFIED

[1] Did not qualify for BRD re-treatment, not a BRD mortality and not removed for non-BRD reasons

PHASE IV ALL SPECIES STUDY BOVINE
STUDY: A131R-US-13-231
TREATMENT SUCCESS RATE
SUMMARY BY ANIMAL

treatment=T01 test material=DRAXXIN period=First Pull

animal	day of study	day 0 date	day 0 weight (LBS)	histophilus somni	mannheimia haemolytica	mycoplasma bovis	pasteurella multocida	Bovine Parainfluenza 3 (qPCR-BPI3)	Bovine Respiratory Syncytial Virus (qPCR-BRSV-PCR)	Bovine Viral Diarrhea (qPCR-BVD-PCR)
34	8	06MAR15	529	N	N	Y	N			
341	10	20MAR15	471	N	N	N	N	Negative	Positive	Positive
342	7	20MAR15	459	N	N	N	N	Negative	Negative	Negative
343	18	20MAR15	557	N	Y	N	N	Negative	Positive	Negative
346	13	20MAR15	422	N	N	P	Y	Negative	Negative	Negative
349	16	20MAR15	441	N	Y	P	N	Positive	Positive	Negative
35	16	06MAR15	495	N	N	N	N	Positive	Negative	Negative
350	21	20MAR15	471	N	N	N	N	Negative	Negative	Negative
353	19	20MAR15	457	N	N	N	N	Positive	Positive	Negative
356	7	20MAR15	404	N	N	N	N	Negative	Negative	Negative
357	11	20MAR15	429	N	N	N	N	Positive	Positive	Positive
365	19	20MAR15	457	N	Y	N	N	Negative	Positive	Negative
366	10	20MAR15	434	N	N	N	N	Negative	Positive	Positive
367	14	20MAR15	474	N	Y	N	N	Negative	Positive	Negative
369	15	20MAR15	507	N	N	N	N	Negative	Positive	Negative
37	10	06MAR15	564	N	N	Y	N	Negative	Negative	Positive
370	12	20MAR15	452	N	N	N	N	Negative	Negative	Negative
373	20	20MAR15	449	N	N	N	N	Negative	Negative	Negative
374	13	20MAR15	475	N	Y	P	N	Positive	Negative	Negative
377	23	20MAR15	436	N	Y	P	N	Negative	Negative	Negative
378	10	20MAR15	495	N	N	Y	N	Negative	Negative	Positive
379	10	20MAR15	475	N	N	Y	N	Negative	Negative	Negative
38	11	06MAR15	464	N	N	N	N	Negative	Negative	Positive
380	15	20MAR15	411	N	N	P	N	Negative	Negative	Negative
385	9	20MAR15	484	N	N	N	N	Negative	Positive	Negative
386	12	20MAR15	546	N	N	N	N	Negative	Negative	Negative
388	15	20MAR15	471	N	Y	P	N	Negative	Negative	Negative
39	12	06MAR15	542	N	N	Y	N	Negative	Positive	Positive
390	16	20MAR15	573	N	Y	P	N	Negative	Positive	Negative
391	14	20MAR15	597	N	Y	P	N	Negative	Positive	Negative
395	7	20MAR15	614	N	N	N	N	Negative	Positive	Negative
396	19	20MAR15	536	N	N	P	N	Positive	Positive	Negative

DATA HAVE NOT BEEN VERIFIED

[1] Did not qualify for BRD re-treatment, not a BRD mortality and not removed for non-BRD reasons

PHASE IV ALL SPECIES STUDY BOVINE
STUDY: A131R-US-13-231
TREATMENT SUCCESS RATE
SUMMARY BY ANIMAL

treatment=T01 test material=DRAXXIN period=First Pull

animal	Infectious Bovine Rhinotracheitis (qPCR-IBR-BHV)	day of BRD	score	temperature (F)	day of removal	brd related	BRD mortality	treatment success [1]
34					15	BRD		NO
341	Negative				16	BRD		NO
342	Negative							YES
343	Negative	26	1	104.5	26	BRD		NO
346	Positive	23	3	105.8	23	BRD		NO
349	Negative	27	1	104.8	27	BRD		NO
35	Negative							YES
350	Negative							YES
353	Negative							YES
356	Negative				18	BRD		NO
357	Negative				15	BRD	YES	NO
365	Negative	27	1	105.3	27	BRD		NO
366	Negative				17	BRD		NO
367	Negative	17	3	105.6	17	BRD		NO
369	Negative							YES
37	Negative							YES
370	Negative				16	BRD		NO
373	Negative							YES
374	Negative	15	3	104.5	15	BRD	YES	NO
377	Negative							YES
378	Negative				17	BRD		NO
379	Negative				16	BRD		NO
38	Negative							YES
380	Negative				18	BRD		NO
385	Negative							YES
386	Negative							YES
388	Negative	19	3	104.5	19	BRD		NO
39	Positive				29	BRD		NO
390	Negative							YES
391	Negative	21	2	106.0	21	BRD		NO
395	Negative							YES
396	Negative							YES

DATA HAVE NOT BEEN VERIFIED

[1] Did not qualify for BRD re-treatment, not a BRD mortality and not removed for non-BRD reasons

PHASE IV ALL SPECIES STUDY BOVINE
STUDY: A131R-US-13-231
TREATMENT SUCCESS RATE
SUMMARY BY ANIMAL

treatment=T01 test material=DRAXXIN period=First Pull

animal	day of study	day 0 date	day 0 weight (LBS)	histophilus somni	mannheimia haemolytica	mycoplasma bovis	pasteurella multocida	Bovine Parainfluenza 3 (qPCR-BPI3)	Bovine Respiratory Syncytial Virus (qPCR-BRSV-PCR)	Bovine Viral Diarrhea (qPCR-BVD-PCR)
406	17	20MAR15	533	N	Y	P	N	Positive	Negative	Negative
407	11	20MAR15	537	N	N	N	N	Negative	Positive	Negative
410	10	20MAR15	505	N	N	N	N	Negative	Positive	Negative
411	12	20MAR15	538	N	Y	Y	N	Negative	Positive	Negative
415	10	20MAR15	521	N	Y	N	N	Positive	Positive	Negative
416	13	20MAR15	489	N	Y	P	N	Negative	Positive	Negative
417	7	20MAR15	515	N	N	N	N	Negative	Positive	Positive
422	15	20MAR15	514	N	Y	P	N	Negative	Positive	Negative
428	14	20MAR15	515	N	N	P	Y	Negative	Positive	Negative
431	19	20MAR15	537	N	N	N	N	Negative	Positive	Negative
433	12	20MAR15	546	N	N	Y	Y	Negative	Positive	Negative
436	19	20MAR15	537	N	N	N	N	Negative	Positive	Negative
437	11	20MAR15	544	N	N	N	N	Negative	Positive	Negative
44	10	06MAR15	450	N	N	Y	N	Negative	Negative	Positive
440	7	20MAR15	508	N	N	N	N	Negative	Positive	Negative
441	19	20MAR15	506	N	N	N	N	Negative	Positive	Negative
445	12	20MAR15	551	N	Y	N	N	Positive	Positive	Negative
447	12	20MAR15	515	N	N	N	N	Negative	Positive	Negative
448	10	20MAR15	533	N	N	N	N	Negative	Positive	Negative
45	7	06MAR15	430	N	N	N	N	Negative	Negative	Negative
450	11	20MAR15	541	N	N	N	N	Negative	Positive	Negative
452	23	20MAR15	530	Y	N	N	Y	Negative	Negative	Negative
453	7	20MAR15	538	N	N	N	N	Negative	Positive	Negative
454	11	20MAR15	523	Y	Y	Y	N	Negative	Positive	Positive
456	10	20MAR15	548	N	N	N	N	Negative	Positive	Positive
458	12	20MAR15	552	N	N	Y	N	Negative	Positive	Negative
46	7	06MAR15	470	N	N	N	N	Negative	Negative	Negative
460	24	20MAR15	508	N	N	N	N	Negative	Negative	Negative
461	9	20MAR15	543	N	N	N	N	Negative	Negative	Negative
462	15	20MAR15	524					Negative	Positive	Positive
463	15	20MAR15	587	N	N	N	N	Negative	Negative	Negative
467	21	20MAR15	560	N	N	P	Y	Negative	Negative	Negative

DATA HAVE NOT BEEN VERIFIED

[1] Did not qualify for BRD re-treatment, not a BRD mortality and not removed for non-BRD reasons

PHASE IV ALL SPECIES STUDY BOVINE
STUDY: A131R-US-13-231
TREATMENT SUCCESS RATE
SUMMARY BY ANIMAL

treatment=T01 test material=DRAXXIN period=First Pull

animal	Infectious Bovine Rhinotracheitis (qPCR-IBR-BHV)	day of BRD	score	temperature (F)	day of removal	brd related	BRD mortality	treatment success [1]
406	Negative							YES
407	Negative							YES
410	Negative				17	BRD		NO
411	Negative							YES
415	Negative	17	1	104.8	17	BRD		NO
416	Positive							YES
417	Negative				15	BRD		NO
422	Positive	18	3	104.5	18	BRD		NO
428	Negative							YES
431	Negative							YES
433	Positive	15	3	104.4	15	BRD		NO
436	Negative							YES
437	Negative				30	BRD		NO
44	Negative				19	BRD		NO
440	Negative				20	BRD		NO
441	Negative							YES
445	Negative							YES
447	Negative				20	BRD		NO
448	Negative				15	BRD		NO
45	Negative				20	BRD		NO
450	Positive				18	BRD		NO
452	Negative	25	3	106.2	25	BRD		NO
453	Negative							YES
454	Positive	18	1	103.8	18	BRD		NO
456	Negative				15	BRD		NO
458	Negative				20	BRD		NO
46	Negative							YES
460	Negative							YES
461	Negative							YES
462	Negative							YES
463	Negative							YES
467	Negative							YES

DATA HAVE NOT BEEN VERIFIED

[1] Did not qualify for BRD re-treatment, not a BRD mortality and not removed for non-BRD reasons

PHASE IV ALL SPECIES STUDY BOVINE
STUDY: A131R-US-13-231
TREATMENT SUCCESS RATE
SUMMARY BY ANIMAL

treatment=T01 test material=DRAXXIN period=First Pull

animal	day of study	day 0 date	day 0 weight (LBS)	histophilus somni	mannheimia haemolytica	mycoplasma bovis	pasteurella multocida	Bovine Parainfluenza 3 (qPCR-BPI3)	Bovine Respiratory Syncytial Virus (qPCR-BRSV-PCR)	Bovine Viral Diarrhea (qPCR-BVD-PCR)
469	8	23MAR15	432	N	N	Y	N	Positive	Positive	Negative
470	15	23MAR15	497	N	N	N	N	Negative	Negative	Negative
471	16	23MAR15	529	N	Y	N	N	Positive	Positive	Negative
473	11	23MAR15	450	N	N	N	N	Negative	Negative	Negative
474	12	23MAR15	502	N	N	N	N	Negative	Negative	Negative
476	10	23MAR15	532	N	N	P	N	Positive	Positive	Positive
477	8	23MAR15	445	N	N	N	N	Negative	Positive	Positive
478	11	23MAR15	555	N	Y	P	N	Positive	Negative	Negative
484	14	23MAR15	537	N	Y	N	N	Positive	Positive	Negative
485	7	23MAR15	504	N	N	N	N	Positive	Positive	Negative
486	15	23MAR15	484	N	Y	N	Y	Negative	Positive	Negative
487	36	23MAR15	579	N	N	P	Y	Negative	Negative	Negative
488	10	23MAR15	430	N	N	P	N	Positive	Negative	Negative
489	7	23MAR15	503	N	N	P	N	Positive	Positive	Negative
49	21	06MAR15	484	Y	N	Y	N	Negative	Negative	Negative
491	7	23MAR15	482	N	Y	N	N	Positive	Negative	Negative
492	10	23MAR15	537	N	Y	P	N	Positive	Negative	Negative
493	9	23MAR15	505	N	N	N	N	Negative	Positive	Negative
494	7	23MAR15	428	N	N	N	N	Negative	Positive	Negative
496	12	23MAR15	542					Negative	Negative	Negative
497	9	23MAR15	439	N	N	N	N	Negative	Positive	Negative
499	7	23MAR15	420	N	N	N	N	Positive	Positive	Negative
5	10	06MAR15	472	N	N	N	N	Negative	Negative	Negative
501	12	23MAR15	551	N	N	N	N	Negative	Negative	Negative
503	24	23MAR15	512	Y	N	P	N	Negative	Negative	Negative
505	7	23MAR15	486	N	Y	Y	N	Negative	Negative	Negative
506	15	23MAR15	539	N	Y	P	N	Positive	Positive	Negative
508	15	23MAR15	485	N	N	P	N	Negative	Positive	Negative
509	8	23MAR15	457	N	Y	Y	N	Positive	Positive	Negative
51	10	06MAR15	514	N	Y	Y	N	Negative	Negative	Negative
510	16	23MAR15	575	Y	Y	P	N	Positive	Positive	Negative
511	11	23MAR15	485	N	N	N	N	Positive	Positive	Positive

DATA HAVE NOT BEEN VERIFIED

[1] Did not qualify for BRD re-treatment, not a BRD mortality and not removed for non-BRD reasons

PHASE IV ALL SPECIES STUDY BOVINE
STUDY: A131R-US-13-231
TREATMENT SUCCESS RATE
SUMMARY BY ANIMAL

treatment=T01 test material=DRAXXIN period=First Pull

animal	Infectious Bovine Rhinotracheitis (qPCR-IBR-BHV)	day of BRD	score	temperature (F)	day of removal	brd related	BRD mortality	treatment success [1]
469	Negative				17	BRD		NO
470	Negative				18	BRD		NO
471	Negative	18	3	104.1	18	BRD		NO
473	Negative				18	BRD		NO
474	Negative							YES
476	Positive				15	BRD		NO
477	Negative				18	BRD		NO
478	Negative	32	2	103.8	32	BRD		NO
484	Negative							YES
485	Negative				16	BRD		NO
486	Negative	18	3	107.4	18	BRD		NO
487	Negative	40	3	105.1	40	BRD		NO
488	Negative				22	BRD		NO
489	Negative				15	BRD		NO
49	Negative							YES
491	Negative	22	1	105.0	22	BRD		NO
492	Positive	14	3	104.1	14	BRD		NO
493	Negative				17	BRD		NO
494	Negative				19	BRD		NO
496	Negative							YES
497	Negative				20	BRD		NO
499	Negative				16	BRD		NO
5	Negative				20	BRD		NO
501	Negative							YES
503	Negative							YES
505	Negative	15	1	103.9	15	BRD		NO
506	Negative							YES
508	Negative				18	BRD		NO
509	Negative	21	2	102.5	21	BRD		NO
51	Negative	17	2	104.6	17	BRD		NO
510	Negative	24	1	104.0	24	BRD		NO
511	Negative				16	BRD		NO

DATA HAVE NOT BEEN VERIFIED

[1] Did not qualify for BRD re-treatment, not a BRD mortality and not removed for non-BRD reasons

PHASE IV ALL SPECIES STUDY BOVINE
STUDY: A131R-US-13-231
TREATMENT SUCCESS RATE
SUMMARY BY ANIMAL

treatment=T01 test material=DRAXXIN period=First Pull

animal	day of study	day 0 date	day 0 weight (LBS)	histophilus somni	mannheimia haemolytica	mycoplasma bovis	pasteurella multocida	Bovine Parainfluenza 3 (qPCR-BPI3)	Bovine Respiratory Syncytial Virus (qPCR-BRSV-PCR)	Bovine Viral Diarrhea (qPCR-BVD-PCR)
512	10	23MAR15	573	N	N	P	Y	Negative	Negative	Positive
513	21	23MAR15	527	Y	N	N	N	Negative	Negative	Negative
517	8	23MAR15	480	N	N	N	N	Negative	Positive	Negative
518	10	23MAR15	518	N	Y	N	N	Negative	Positive	Negative
519	15	23MAR15	506	N	N	N	N	Positive	Positive	Negative
52	12	06MAR15	509	N	N	N	N	Negative	Negative	Positive
520	7	23MAR15	545	N	N	Y	Y	Negative	Positive	Negative
522	9	23MAR15	478	N	N	N	N	Negative	Negative	Negative
524	15	23MAR15	500	N	Y	P	N	Positive	Negative	Negative
526	12	23MAR15	568	N	N	N	N	Negative	Positive	Negative
531	15	23MAR15	490	N	Y	P	N	Negative	Negative	Negative
532	13	23MAR15	496	N	N	N	N	Negative	Positive	Negative
537	17	23MAR15	491	N	Y	P	N	Negative	Positive	Negative
541	12	23MAR15	502	N	Y	N	N	Negative	Negative	Negative
542	19	23MAR15	473	N	Y	P	Y	Negative	Negative	Negative
543	11	23MAR15	510	N	N	N	N	Negative	Negative	Negative
547	36	23MAR15	433	N	Y	N	N	Negative	Negative	Negative
548	11	23MAR15	480	N	N	N	N	Negative	Positive	Negative
549	14	23MAR15	469	Y	N	P	Y	Negative	Negative	Negative
55	10	06MAR15	470	N	N	N	N	Negative	Negative	Positive
550	26	23MAR15	542	N	N	P	N	Negative	Negative	Negative
551	36	23MAR15	490	N	N	P	N	Negative	Negative	Negative
552	8	23MAR15	469	N	N	N	N	Negative	Positive	Negative
556	7	23MAR15	577	N	N	N	N	Negative	Negative	Negative
561	20	23MAR15	569	Y	N	N	N	Negative	Negative	Negative
564	11	23MAR15	481	N	N	N	Y	Negative	Positive	Negative
57	9	06MAR15	496	N	N	Y	N			
570	10	23MAR15	633	N	N	N	N	Positive	Positive	Negative
571	16	23MAR15	501	Y	N	N	N	Positive	Positive	Negative
576	7	23MAR15	563	N	N	N	N	Negative	Negative	Negative
582	29	23MAR15	554	N	N	P	N	Negative	Negative	Negative
583	8	23MAR15	566	N	N	Y	N	Negative	Positive	Negative

DATA HAVE NOT BEEN VERIFIED

[1] Did not qualify for BRD re-treatment, not a BRD mortality and not removed for non-BRD reasons

PHASE IV ALL SPECIES STUDY BOVINE
STUDY: A131R-US-13-231
TREATMENT SUCCESS RATE
SUMMARY BY ANIMAL

treatment=T01 test material=DRAXXIN period=First Pull

animal	Infectious Bovine Rhinotracheitis (qPCR-IBR-BHV)	day of BRD	score	temperature (F)	day of removal	brd related	BRD mortality	treatment success [1]
512	Positive							YES
513	Negative							YES
517	Negative				15	BRD		NO
518	Negative							YES
519	Negative							YES
52	Positive				19	BRD		NO
520	Negative	15	1	104.6	15	BRD		NO
522	Negative				27	BRD		NO
524	Negative	22	1	105.0	22	BRD		NO
526	Negative							YES
531	Negative	22	1	105.8	22	BRD		NO
532	Negative							YES
537	Negative	22	3	106.7	22	BRD		NO
541	Negative	19	1	104.6	19	BRD		NO
542	Positive							YES
543	Negative				19	BRD		NO
547	Negative							YES
548	Positive				19	BRD		NO
549	Negative	29	1	104.5	29	BRD		NO
55	Negative							YES
550	Negative							YES
551	Negative							YES
552	Negative				15	BRD		NO
556	Negative							YES
561	Negative							YES
564	Negative							YES
57					15	BRD		NO
570	Negative							YES
571	Negative							YES
576	Negative							YES
582	Negative							YES
583	Negative				20	BRD		NO

DATA HAVE NOT BEEN VERIFIED

[1] Did not qualify for BRD re-treatment, not a BRD mortality and not removed for non-BRD reasons

PHASE IV ALL SPECIES STUDY BOVINE
STUDY: A131R-US-13-231
TREATMENT SUCCESS RATE
SUMMARY BY ANIMAL

treatment=T01 test material=DRAXXIN period=First Pull

animal	day of study	day 0 date	day 0 weight (LBS)	histophilus somni	mannheimia haemolytica	mycoplasma bovis	pasteurella multocida	Bovine Parainfluenza 3 (qPCR-BPI3)	Bovine Respiratory Syncytial Virus (qPCR-BRSV-PCR)	Bovine Viral Diarrhea (qPCR-BVD-PCR)
585	16	23MAR15	540	N	N	N	N	Positive	Positive	Negative
595	16	23MAR15	619	N	N	N	N	Positive	Positive	Negative
601	8	23MAR15	554	N	N	Y	N	Negative	Positive	Negative
603	7	23MAR15	515	N	N	N	N	Negative	Negative	Negative
615	7	23MAR15	510	N	N	N	N	Negative	Negative	Negative
617	28	23MAR15	600	N	N	N	N	Negative	Negative	Negative
618	7	23MAR15	516	N	Y	Y	N	Negative	Positive	Positive
619	16	23MAR15	588	N	N	N	N	Positive	Positive	Negative
62	24	06MAR15	444	N	Y	Y	N	Negative	Negative	Negative
622	13	23MAR15	570	N	N	N	N	Negative	Positive	Negative
625	11	23MAR15	497	N	Y	N	N	Negative	Positive	Negative
626	16	23MAR15	490	N	N	N	N	Positive	Positive	Negative
630	24	23MAR15	550	N	N	P	N	Negative	Negative	Negative
631	7	23MAR15	568	N	N	N	N	Negative	Positive	Positive
636	8	23MAR15	573	N	N	N	N	Positive	Positive	Negative
64	10	06MAR15	484	N	N	N	N	Negative	Negative	Negative
644	15	25MAR15	402	N	Y	N	N	Negative	Negative	Negative
645	14	25MAR15	429	N	N	P	Y	Negative	Positive	Negative
65	11	06MAR15	503	N	N	Y	N	Negative	Negative	Positive
651	16	25MAR15	410	N	N	N	N	Negative	Negative	Negative
653	13	25MAR15	372	N	N	N	N	Negative	Positive	Negative
654	14	25MAR15	399	N	N	N	N	Negative	Positive	Negative
656	16	25MAR15	445	N	N	N	N	Negative	Negative	Negative
66	7	06MAR15	545	Y	N	N	N	Negative	Negative	Positive
665	20	25MAR15	437	N	Y	P	N	Negative	Negative	Negative
675	16	25MAR15	455	N	Y	N	N	Negative	Positive	Negative
680	16	25MAR15	378	N	N	N	N	Negative	Negative	Negative
69	10	06MAR15	497	N	N	N	N	Negative	Negative	Positive
692	17	25MAR15	413	N	N	N	N	Negative	Negative	Negative
693	15	25MAR15	433	N	Y	P	Y	Negative	Negative	Negative
695	13	25MAR15	428	N	N	N	N	Negative	Positive	Negative
7	7	06MAR15	481	N	N	N	N	Positive	Negative	Negative

DATA HAVE NOT BEEN VERIFIED

[1] Did not qualify for BRD re-treatment, not a BRD mortality and not removed for non-BRD reasons

treatment=T01 test material=DRAXXIN period=First Pull

animal	Infectious Bovine Rhinotracheitis (qPCR-IBR-BHV)	day of BRD	score	temperature (F)	day of removal	brd related	BRD mortality	treatment success [1]
585	Negative				27	BRD		NO
595	Negative							YES
601	Negative				31	BRD		NO
603	Negative				19	BRD		NO
615	Negative				16	BRD		NO
617	Negative							YES
618	Negative							YES
619	Negative				24	BRD		NO
62	Negative							YES
622	Negative							YES
625	Negative							YES
626	Negative							YES
630	Negative							YES
631	Negative				14	BRD		NO
636	Negative							YES
64	Negative							YES
644	Negative	22	1	103.7	22	BRD		NO
645	Negative							YES
65	Positive				17	BRD		NO
651	Negative							YES
653	Negative				20	BRD		NO
654	Negative				16	BRD		NO
656	Negative				35	BRD		NO
66	Negative				9	BRD		NO
665	Negative							YES
675	Negative							YES
680	Negative							YES
69	Negative				18	BRD		NO
692	Negative							YES
693	Negative	22	2	103.7	22	BRD		NO
695	Negative							YES
7	Negative							YES

DATA HAVE NOT BEEN VERIFIED

[1] Did not qualify for BRD re-treatment, not a BRD mortality and not removed for non-BRD reasons

STUDY: A131R-US-13-231
TREATMENT SUCCESS RATE
SUMMARY BY ANIMAL

treatment=T01 test material=DRAXXIN period=First Pull

animal	day of study	day 0 date	day 0 weight (LBS)	histophilus somni	mannheimia haemolytica	mycoplasma bovis	pasteurella multocida	Bovine Parainfluenza 3 (qPCR-BPI3)	Bovine Respiratory Syncytial Virus (qPCR-BRSV-PCR)	Bovine Viral Diarrhea (qPCR-BVD-PCR)
70	10	06MAR15	540	N	N	N	N	Positive	Negative	Negative
705	22	25MAR15	400	N	N	N	N	Negative	Negative	Negative
706	15	25MAR15	424	N	Y	N	N	Negative	Negative	Negative
708	16	25MAR15	407	N	Y	N	N	Negative	Positive	Negative
712	22	25MAR15	355	N	Y	N	N	Negative	Negative	Negative
713	15	25MAR15	409	N	Y	N	N	Negative	Positive	Negative
72	11	06MAR15	484	N	N	N	N	Positive	Positive	Positive
721	15	25MAR15	412	N	Y	N	N	Negative	Negative	Negative
724	35	25MAR15	380	Y	N	N	N	Negative	Negative	Negative
728	12	25MAR15	394	Y	N	P	N	Negative	Positive	Negative
73	12	06MAR15	508	N	N	N	N	Negative	Negative	Positive
737	14	25MAR15	340	N	Y	N	N	Positive	Positive	Negative
74	7	06MAR15	420	N	N	N	N	Positive	Negative	Positive
745	14	25MAR15	450	N	Y	N	N	Positive	Positive	Negative
748	14	25MAR15	434	N	N	N	N	Positive	Positive	Negative
752	14	25MAR15	434	N	Y	P	N	Positive	Positive	Negative
753	9	25MAR15	404	N	Y	N	N	Negative	Negative	Negative
754	18	25MAR15	349	N	Y	P	N	Negative	Negative	Negative
760	13	26MAR15	511	N	N	N	N	Positive	Positive	Negative
765	13	26MAR15	504	N	N	P	N	Positive	Positive	Negative
766	18	26MAR15	485	N	Y	N	N	Positive	Negative	Negative
769	30	26MAR15	475	N	N	P	N	Negative	Negative	Positive
771	10	26MAR15	492	N	N	N	N	Negative	Positive	Negative
772	18	26MAR15	558	Y	Y	P	N	Positive	Negative	Negative
773	10	26MAR15	454	N	Y	N	N	Negative	Negative	Positive
775	8	26MAR15	466	N	Y	N	N	Negative	Positive	Positive
779	13	26MAR15	467	N	N	N	N	Positive	Positive	Negative
783	14	26MAR15	505	N	Y	P	N	Negative	Positive	Negative
786	17	26MAR15	499	N	Y	P	N	Negative	Negative	Negative
792	10	26MAR15	507	N	N	N	N	Negative	Positive	Positive
793	9	26MAR15	532	N	Y	N	N	Negative	Positive	Positive
795	16	26MAR15	535	N	Y	N	N	Negative	Negative	Positive

DATA HAVE NOT BEEN VERIFIED

[1] Did not qualify for BRD re-treatment, not a BRD mortality and not removed for non-BRD reasons

PHASE IV ALL SPECIES STUDY BOVINE
STUDY: A131R-US-13-231
TREATMENT SUCCESS RATE
SUMMARY BY ANIMAL

treatment=T01 test material=DRAXXIN period=First Pull

animal	Infectious Bovine Rhinotracheitis (qPCR-IBR-BHV)	day of BRD	score	temperature (F)	day of removal	brd related	BRD mortality	treatment success [1]
70	Negative							YES
705	Negative							YES
706	Negative	22	3	106.8	22	BRD		NO
708	Negative							YES
712	Negative							YES
713	Negative	22	1	105.4	22	BRD		NO
72	Negative				17	BRD		NO
721	Negative	22	2	105.0	22	BRD		NO
724	Negative							YES
728	Positive				21	BRD		NO
73	Positive				19	BRD		NO
737	Negative							YES
74	Negative							YES
745	Negative							YES
748	Negative							YES
752	Negative	24	1	103.7	24	BRD		NO
753	Negative							YES
754	Negative							YES
760	Negative				34	BRD		NO
765	Negative							YES
766	Negative							YES
769	Negative							YES
771	Negative							YES
772	Negative	25	2	103.8	25	BRD		NO
773	Positive							YES
775	Negative	12	3	106.0	12	BRD		NO
779	Negative				20	BRD		NO
783	Negative	21	1	106.0	21	BRD		NO
786	Negative	33	2	103.2	33	BRD		NO
792	Negative				15	BRD		NO
793	Negative							YES
795	Negative							YES

DATA HAVE NOT BEEN VERIFIED

[1] Did not qualify for BRD re-treatment, not a BRD mortality and not removed for non-BRD reasons

PHASE IV ALL SPECIES STUDY BOVINE
STUDY: A131R-US-13-231
TREATMENT SUCCESS RATE
SUMMARY BY ANIMAL

treatment=T01 test material=DRAXXIN period=First Pull

animal	day of study	day 0 date	day 0 weight (LBS)	histophilus somni	mannheimia haemolytica	mycoplasma bovis	pasteurella multocida	Bovine Parainfluenza 3 (qPCR-BPI3)	Bovine Respiratory Syncytial Virus (qPCR-BRSV-PCR)	Bovine Viral Diarrhea (qPCR-BVD-PCR)
798	8	26MAR15	545	N	Y	N	Y	Negative	Positive	Negative
799	11	26MAR15	500	N	N	P	N	Positive	Positive	Positive
80	7	06MAR15	497	N	N	N	N	Negative	Negative	Positive
800	12	26MAR15	510	N	Y	N	N	Positive	Negative	Negative
805	9	26MAR15	474					Negative	Positive	Negative
806	11	26MAR15	482	N	N	N	N	Negative	Negative	Negative
807	7	26MAR15	520	N	N	N	N	Positive	Positive	Negative
809	13	26MAR15	513	Y	N	N	N	Negative	Negative	Negative
814	26	26MAR15	500	Y	N	P	N	Negative	Negative	Negative
815	7	26MAR15	515	N	N	N	N	Negative	Positive	Negative
818	8	26MAR15	450	Y	N	N	N	Negative	Negative	Positive
819	8	26MAR15	423	N	N	N	N	Negative	Negative	Negative
824	8	26MAR15	474	N	N	N	N	Negative	Positive	Negative
829	13	26MAR15	493	N	N	N	N	Positive	Positive	Positive
83	11	06MAR15	540	N	N	Y	N	Negative	Negative	Positive
832	13	26MAR15	446	N	N	N	N	Positive	Positive	Positive
834	7	26MAR15	520	N	N	N	N	Positive	Negative	Positive
836	19	26MAR15	443	N	Y	P	N	Negative	Negative	Negative
838	14	26MAR15	501	N	Y	N	N	Negative	Positive	Negative
842	12	26MAR15	442	N	Y	N	N	Positive	Positive	Negative
852	12	27MAR15	515	N	N	P	N	Positive	Positive	Negative
853	15	27MAR15	528	N	N	N	N	Negative	Positive	Negative
854	7	27MAR15	539	N	N	N	N	Positive	Negative	Negative
855	32	27MAR15	522	N	Y	N	Y	Negative	Negative	Negative
856	7	27MAR15	530	N	Y	N	N	Negative	Negative	Negative
86	13	06MAR15	418	N	N	N	N	Positive	Positive	Positive
865	11	27MAR15	464	N	N	N	N	Positive	Positive	Negative
867	9	27MAR15	523	N	N	N	N	Positive	Positive	Positive
868	7	27MAR15	560	N	Y	P	N	Negative	Negative	Positive
87	19	06MAR15	444	N	Y	Y	N	Negative	Negative	Positive
872	7	27MAR15	534	N	Y	N	N	Positive	Negative	Negative
873	10	27MAR15	538	N	Y	P	N	Negative	Positive	Positive

DATA HAVE NOT BEEN VERIFIED

[1] Did not qualify for BRD re-treatment, not a BRD mortality and not removed for non-BRD reasons

PHASE IV ALL SPECIES STUDY BOVINE
STUDY: A131R-US-13-231
TREATMENT SUCCESS RATE
SUMMARY BY ANIMAL

treatment=T01 test material=DRAXXIN period=First Pull

animal	Infectious Bovine Rhinotracheitis (qPCR-IBR-BHV)	day of BRD	score	temperature (F)	day of removal	brd related	BRD mortality	treatment success [1]
798	Positive	24	1	103.6	24	BRD		NO
799	Positive				18	BRD		NO
80	Negative				12	BRD		NO
800	Negative							YES
805	Negative				21	BRD		NO
806	Positive				19	BRD		NO
807	Negative				14	BRD		NO
809	Negative				17	BRD		NO
814	Negative				33	BRD		NO
815	Negative				14	BRD		NO
818	Negative				13	BRD		NO
819	Negative				17	BRD		NO
824	Negative				19	BRD		NO
829	Negative				15	BRD		NO
83	Negative							YES
832	Negative				21	BRD		NO
834	Negative							YES
836	Negative	28	2	105.2	28	BRD		NO
838	Positive	21	1	103.7	21	BRD		NO
842	Negative							YES
852	Negative				33	BRD		NO
853	Negative							YES
854	Positive							YES
855	Negative							YES
856	Negative							YES
86	Negative				19	BRD		NO
865	Negative				18	BRD		NO
867	Negative				14	BRD		NO
868	Negative	10	4	104.8	10	BRD	YES	NO
87	Negative							YES
872	Negative							YES
873	Negative	12	3	106.9	12	BRD		NO

DATA HAVE NOT BEEN VERIFIED

[1] Did not qualify for BRD re-treatment, not a BRD mortality and not removed for non-BRD reasons

treatment=T01 test material=DRAXXIN period=First Pull

animal	day of study	day 0 date	day 0 weight (LBS)	histophilus somni	mannheimia haemolytica	mycoplasma bovis	pasteurella multocida	Bovine Parainfluenza 3 (qPCR-BPI3)	Bovine Respiratory Syncytial Virus (qPCR-BRSV-PCR)	Bovine Viral Diarrhea (qPCR-BVD-PCR)
874	7	27MAR15	563	N	Y	P	N	Positive	Positive	Negative
876	25	27MAR15	538	N	Y	P	Y	Negative	Negative	Negative
877	9	27MAR15	542	N	Y	N	N	Positive	Positive	Negative
878	18	27MAR15	475	N	N	N	Y	Negative	Negative	Negative
88	18	06MAR15	479	N	Y	Y	N	Positive	Positive	Positive
888	12	27MAR15	483	N	Y	N	N	Positive	Positive	Positive
89	7	06MAR15	479	N	N	N	N	Negative	Negative	Negative
893	26	27MAR15	554	Y	Y	P	N	Negative	Negative	Negative
894	25	27MAR15	538	Y	N	P	N	Negative	Negative	Negative
895	40	27MAR15	486					Negative	Positive	Negative
896	32	27MAR15	582	N	N	N	Y	Negative	Negative	Negative
898	8	27MAR15	519	N	Y	N	N	Negative	Positive	Negative
9	7	06MAR15	526	N	N	N	N	Negative	Negative	Positive
901	10	27MAR15	548	N	N	N	N	Negative	Negative	Positive
904	11	27MAR15	528	N	N	N	N	Negative	Negative	Positive
907	33	27MAR15	575	Y	N	P	Y	Negative	Negative	Negative
912	10	27MAR15	506	N	N	N	N	Negative	Positive	Positive
915	20	27MAR15	441	N	Y	N	N	Negative	Negative	Negative
917	27	27MAR15	525	N	N	N	N	Positive	Positive	Negative
918	12	27MAR15	558	N	N	N	N	Positive	Positive	Negative
922	12	27MAR15	477	N	N	N	N	Positive	Positive	Negative
931	29	27MAR15	510	Y	Y	P	N	Negative	Negative	Negative
932	9	27MAR15	540	N	N	N	N	Negative	Positive	Negative
934	11	27MAR15	518	N	Y	N	N	Positive	Positive	Negative
942	11	28MAR15	449	N	Y	N	Y	Positive	Positive	Negative
944	11	28MAR15	440	N	Y	N	N	Positive	Positive	Positive
945	13	28MAR15	470	N	N	N	N	Negative	Negative	Positive
946	39	28MAR15	539					Negative	Negative	Negative
947	11	28MAR15	477	N	N	N	N	Positive	Positive	Negative
949	11	28MAR15	433	N	N	N	N	Negative	Positive	Positive
950	12	28MAR15	454	N	Y	P	N	Negative	Negative	Negative
951	11	28MAR15	448	N	Y	P	N	Positive	Positive	Negative

DATA HAVE NOT BEEN VERIFIED

[1] Did not qualify for BRD re-treatment, not a BRD mortality and not removed for non-BRD reasons

PHASE IV ALL SPECIES STUDY BOVINE
STUDY: A131R-US-13-231
TREATMENT SUCCESS RATE
SUMMARY BY ANIMAL

treatment=T01 test material=DRAXXIN period=First Pull

animal	Infectious Bovine Rhinotracheitis (qPCR-IBR-BHV)	day of BRD	score	temperature (F)	day of removal	brd related	BRD mortality	treatment success [1]
874	Negative	10	3	105.9	10	BRD		NO
876	Negative	40	1	104.2	40	BRD		NO
877	Negative	19	1	105.3	19	BRD		NO
878	Negative							YES
88	Positive	20	3	101.7	20	BRD		NO
888	Negative	19	2	106.7	19	BRD		NO
89	Negative							YES
893	Negative	33	1	103.6	33	BRD		NO
894	Negative				37	BRD		NO
895	Negative							YES
896	Negative	40	1	103.5	40	BRD		NO
898	Negative							YES
9	Negative				17	BRD		NO
901	Negative							YES
904	Negative				17	BRD		NO
907	Negative							YES
912	Negative				18	BRD		NO
915	Negative	37	1	104.9	37	BRD		NO
917	Negative							YES
918	Negative				21	BRD		NO
922	Negative				14	BRD		NO
931	Negative							YES
932	Negative				23	BRD		NO
934	Negative							YES
942	Positive	13	3	105.8	13	BRD		NO
944	Negative							YES
945	Negative							YES
946	Negative							YES
947	Negative				13	BRD		NO
949	Negative				20	BRD		NO
950	Negative	19	1	104.8	19	BRD		NO
951	Negative	21	3	106.4	21	BRD		NO

DATA HAVE NOT BEEN VERIFIED

[1] Did not qualify for BRD re-treatment, not a BRD mortality and not removed for non-BRD reasons

treatment=T01 test material=DRAXXIN period=First Pull

animal	day of study	day 0 date	day 0 weight (LBS)	histophilus somni	mannheimia haemolytica	mycoplasma bovis	pasteurella multocida	Bovine Parainfluenza 3 (qPCR-BPI3)	Bovine Respiratory Syncytial Virus (qPCR-BRSV-PCR)	Bovine Viral Diarrhea (qPCR-BVD-PCR)
953	7	28MAR15	488	N	Y	N	N	Negative	Positive	Negative
956	12	28MAR15	509	N	Y	P	N	Negative	Negative	Positive
958	10	28MAR15	456	N	N	N	N	Negative	Positive	Positive
962	17	28MAR15	446	N	Y	P	N	Negative	Negative	Negative
964	31	28MAR15	438	N	Y	P	N	Negative	Negative	Negative
97	7	13MAR15	515	N	N	N	N	Positive	Positive	Negative
970	37	28MAR15	465	N	Y		Y	Negative	Positive	Negative
972	17	28MAR15	489	N	Y	P	N	Negative	Negative	Negative
973	14	28MAR15	417	N	Y	N	N	Positive	Positive	Positive
975	9	28MAR15	507	N	N	P	N			
98	10	13MAR15	533	N	Y	Y	N	Positive	Positive	Positive
982	8	28MAR15	513	N	N	N	N	Negative	Negative	Negative
983	16	28MAR15	475	N	Y	N	N	Negative	Negative	Negative
987	21	28MAR15	431	N	Y	N	N	Negative	Positive	Negative
989	8	28MAR15	382	N	Y	N	N	Negative	Positive	Negative
990	16	28MAR15	516	N	N	N	N	Negative	Negative	Negative
996	10	28MAR15	536	N	Y	P	N	Negative	Positive	Positive
999	12	28MAR15	484	N	N	N	N	Negative	Negative	Negative

DATA HAVE NOT BEEN VERIFIED

[1] Did not qualify for BRD re-treatment, not a BRD mortality and not removed for non-BRD reasons

STUDY: A131R-US-13-231
TREATMENT SUCCESS RATE
SUMMARY BY ANIMAL

treatment=T01 test material=DRAXXIN period=First Pull

animal	Infectious Bovine Rhinotracheitis (qPCR-IBR-BHV)	day of BRD	score	temperature (F)	day of removal	brd related	BRD mortality	treatment success [1]
953	Positive	18	2	104.5	18	BRD		NO
956	Negative	15	3	107.4	15	BRD		NO
958	Negative				19	BRD		NO
962	Negative							YES
964	Negative							YES
97	Negative							YES
970	Negative							YES
972	Negative	27	2	104.8	27	BRD		NO
973	Negative	21	2	104.7	21	BRD		NO
975					16	BRD		NO
98	Positive	17	1	105.3	17	BRD		NO
982	Negative				12	BRD		NO
983	Negative							YES
987	Negative							YES
989	Negative	13	3	105.2	13	BRD		NO
990	Negative				18	BRD		NO
996	Negative							YES
999	Negative				19	BRD		NO

DATA HAVE NOT BEEN VERIFIED

[1] Did not qualify for BRD re-treatment, not a BRD mortality and not removed for non-BRD reasons

PHASE IV ALL SPECIES STUDY BOVINE
STUDY: A131R-US-13-231
TREATMENT SUCCESS RATE
SUMMARY OF BACTERIAL ISOLATES

treatment=T01 test material=DRAXXIN

	histophilus somni				ALL
	N		Y		
	n	%	n	%	n
period					
Arrival	1019	99.4	6	0.6	1025
First Pull	369	93.7	25	6.3	394

PHASE IV ALL SPECIES STUDY BOVINE
STUDY: A131R-US-13-231
TREATMENT SUCCESS RATE
SUMMARY OF BACTERIAL ISOLATES

treatment=T01 test material=DRAXXIN

	mannheimia haemolytica				ALL
	N		Y		
	n	%	n	%	n
period					
Arrival	913	89.1	112	10.9	1025
First Pull	260	66.0	134	34.0	394

PHASE IV ALL SPECIES STUDY BOVINE
STUDY: A131R-US-13-231
TREATMENT SUCCESS RATE
SUMMARY OF BACTERIAL ISOLATES

treatment=T01 test material=DRAXXIN

	mycoplasma bovis						ALL
	N		P		Y		
	n	%	n	%	n	%	n
period							
Arrival	1015	99.0	0	0	10	1.0	1025
First Pull	250	63.5	79	20.1	65	16.5	394

DATA HAVE NOT BEEN VERIFIED

PHASE IV ALL SPECIES STUDY BOVINE
STUDY: A131R-US-13-231
TREATMENT SUCCESS RATE
SUMMARY OF BACTERIAL ISOLATES

treatment=T01 test material=DRAXXIN

	pasteurella multocida				ALL
	N		Y		
	n	%	n	%	n
period					
Arrival	918	89.6	107	10.4	1025
First Pull	365	92.6	29	7.4	394

DATA HAVE NOT BEEN VERIFIED

PHASE IV ALL SPECIES STUDY BOVINE
STUDY: A131R-US-13-231
TREATMENT SUCCESS RATE
SUMMARY OF BACTERIAL ISOLATES BY TREATMENT SUCCESS

treatment=T01 test material=DRAXXIN

		treatment success				
		NO		YES		ALL
		n	%	n	%	n
period	histophilus somni					
Arrival	N	418	41.0	601	59.0	1019
	Y	2	33.3	4	66.7	6
	ALL	420	41.0	605	59.0	1025
First Pull	histophilus somni					
	N	201	54.5	168	45.5	369
	Y	15	60.0	10	40.0	25
	ALL	216	54.8	178	45.2	394

PHASE IV ALL SPECIES STUDY BOVINE
STUDY: A131R-US-13-231
TREATMENT SUCCESS RATE
SUMMARY OF BACTERIAL ISOLATES BY TREATMENT SUCCESS

treatment=T01 test material=DRAXXIN

		treatment success				ALL
		NO		YES		ALL
		n	%	n	%	n
period	mannheimia haemolytica					
Arrival	N	365	40.0	548	60.0	913
	Y	55	49.1	57	50.9	112
	ALL	420	41.0	605	59.0	1025
First Pull	mannheimia haemolytica					
	N	148	56.9	112	43.1	260
	Y	68	50.7	66	49.3	134
	ALL	216	54.8	178	45.2	394

PHASE IV ALL SPECIES STUDY BOVINE
STUDY: A131R-US-13-231
TREATMENT SUCCESS RATE
SUMMARY OF BACTERIAL ISOLATES BY TREATMENT SUCCESS

treatment=T01 test material=DRAXXIN

		treatment success				ALL
		NO		YES		
		n	%	n	%	n
period	mycoplasma bovis					
Arrival	N	417	41.1	598	58.9	1015
	Y	3	30.0	7	70.0	10
	ALL	420	41.0	605	59.0	1025
First Pull	mycoplasma bovis					
	N	133	53.2	117	46.8	250
	P	45	57.0	34	43.0	79
	Y	38	58.5	27	41.5	65
	ALL	216	54.8	178	45.2	394

PHASE IV ALL SPECIES STUDY BOVINE
STUDY: A131R-US-13-231
TREATMENT SUCCESS RATE
SUMMARY OF BACTERIAL ISOLATES BY TREATMENT SUCCESS

treatment=T01 test material=DRAXXIN

		treatment success				
		NO		YES		ALL
		n	%	n	%	n
period	pasteurella multocida					
Arrival	N	375	40.8	543	59.2	918
	Y	45	42.1	62	57.9	107
	ALL	420	41.0	605	59.0	1025
First Pull	pasteurella multocida					
	N	200	54.8	165	45.2	365
	Y	16	55.2	13	44.8	29
	ALL	216	54.8	178	45.2	394

PHASE IV ALL SPECIES STUDY BOVINE
STUDY: A131R-US-13-231
TREATMENT SUCCESS RATE
SUMMARY OF VIRAL EXPOSURE AT FIRST PULL

treatment=T01 test material=DRAXXIN

	Bovine Parainfluenza 3 (qPCR-BPI3)				
	Negative		Positive		ALL
	n	%	n	%	n
period					
First Pull	263	65.9	136	34.1	399

PHASE IV ALL SPECIES STUDY BOVINE
STUDY: A131R-US-13-231
TREATMENT SUCCESS RATE
SUMMARY OF VIRAL EXPOSURE AT FIRST PULL

treatment=T01 test material=DRAXXIN

	Bovine Respiratory Syncytial Virus (qPCR-BRSV-PCR)				ALL
	Negative		Positive		
	n	%	n	%	n
period					
First Pull	183	45.9	216	54.1	399

PHASE IV ALL SPECIES STUDY BOVINE
STUDY: A131R-US-13-231
TREATMENT SUCCESS RATE
SUMMARY OF VIRAL EXPOSURE AT FIRST PULL

treatment=T01 test material=DRAXXIN

	Bovine Viral Diarrhea (qPCR-BVD-PCR)				ALL
	Negative		Positive		
	n	%	n	%	n
period					
First Pull	293	73.4	106	26.6	399

PHASE IV ALL SPECIES STUDY BOVINE
STUDY: A131R-US-13-231
TREATMENT SUCCESS RATE
SUMMARY OF VIRAL EXPOSURE AT FIRST PULL

treatment=T01 test material=DRAXXIN

	Infectious Bovine Rhinotracheitis (qPCR-IBR-BHV)				ALL
	Negative		Positive		
	n	%	n	%	n
period					
First Pull	355	89.0	44	11.0	399

DATA HAVE NOT BEEN VERIFIED

PHASE IV ALL SPECIES STUDY BOVINE
STUDY: A131R-US-13-231
TREATMENT SUCCESS RATE
SUMMARY OF VIRAL EXPOSURE BY TREATMENT SUCCESS AT FIRST PULL

treatment=T01 test material=DRAXXIN

		treatment success				ALL
		NO		YES		
		n	%	n	%	n
period	Bovine Parainfluenza 3 (qPCR-BPI3)					
First Pull	Negative	130	49.4	133	50.6	263
	Positive	85	62.5	51	37.5	136
	ALL	215	53.9	184	46.1	399

DATA HAVE NOT BEEN VERIFIED

PHASE IV ALL SPECIES STUDY BOVINE
STUDY: A131R-US-13-231
TREATMENT SUCCESS RATE
SUMMARY OF VIRAL EXPOSURE BY TREATMENT SUCCESS AT FIRST PULL

treatment=T01 test material=DRAXXIN

		treatment success				ALL
		NO		YES		
		n	%	n	%	n
period	Bovine Respiratory Syncytial Virus (qPCR-BRSV-PCR)					
First Pull	Negative	83	45.4	100	54.6	183
	Positive	132	61.1	84	38.9	216
	ALL	215	53.9	184	46.1	399

PHASE IV ALL SPECIES STUDY BOVINE
STUDY: A131R-US-13-231
TREATMENT SUCCESS RATE
SUMMARY OF VIRAL EXPOSURE BY TREATMENT SUCCESS AT FIRST PULL

treatment=T01 test material=DRAXXIN

		treatment success				ALL
		NO		YES		
		n	%	n	%	n
period	Bovine Viral Diarrhea (qPCR-BVD-PCR)					
First Pull	Negative	143	48.8	150	51.2	293
	Positive	72	67.9	34	32.1	106
	ALL	215	53.9	184	46.1	399

PHASE IV ALL SPECIES STUDY BOVINE
STUDY: A131R-US-13-231
TREATMENT SUCCESS RATE
SUMMARY OF VIRAL EXPOSURE BY TREATMENT SUCCESS AT FIRST PULL

treatment=T01 test material=DRAXXIN

		treatment success				ALL
		NO		YES		
		n	%	n	%	n
period	Infectious Bovine Rhinotracheitis (qPCR-IBR-BHV)					
First Pull	Negative	181	51.0	174	49.0	355
	Positive	34	77.3	10	22.7	44
	ALL	215	53.9	184	46.1	399

PHASE IV ALL SPECIES STUDY BOVINE
STUDY: A131R-US-13-231
TREATMENT SUCCESS RATE
SUMMARY OF TIME OF ARRIVAL ON STUDY

treatment=T01 test material=DRAXXIN

	day 0 date																ALL
	06MAR15		13MAR15		20MAR15		23MAR15		25MAR15		26MAR15		27MAR15		28MAR15		
	n	%	n	%	n	%	n	%	n	%	n	%	n	%	n	%	n
**period**																	
**Arrival**	92	9.0	193	18.8	175	17.1	174	17.0	113	11.0	87	8.5	92	9.0	99	9.7	1025
**First Pull**	41	10.2	90	22.4	74	18.4	72	17.9	26	6.5	32	8.0	31	7.7	36	9.0	402

DATA HAVE NOT BEEN VERIFIED

PHASE IV ALL SPECIES STUDY BOVINE
STUDY: A131R-US-13-231
TREATMENT SUCCESS RATE
SUMMARY OF TIME OF ARRIVAL ON STUDY BY TREATMENT SUCCESS

treatment=T01 test material=DRAXXIN

		treatment success				ALL
		NO		YES		
		n	%	n	%	n
period	day 0 date					
Arrival	06MAR15	45	48.9	47	51.1	92
	13MAR15	95	49.2	98	50.8	193
	20MAR15	79	45.1	96	54.9	175
	23MAR15	73	42.0	101	58.0	174
	25MAR15	26	23.0	87	77.0	113
	26MAR15	33	37.9	54	62.1	87
	27MAR15	32	34.8	60	65.2	92
	28MAR15	37	37.4	62	62.6	99
	ALL	420	41.0	605	59.0	1025
First Pull	day 0 date					
	06MAR15	22	53.7	19	46.3	41
	13MAR15	45	50.0	45	50.0	90
	20MAR15	39	52.7	35	47.3	74
	23MAR15	41	56.9	31	43.1	72
	25MAR15	10	38.5	16	61.5	26
	26MAR15	22	68.8	10	31.3	32
	27MAR15	18	58.1	13	41.9	31
	28MAR15	21	58.3	15	41.7	36
	ALL	218	54.2	184	45.8	402

PHASE IV ALL SPECIES STUDY BOVINE
STUDY: A131R-US-13-231
TREATMENT SUCCESS RATE
SUMMARY OF DAY 0 BODY WEIGHT BY TREATMENT SUCCESS

treatment=T01 test material=DRAXXIN

		day 0 weight (LBS)				
		n	mean	std. dev.	minimum	maximum
period	treatment success					
Arrival	NO	420	495.4	49.1	340	633
	YES	605	499.3	56.2	317	642
	All	1025	497.7	53.4	317	642
First Pull	treatment success					
	NO	218	492.8	46.2	372	597
	YES	184	498.7	52.5	340	633
	All	402	495.5	49.2	340	633

PHASE IV ALL SPECIES STUDY BOVINE
STUDY: A131R-US-13-231
TREATMENT SUCCESS RATE
SUMMARY OF MIC BY ANIMAL

treatment=T01 test material=DRAXXIN period=Arrival

animal	day of study	day 0 date	day 0 weight (LBS)	histophilus somni	mannheimia haemolytica	mycoplasma bovis	pasteurella multocida	microorganism	mic	resistant [1]	susceptible [2]
101	0	13MAR15	556	N	Y	N	N	MH	2	NO	YES
1011	0	28MAR15	487	N	Y	N	N	MH	1	NO	YES
1016	0	28MAR15	468	N	Y	N	N	MH	4	NO	YES
1018	0	28MAR15	522	N	N	N	Y	PM	0.25	NO	YES
1027	0	28MAR15	457	N	Y	N	Y	MH	2	NO	YES
			457	N	Y	N	Y	PM	0.5	NO	YES
103	0	13MAR15	577	N	N	N	Y	PM	0.5	NO	YES
1034	0	28MAR15	497	N	Y	N	N	MH	2	NO	YES
11	0	06MAR15	438	N	N	N	Y	PM	0.5	NO	YES
110	0	13MAR15	456	N	N	N	Y	PM	0.5	NO	YES
112	0	13MAR15	551	N	Y	N	N	MH	2	NO	YES
115	0	13MAR15	493	N	Y	N	N	MH	2	NO	YES
117	0	13MAR15	447	N	N	N	Y	PM	0.5	NO	YES
119	0	13MAR15	545	N	N	N	Y	PM	<=0.12	NO	YES
128	0	13MAR15	510	N	Y	N	N	MH	2	NO	YES
131	0	13MAR15	524	N	N	N	Y	PM	0.5	NO	YES
136	0	13MAR15	549	N	N	N	Y	PM	0.5	NO	YES
140	0	13MAR15	476	N	Y	N	N	MH	4	NO	YES
144	0	13MAR15	514	N	Y	N	N	MH	2	NO	YES
149	0	13MAR15	510	N	Y	N	N	MH	2	NO	YES
158	0	13MAR15	512	N	Y	N	N	MH	4	NO	YES
160	0	13MAR15	549	N	Y	N	Y	MH	2	NO	YES
			549	N	Y	N	Y	PM	0.5	NO	YES
161	0	13MAR15	560	N	N	N	Y	PM	0.25	NO	YES
163	0	13MAR15	499	N	Y	N	N	MH	4	NO	YES
164	0	13MAR15	429	N	N	N	Y	PM	0.5	NO	YES
165	0	13MAR15	501	N	Y	Y	N	PMMH	1	NO	YES
166	0	13MAR15	568	N	Y	N	N	MH	1	NO	YES
167	0	13MAR15	569	N	Y	Y	N	MH	1	NO	YES
171	0	13MAR15	524	N	Y	N	N	MH	2	NO	YES
173	0	13MAR15	520	N	Y	Y	N	MH	2	NO	YES
174	0	13MAR15	436	N	N	N	Y	PM	0.5	NO	YES
177	0	13MAR15	507	N	N	N	Y	PM	0.5	NO	YES

DATA HAVE NOT BEEN VERIFIED

[1] MIC < 64 are non-resistant, MIC >= 64 are resistant.
[2] MIC > 16 are non-susceptible, MIC <= 16 are susceptible.
[3] Did not qualify for BRD re-treatment, not a BRD mortality and not removed for non-BRD reasons

treatment=T01 test material=DRAXXIN period=Arrival

animal	day of BRD	score	temperature (F)	day of removal	brd related	BRD mortality	treatment success [3]
101	10	1	105.5	18	BRD		NO
1011	24	1	105.9				NO
1016	37	1	105.5	40	BRD		NO
1018							YES
1027							YES
							YES
103							YES
1034	37	1	103.6				NO
11	10	1	103.5				NO
110	9	1	104.9	17	BRD		NO
112	11	1	105.3				NO
115	7	1	104.6	13	BRD		NO
117	12	1	103.6				NO
119							YES
128	14	1	105.2	23	BRD		NO
131							YES
136	10	1	103.9				NO
140	12	1	104.5	28	BRD		NO
144	10	1	105.1				NO
149	12	1	103.5				NO
158							YES
160	7	1	103.5	14	BRD		NO
	7	1	103.5	14	BRD		NO
161							YES
163	11	1	106.2	18	BRD		NO
164	12	1	103.7	20	BRD		NO
165	8	1	104.7	17	BRD		NO
166	8	1	104.7	16	BRD		NO
167	18	1	106.5				NO
171	10	1	104.0	16	BRD		NO
173							YES
174	12	1	104.0				NO
177	8	1	104.8	30	BRD		NO

DATA HAVE NOT BEEN VERIFIED

[1] MIC < 64 are non-resistant, MIC >= 64 are resistant.
[2] MIC > 16 are non-susceptible, MIC <= 16 are susceptible.
[3] Did not qualify for BRD re-treatment, not a BRD mortality and not removed for non-BRD reasons

treatment=T01 test material=DRAXXIN period=Arrival

animal	day of study	day 0 date	day 0 weight (LBS)	histophilus somni	mannheimia haemolytica	mycoplasma bovis	pasteurella multocida	microorganism	mic	resistant [1]	susceptible [2]
178	0	13MAR15	479	N	Y	N	N	MH	2	NO	YES
179	0	13MAR15	542	N	N	N	Y	PM	0.5	NO	YES
18	0	06MAR15	505	N	Y	N	N	MH	1	NO	YES
183	0	13MAR15	500	N	Y	N	N	MH	2	NO	YES
189	0	13MAR15	508	N	Y	N	N	MH	4	NO	YES
190	0	13MAR15	556	N	Y	N	N	MH	2	NO	YES
198	0	13MAR15	489	N	Y	N	N	MH	1	NO	YES
199	0	13MAR15	554	N	N	N	Y	PM	0.5	NO	YES
2	0	06MAR15	436	N	Y	N	N	MH	2	NO	YES
20	0	06MAR15	438	N	N	N	Y	PM	0.5	NO	YES
203	0	13MAR15	496	N	Y	N	N	MH	4	NO	YES
205	0	13MAR15	475	N	Y	N	N	MH	1	NO	YES
209	0	13MAR15	545	N	Y	N	N	MH	1	NO	YES
211	0	13MAR15	512	N	Y	N	Y	MH	4	NO	YES
			512	N	Y	N	Y	PM	0.25	NO	YES
214	0	13MAR15	475	N	N	N	Y	HSPM	0.25	NO	YES
222	0	13MAR15	490	N	N	Y	Y	PM	2	NO	YES
225	0	13MAR15	494	N	Y	N	N	MH	1	NO	YES
229	0	13MAR15	542	N	N	N	Y	PM	8	NO	YES
230	0	13MAR15	570	N	N	N	Y	PM	0.5	NO	YES
237	0	13MAR15	547	N	Y	N	N	MH	4	NO	YES
238	0	13MAR15	446	N	N	N	Y	PM	0.5	NO	YES
244	0	13MAR15	507	N	Y	N	N	MH	1	NO	YES
248	0	13MAR15	532	N	Y	N	N	MH	1	NO	YES
252	0	13MAR15	457	N	N	N	Y	PM	<=0.12	NO	YES
253	0	13MAR15	503	N	N	N	Y	PM	0.5	NO	YES
263	0	13MAR15	512	N	Y	N	N	MH	2	NO	YES
268	0	13MAR15	560	N	Y	N	N	MH	1	NO	YES
272	0	13MAR15	463	N	N	N	Y	MHPM	0.5	NO	YES
276	0	13MAR15	501	N	N	N	Y	PM	1	NO	YES
28	0	06MAR15	506	N	N	N	Y	PM	0.5	NO	YES
283	0	13MAR15	510	N	Y	N	Y	MH	1	NO	YES
			510	N	Y	N	Y	PM	0.5	NO	YES

DATA HAVE NOT BEEN VERIFIED

[1] MIC < 64 are non-resistant, MIC >= 64 are resistant.
[2] MIC > 16 are non-susceptible, MIC <= 16 are susceptible.
[3] Did not qualify for BRD re-treatment, not a BRD mortality and not removed for non-BRD reasons

PHASE IV ALL SPECIES STUDY BOVINE
STUDY: A131R-US-13-231
TREATMENT SUCCESS RATE
SUMMARY OF MIC BY ANIMAL

treatment=T01 test material=DRAXXIN period=Arrival

animal	day of BRD	score	temperature (F)	day of removal	brd related	BRD mortality	treatment success [3]
178							YES
179	17	1	106.0	35	BRD		NO
18							YES
183							YES
189							YES
190							YES
198							YES
199							YES
2							YES
20	11	1	103.7	24	BRD		NO
203	25	2	104.6	30	BRD		NO
205							YES
209	7	1	103.8	12	BRD		NO
211							YES
							YES
214	11	1	106.3				NO
222	8	1	105.6				NO
225							YES
229							YES
230							YES
237							YES
238	7	1	103.5	17	BRD		NO
244							YES
248	17	2	107.0				NO
252							YES
253							YES
263							YES
268	18	1	104.5				NO
272	33	1	105.2				NO
276							YES
28							YES
283							YES
							YES

DATA HAVE NOT BEEN VERIFIED

[1] MIC < 64 are non-resistant, MIC >= 64 are resistant.
[2] MIC > 16 are non-susceptible, MIC <= 16 are susceptible.
[3] Did not qualify for BRD re-treatment, not a BRD mortality and not removed for non-BRD reasons

PHASE IV ALL SPECIES STUDY BOVINE
STUDY: A131R-US-13-231
TREATMENT SUCCESS RATE
SUMMARY OF MIC BY ANIMAL

treatment=T01 test material=DRAXXIN period=Arrival

animal	day of study	day 0 date	day 0 weight (LBS)	histophilus somni	mannheimia haemolytica	mycoplasma bovis	pasteurella multocida	microorganism	mic	resistant [1]	susceptible [2]
286	0	13MAR15	576	N	N	N	Y	PM	1	NO	YES
287	0	13MAR15	577	N	Y	N	N	MH	1	NO	YES
289	0	13MAR15	406	N	Y	N	N	MH	1	NO	YES
292	0	20MAR15	441	N	Y	N	N	MH	1	NO	YES
294	0	20MAR15	493	N	Y	N	N	MH	2	NO	YES
313	0	20MAR15	423	N	N	N	Y	PM	0.5	NO	YES
314	0	20MAR15	441	N	N	N	Y	MHPM	0.5	NO	YES
320	0	20MAR15	459	N	N	N	Y	PM	0.25	NO	YES
330	0	20MAR15	454	N	N	N	Y	PM	0.5	NO	YES
337	0	20MAR15	457	N	N	N	Y	PM	0.5	NO	YES
339	0	20MAR15	475	N	N	N	Y	PM	0.25	NO	YES
340	0	20MAR15	496	N	N	N	Y	PM	1	NO	YES
352	0	20MAR15	435	N	N	N	Y	PM	0.25	NO	YES
361	0	20MAR15	456	N	N	N	Y	PM	1	NO	YES
363	0	20MAR15	465	N	Y	N	N	MH	2	NO	YES
366	0	20MAR15	434	N	Y	N	N	MH	1	NO	YES
372	0	20MAR15	478	N	N	N	Y	PM	0.5	NO	YES
38	0	06MAR15	464	N	Y	N	N	MH	1	NO	YES
380	0	20MAR15	411	N	N	N	Y	PM	0.5	NO	YES
391	0	20MAR15	597	N	N	N	Y	PM	0.25	NO	YES
397	0	20MAR15	500	N	N	N	Y	PM	0.25	NO	YES
399	0	20MAR15	551	N	N	N	Y	PM	0.5	NO	YES
40	0	06MAR15	506	N	N	N	Y	PM	0.5	NO	YES
405	0	20MAR15	532	N	Y	N	N	MH	1	NO	YES
419	0	20MAR15	511	N	N	N	Y	PM	0.5	NO	YES
425	0	20MAR15	556	N	N	N	Y	PM1	0.5	NO	YES
434	0	20MAR15	500	N	N	N	Y	PM	2	NO	YES
437	0	20MAR15	544	N	Y	N	Y	MH	>64	YES	NO
			544	N	Y	N	Y	PM	16	NO	YES
444	0	20MAR15	494	N	Y	N	N	MH	1	NO	YES
449	0	20MAR15	560	N	Y	N	N	MH	2	NO	YES
452	0	20MAR15	530	N	N	N	Y	PM	16	NO	YES
46	0	06MAR15	470	N	N	N	Y	PM	0.5	NO	YES

DATA HAVE NOT BEEN VERIFIED

[1] MIC < 64 are non-resistant, MIC >= 64 are resistant.
[2] MIC > 16 are non-susceptible, MIC <= 16 are susceptible.
[3] Did not qualify for BRD re-treatment, not a BRD mortality and not removed for non-BRD reasons

PHASE IV ALL SPECIES STUDY BOVINE
STUDY: A131R-US-13-231
TREATMENT SUCCESS RATE
SUMMARY OF MIC BY ANIMAL

treatment=T01 test material=DRAXXIN period=Arrival

animal	day of BRD	score	temperature (F)	day of removal	brd related	BRD mortality	treatment success [3]
286							YES
287	17	1	105.7				NO
289							YES
292	12	1	103.7	24	BRD		NO
294							YES
313							YES
314							YES
320	14	1	107.0				NO
330	8	1	106.1	12	BRD		NO
337							YES
339							YES
340							YES
352							YES
361							YES
363							YES
366	10	1	107.4	17	BRD		NO
372							YES
38	11	1	103.9				NO
380	15	1	106.3	18	BRD		NO
391	14	1	107.0	21	BRD		NO
397							YES
399							YES
40							YES
405							YES
419							YES
425							YES
434							YES
437	11	1	106.1	30	BRD		NO
	11	1	106.1	30	BRD		NO
444							YES
449							YES
452	23	1	106.0	25	BRD		NO
46	7	2	103.5				NO

DATA HAVE NOT BEEN VERIFIED

[1] MIC < 64 are non-resistant, MIC >= 64 are resistant.
[2] MIC > 16 are non-susceptible, MIC <= 16 are susceptible.
[3] Did not qualify for BRD re-treatment, not a BRD mortality and not removed for non-BRD reasons

treatment=T01 test material=DRAXXIN period=Arrival

animal	day of study	day 0 date	day 0 weight (LBS)	histophilus somni	mannheimia haemolytica	mycoplasma bovis	pasteurella multocida	microorganism	mic	resistant [1]	susceptible [2]
464	0	20MAR15	497	N	N	N	Y	PM	1	NO	YES
472	0	23MAR15	514	N	Y	N	N	MH	4	NO	YES
473	0	23MAR15	450	N	Y	N	N	MH	2	NO	YES
474	0	23MAR15	502	N	N	N	Y	PM	<=0.12	NO	YES
476	0	23MAR15	532	N	Y	N	N	MH	4	NO	YES
478	0	23MAR15	555	N	N	N	Y	PM	0.5	NO	YES
481	0	23MAR15	568	N	N	N	Y	PM	0.25	NO	YES
485	0	23MAR15	504	N	Y	N	N	MH	4	NO	YES
487	0	23MAR15	579	N	Y	N	N	MH	1	NO	YES
488	0	23MAR15	430	N	Y	N	N	MH	1	NO	YES
490	0	23MAR15	516	N	N	N	Y	PM	>64	YES	NO
493	0	23MAR15	505	N	Y	N	N	MH	1	NO	YES
495	0	23MAR15	517	N	Y	N	Y	MH	1	NO	YES
			517	N	Y	N	Y	PM	0.5	NO	YES
5	0	06MAR15	472	N	N	N	Y	PM	0.25	NO	YES
500	0	23MAR15	495	N	Y	N	N	MH	4	NO	YES
503	0	23MAR15	512	N	N	N	Y	PM	0.5	NO	YES
505	0	23MAR15	486	N	Y	N	Y	MH	1	NO	YES
			486	N	Y	N	Y	PM	0.5	NO	YES
508	0	23MAR15	485	N	N	N	Y	PM	0.5	NO	YES
512	0	23MAR15	573	N	N	N	Y	PM	0.5	NO	YES
513	0	23MAR15	527	N	N	N	Y	PM	0.25	NO	YES
515	0	23MAR15	540	N	Y	N	N	MH	4	NO	YES
522	0	23MAR15	478	N	N	N	Y	PM	0.25	NO	YES
525	0	23MAR15	537	N	N	N	Y	PM	0.25	NO	YES
531	0	23MAR15	490	N	Y	N	Y	MH	2	NO	YES
			490	N	Y	N	Y	PM	0.5	NO	YES
541	0	23MAR15	502	N	Y	N	N	MH	4	NO	YES
542	0	23MAR15	473	N	Y	N	N	MH	1	NO	YES
543	0	23MAR15	510	N	Y	N	N	MH	1	NO	YES
55	0	06MAR15	470	N	N	N	Y	PM	0.5	NO	YES
551	0	23MAR15	490	N	Y	N	Y	MH	4	NO	YES
			490	N	Y	N	Y	PM	0.25	NO	YES

DATA HAVE NOT BEEN VERIFIED

[1] MIC < 64 are non-resistant, MIC >= 64 are resistant.
[2] MIC > 16 are non-susceptible, MIC <= 16 are susceptible.
[3] Did not qualify for BRD re-treatment, not a BRD mortality and not removed for non-BRD reasons

PHASE IV ALL SPECIES STUDY BOVINE
STUDY: A131R-US-13-231
TREATMENT SUCCESS RATE
SUMMARY OF MIC BY ANIMAL

treatment=T01 test material=DRAXXIN period=Arrival

animal	day of BRD	score	temperature (F)	day of removal	brd related	BRD mortality	treatment success [3]
464							YES
472							YES
473	11	1	104.1	18	BRD		NO
474	12	1	104.9				NO
476	10	1	106.9	15	BRD		NO
478	11	1	103.8	32	BRD		NO
481							YES
485	7	1	104.1	16	BRD		NO
487	36	1	103.5	40	BRD		NO
488	10	1	105.0	22	BRD		NO
490							YES
493	9	1	106.1	17	BRD		NO
495							YES
							YES
5	10	1	103.5	20	BRD		NO
500							YES
503	24	1	106.3				NO
505	7	1	105.3	15	BRD		NO
	7	1	105.3	15	BRD		NO
508	15	1	106.2	18	BRD		NO
512	10	1	103.8				NO
513	21	1	103.6				NO
515							YES
522	9	1	103.8	27	BRD		NO
525							YES
531	15	1	106.2	22	BRD		NO
	15	1	106.2	22	BRD		NO
541	12	1	105.6	19	BRD		NO
542	19	1	103.6				NO
543	11	1	104.6	19	BRD		NO
55	10	1	105.9				NO
551	36	1	105.2				NO
	36	1	105.2				NO

DATA HAVE NOT BEEN VERIFIED

[1] MIC < 64 are non-resistant, MIC >= 64 are resistant.
[2] MIC > 16 are non-susceptible, MIC <= 16 are susceptible.
[3] Did not qualify for BRD re-treatment, not a BRD mortality and not removed for non-BRD reasons

treatment=T01 test material=DRAXXIN period=Arrival

animal	day of study	day 0 date	day 0 weight (LBS)	histophilus somni	mannheimia haemolytica	mycoplasma bovis	pasteurella multocida	microorganism	mic	resistant [1]	susceptible [2]
553	0	23MAR15	543	N	Y	N	N	MH	1	NO	YES
554	0	23MAR15	493	N	N	N	Y	PM1	0.25	NO	YES
556	0	23MAR15	577	N	Y	N	N	MH	2	NO	YES
557	0	23MAR15	542	N	Y	N	N	MH	2	NO	YES
561	0	23MAR15	569	N	Y	N	N	MH	2	NO	YES
563	0	23MAR15	601	N	N	N	Y	PM	0.5	NO	YES
574	0	23MAR15	514	N	N	N	Y	PM	1	NO	YES
576	0	23MAR15	563	N	Y	N	N	MH	2	NO	YES
578	0	23MAR15	505	N	Y	N	N	MH	1	NO	YES
579	0	23MAR15	578	N	Y	N	Y	MH	2	NO	YES
			578	N	Y	N	Y	PM	0.5	NO	YES
58	0	06MAR15	557	N	N	N	Y	PM	1	NO	YES
582	0	23MAR15	554	N	Y	N	N	MH	32	NO	NO
588	0	23MAR15	488	N	Y	N	Y	MH	1	NO	YES
			488	N	Y	N	Y	PM	0.25	NO	YES
59	0	06MAR15	499	N	N	N	Y	PM	1	NO	YES
593	0	23MAR15	580	N	N	N	Y	PM	0.5	NO	YES
595	0	23MAR15	619	N	Y	N	Y	MH	1	NO	YES
			619	N	Y	N	Y	PM	0.5	NO	YES
597	0	23MAR15	535	N	Y	N	Y	MH	1	NO	YES
			535	N	Y	N	Y	PM	0.5	NO	YES
601	0	23MAR15	554	N	N	N	Y	PM	0.5	NO	YES
606	0	23MAR15	572	N	Y	N	Y	MH	32	NO	NO
			572	N	Y	N	Y	PM	0.5	NO	YES
607	0	23MAR15	538	N	N	N	Y	PM	0.5	NO	YES
608	0	23MAR15	540	N	N	N	Y	PM	0.5	NO	YES
609	0	23MAR15	586	N	Y	N	N	MH	1	NO	YES
612	0	23MAR15	549	N	Y	N	N	MH	32	NO	NO
614	0	23MAR15	580	N	Y	N	N	MH	32	NO	NO
616	0	23MAR15	546	N	Y	N	N	MH	2	NO	YES
617	0	23MAR15	600	N	Y	N	N	MH	16	NO	YES
618	0	23MAR15	516	N	Y	N	N	MH	0.5	NO	YES
619	0	23MAR15	588	N	Y	N	N	MH	1	NO	YES

DATA HAVE NOT BEEN VERIFIED

[1] MIC < 64 are non-resistant, MIC >= 64 are resistant.
[2] MIC > 16 are non-susceptible, MIC <= 16 are susceptible.
[3] Did not qualify for BRD re-treatment, not a BRD mortality and not removed for non-BRD reasons

PHASE IV ALL SPECIES STUDY BOVINE
STUDY: A131R-US-13-231
TREATMENT SUCCESS RATE
SUMMARY OF MIC BY ANIMAL

treatment=T01 test material=DRAXXIN period=Arrival

animal	day of BRD	score	temperature (F)	day of removal	brd related	BRD mortality	treatment success [3]
553							YES
554							YES
556	7	1	106.7				NO
557							YES
561	20	1	105.6				NO
563							YES
574							YES
576	7	1	103.8				NO
578							YES
579							YES
							YES
58							YES
582	29	1	106.1				NO
588							YES
							YES
59							YES
593							YES
595	16	1	104.1				NO
	16	1	104.1				NO
597							YES
							YES
601	8	1	103.5	31	BRD		NO
606							YES
							YES
607							YES
608							YES
609							YES
612							YES
614							YES
616							YES
617	28	1	105.1				NO
618	7	1	104.8				NO
619	16	1	104.7	24	BRD		NO

DATA HAVE NOT BEEN VERIFIED

[1] MIC < 64 are non-resistant, MIC >= 64 are resistant.
[2] MIC > 16 are non-susceptible, MIC <= 16 are susceptible.
[3] Did not qualify for BRD re-treatment, not a BRD mortality and not removed for non-BRD reasons

PHASE IV ALL SPECIES STUDY BOVINE
STUDY: A131R-US-13-231
TREATMENT SUCCESS RATE
SUMMARY OF MIC BY ANIMAL

treatment=T01 test material=DRAXXIN period=Arrival

animal	day of study	day 0 date	day 0 weight (LBS)	histophilus somni	mannheimia haemolytica	mycoplasma bovis	pasteurella multocida	microorganism	mic	resistant [1]	susceptible [2]
620	0	23MAR15	547	N	Y	N	N	MH	4	NO	YES
623	0	23MAR15	591	N	Y	N	N	MH	2	NO	YES
624	0	23MAR15	548	N	N	N	Y	PM	0.5	NO	YES
626	0	23MAR15	490	N	N	N	Y	PM	<=0.12	NO	YES
630	0	23MAR15	550	N	Y	N	N	MH	2	NO	YES
631	0	23MAR15	568	N	Y	N	N	MH	32	NO	NO
632	0	23MAR15	558	N	N	N	Y	PM	0.5	NO	YES
634	0	23MAR15	534	N	Y	N	N	MH	32	NO	NO
637	0	23MAR15	526	N	Y	N	N	MH	32	NO	NO
641	0	23MAR15	588	N	Y	N	N	MH	16	NO	YES
65	0	06MAR15	503	N	N	N	Y	PM	0.5	NO	YES
665	0	25MAR15	437	N	N	N	Y	PM	0.5	NO	YES
669	0	25MAR15	405	N	Y	N	N	MH	0.5	NO	YES
68	0	06MAR15	590	N	Y	N	N	MH	2	NO	YES
687	0	25MAR15	418	Y	N	N	Y	PM	0.25	NO	YES
689	0	25MAR15	427	N	N	N	Y	PM	0.5	NO	YES
69	0	06MAR15	497	N	Y	N	N	PMMH	2	NO	YES
694	0	25MAR15	407	N	Y	N	N	MH	1	NO	YES
711	0	25MAR15	430	N	N	N	Y	PM	0.5	NO	YES
716	0	25MAR15	408	N	Y	N	N	MH	1	NO	YES
729	0	25MAR15	371	N	N	N	Y	PM	0.25	NO	YES
733	0	25MAR15	382	N	N	N	Y	PM	0.5	NO	YES
74	0	06MAR15	420	N	N	N	Y	PM	0.5	NO	YES
743	0	25MAR15	424	N	Y	Y	N	MH	2	NO	YES
746	0	25MAR15	432	N	N	N	Y	PM	0.25	NO	YES
765	0	26MAR15	504	N	N	N	Y	PM	1	NO	YES
767	0	26MAR15	508	N	N	N	Y	PM	64	YES	NO
770	0	26MAR15	525	N	Y	N	N	MH	1	NO	YES
794	0	26MAR15	462	N	N	N	Y	PM	0.5	NO	YES
796	0	26MAR15	439	N	Y	N	N	MH	1	NO	YES
808	0	26MAR15	470	N	Y	N	Y	HSPM	0.25	NO	YES
			470	N	Y	N	Y	MH	1	NO	YES
83	0	06MAR15	540	N	Y	N	N	MH	0.5	NO	YES

DATA HAVE NOT BEEN VERIFIED

[1] MIC < 64 are non-resistant, MIC >= 64 are resistant.
[2] MIC > 16 are non-susceptible, MIC <= 16 are susceptible.
[3] Did not qualify for BRD re-treatment, not a BRD mortality and not removed for non-BRD reasons

PHASE IV ALL SPECIES STUDY BOVINE
STUDY: A131R-US-13-231
TREATMENT SUCCESS RATE
SUMMARY OF MIC BY ANIMAL

treatment=T01 test material=DRAXXIN period=Arrival

animal	day of BRD	score	temperature (F)	day of removal	brd related	BRD mortality	treatment success [3]
620							YES
623							YES
624							YES
626	16	1	104.3				NO
630	24	1	104.2				NO
631	7	1	104.6	14	BRD		NO
632							YES
634							YES
637							YES
641							YES
65	11	1	103.6	17	BRD		NO
665	20	1	103.5				NO
669							YES
68							YES
687							YES
689							YES
69	10	1	105.3	18	BRD		NO
694							YES
711							YES
716							YES
729							YES
733							YES
74	7	1	103.9				NO
743							YES
746							YES
765	13	1	106.8				NO
767							YES
770							YES
794							YES
796							YES
808							YES
							YES
83	11	1	103.6				NO

DATA HAVE NOT BEEN VERIFIED

[1] MIC < 64 are non-resistant, MIC >= 64 are resistant.
[2] MIC > 16 are non-susceptible, MIC <= 16 are susceptible.
[3] Did not qualify for BRD re-treatment, not a BRD mortality and not removed for non-BRD reasons

PHASE IV ALL SPECIES STUDY BOVINE
STUDY: A131R-US-13-231
TREATMENT SUCCESS RATE
SUMMARY OF MIC BY ANIMAL

treatment=T01 test material=DRAXXIN period=Arrival

animal	day of study	day 0 date	day 0 weight (LBS)	histophilus somni	mannheimia haemolytica	mycoplasma bovis	pasteurella multocida	microorganism	mic	resistant [1]	susceptible [2]
84	0	06MAR15	460	N	Y	N	Y	MH	1	NO	YES
			460	N	Y	N	Y	PM	0.5	NO	YES
848	0	26MAR15	499	N	N	N	Y	PM	0.5	NO	YES
849	0	27MAR15	566	N	Y	N	N	MH	4	NO	YES
86	0	06MAR15	418	N	Y	N	N	MH	4	NO	YES
87	0	06MAR15	444	N	Y	N	N	PMMH	2	NO	YES
872	0	27MAR15	534	N	N	N	Y	PM	0.5	NO	YES
875	0	27MAR15	519	N	N	N	Y	PM	0.5	NO	YES
879	0	27MAR15	471	N	Y	N	N	MH	>64	YES	NO
89	0	06MAR15	479	N	N	N	Y	PM	1	NO	YES
892	0	27MAR15	546	N	Y	N	N	MH	1	NO	YES
910	0	27MAR15	498	N	Y	N	N	MH	1	NO	YES
912	0	27MAR15	506	N	Y	N	N	MH	1	NO	YES
917	0	27MAR15	525	N	N	N	Y	PM	0.5	NO	YES
94	0	06MAR15	452	N	N	N	Y	PM	0.5	NO	YES
947	0	28MAR15	477	N	Y	N	N	MH	0.5	NO	YES
952	0	28MAR15	462	N	Y	N	N	MH	0.5	NO	YES
961	0	28MAR15	418	N	N	N	Y	PM	0.5	NO	YES
979	0	28MAR15	429	N	Y	N	N	MH	0.5	NO	YES
990	0	28MAR15	516	N	Y	N	N	MH	1	NO	YES
993	0	28MAR15	593	N	N	N	Y	PM	0.25	NO	YES

DATA HAVE NOT BEEN VERIFIED

[1] MIC < 64 are non-resistant, MIC >= 64 are resistant.
[2] MIC > 16 are non-susceptible, MIC <= 16 are susceptible.
[3] Did not qualify for BRD re-treatment, not a BRD mortality and not removed for non-BRD reasons

PHASE IV ALL SPECIES STUDY BOVINE
STUDY: A131R-US-13-231
TREATMENT SUCCESS RATE
SUMMARY OF MIC BY ANIMAL

treatment=T01 test material=DRAXXIN period=Arrival

animal	day of BRD	score	temperature (F)	day of removal	brd related	BRD mortality	treatment success [3]
84	7	2	103.6				NO
	7	2	103.6				NO
848							YES
849							YES
86	13	1	104.9	19	BRD		NO
87	19	1	103.5				NO
872	7	1	104.2				NO
875							YES
879							YES
89	7	2	102.4				NO
892							YES
910							YES
912	10	1	104.3	18	BRD		NO
917	27	1	103.5				NO
94							YES
947	11	1	104.7	13	BRD		NO
952							YES
961							YES
979							YES
990	16	1	106.9	18	BRD		NO
993	7	1	104.4				NO

DATA HAVE NOT BEEN VERIFIED

[1] MIC < 64 are non-resistant, MIC >= 64 are resistant.
[2] MIC > 16 are non-susceptible, MIC <= 16 are susceptible.
[3] Did not qualify for BRD re-treatment, not a BRD mortality and not removed for non-BRD reasons

PHASE IV ALL SPECIES STUDY BOVINE
STUDY: A131R-US-13-231
TREATMENT SUCCESS RATE
SUMMARY OF MIC BY ANIMAL

treatment=T01 test material=DRAXXIN period=First Pull

animal	day of study	day 0 date	day 0 weight (LBS)	histophilus somni	mannheimia haemolytica	mycoplasma bovis	pasteurella multocida	Bovine Parainfluenza 3 (qPCR-BPI3)	Bovine Respiratory Syncytial Virus (qPCR-BRSV-PCR)	Bovine Viral Diarrhea (qPCR-BVD-PCR)	Infectious Bovine Rhinotracheitis (qPCR-IBR-BHV)
1004	19	28MAR15	540	N	Y	P	N	Negative	Negative	Negative	Negative
1006	12	28MAR15	450	N	Y	N	N	Negative	Positive	Negative	Negative
101	10	13MAR15	556	N	Y	N	N	Positive	Positive	Positive	Positive
1011	24	28MAR15	487	N	Y	P	N	Negative	Negative	Negative	Negative
1016	37	28MAR15	468	N	Y		Y	Negative	Positive	Negative	Negative
			468	N	Y		Y	Negative	Positive	Negative	Negative
1030	11	28MAR15	533	N	Y	N	N	Positive	Positive	Positive	Negative
1032	14	28MAR15	470	N	Y	N	N	Negative	Positive	Negative	Negative
1033	16	28MAR15	487	N	Y	N	N	Negative	Negative	Negative	Negative
104	12	13MAR15	548	N	Y	Y	N	Negative	Negative	Negative	Negative
112	11	13MAR15	551	N	N	Y	Y	Positive	Negative	Positive	Negative
130	11	13MAR15	590	N	Y	Y	N	Positive	Negative	Positive	Negative
133	11	13MAR15	532	N	Y	Y	N	Positive	Positive	Positive	Positive
141	11	13MAR15	539	N	Y	Y	N	Positive	Negative	Positive	Negative
142	12	13MAR15	462	N	Y	Y	N	Negative	Negative	Positive	Negative
159	11	13MAR15	541	Y	Y	Y	N	Negative	Positive	Positive	Positive
163	11	13MAR15	499	N	Y	Y	N	Positive	Positive	Positive	Negative
164	12	13MAR15	429	N	N	N	Y	Positive	Positive	Negative	Negative
167	18	13MAR15	569	N	Y	Y	N	Negative	Positive	Negative	Negative
179	17	13MAR15	542	N	Y	Y	N	Negative	Negative	Negative	Negative
181	17	13MAR15	478	N	Y	Y	N	Negative	Negative	Negative	Negative
184	7	13MAR15	517	N	Y	Y	N	Positive	Positive	Negative	Negative
200	40	13MAR15	477	Y	Y	N	Y	Negative	Negative	Negative	Negative
			477	Y	Y	N	Y	Negative	Negative	Negative	Negative
201	7	13MAR15	470	N	Y	N	N	Positive	Positive	Negative	Negative
202	18	13MAR15	575	N	Y	Y	N	Positive	Positive	Negative	Negative
212	13	13MAR15	483	N	Y	Y	N	Negative	Negative	Negative	Negative
221	19	13MAR15	486	N	Y	N	N	Positive	Negative	Negative	Negative
222	8	13MAR15	490	N	Y	Y	N	Positive	Positive	Negative	Negative
232	14	13MAR15	508	N	Y	Y	N	Positive	Positive	Negative	Negative
248	17	13MAR15	532	N	Y	N	N	Negative	Positive	Negative	Negative

DATA HAVE NOT BEEN VERIFIED

[1] MIC < 64 are non-resistant, MIC >= 64 are resistant.
[2] MIC > 16 are non-susceptible, MIC <= 16 are susceptible.
[3] Did not qualify for BRD re-treatment, not a BRD mortality and not removed for non-BRD reasons

PHASE IV ALL SPECIES STUDY BOVINE
STUDY: A131R-US-13-231
TREATMENT SUCCESS RATE
SUMMARY OF MIC BY ANIMAL

treatment=T01 test material=DRAXXIN period=First Pull

animal	microorganism	mic	resistant [1]	susceptible [2]	day of BRD	score	temperature (F)	day of removal	brd related	BRD mortality	treatment success [3]
1004	MH	>64	YES	NO							YES
1006	MH	>64	YES	NO							YES
101	MH	>64	YES	NO	18	1	103.5	18	BRD		NO
1011	MH	>64	YES	NO							YES
1016	MH	>64	YES	NO	40	3	102.4	40	BRD		NO
	PM	>64	YES	NO	40	3	102.4	40	BRD		NO
1030	MH	>64	YES	NO	19	1	105.5	19	BRD		NO
1032	MH	>64	YES	NO							YES
1033	MH	>64	YES	NO							YES
104	MH	>64	YES	NO							YES
112	PM	32	NO	NO							YES
130	MH	>64	YES	NO	13	3	105.9	13	BRD		NO
133	MH	>64	YES	NO							YES
141	MH	>64	YES	NO	40	1	106.0	40	BRD		NO
142	MH	>64	YES	NO							YES
159	MH	>64	YES	NO	36	2	105.1	36	BRD		NO
163	MH	>64	YES	NO	18	2	106.2	18	BRD		NO
164	PM	0.5	NO	YES	19	2		20	BRD		NO
167	MH	>64	YES	NO							YES
179	MH	>64	YES	NO	35	1	103.5	35	BRD		NO
181	MH	>64	YES	NO							YES
184	MH	2	NO	YES	13	3	107.3	13	BRD		NO
200	MH	>64	YES	NO							YES
	PM	>64	YES	NO							YES
201	MH	>64	YES	NO							YES
202	MH	>64	YES	NO							YES
212	MH	>64	YES	NO							YES
221	MH	>64	YES	NO							YES
222	MH	>64	YES	NO							YES
232	MH	>64	YES	NO	20	3	105.0	20	BRD		NO
248	MH	>64	YES	NO							YES

DATA HAVE NOT BEEN VERIFIED

[1] MIC < 64 are non-resistant, MIC >= 64 are resistant.
[2] MIC > 16 are non-susceptible, MIC <= 16 are susceptible.
[3] Did not qualify for BRD re-treatment, not a BRD mortality and not removed for non-BRD reasons

PHASE IV ALL SPECIES STUDY BOVINE
STUDY: A131R-US-13-231
TREATMENT SUCCESS RATE
SUMMARY OF MIC BY ANIMAL

treatment=T01 test material=DRAXXIN period=First Pull

animal	day of study	day 0 date	day 0 weight (LBS)	histophilus somni	mannheimia haemolytica	mycoplasma bovis	pasteurella multocida	Bovine Parainfluenza 3 (qPCR-BPI3)	Bovine Respiratory Syncytial Virus (qPCR-BRSV-PCR)	Bovine Viral Diarrhea (qPCR-BVD-PCR)	Infectious Bovine Rhinotracheitis (qPCR-IBR-BHV)
250	25	13MAR15	500	N	Y	P	Y	Negative	Negative	Negative	Negative
			500	N	Y	P	Y	Negative	Negative	Negative	Negative
255	18	13MAR15	450	N	Y	N	N	Positive	Positive	Negative	Negative
258	18	13MAR15	480	N	N	Y	Y	Negative	Positive	Negative	Negative
264	21	13MAR15	533	Y	Y	P	N	Negative	Negative	Negative	Negative
265	20	13MAR15	435	N	N	N	Y	Positive	Negative	Negative	Negative
266	21	13MAR15	552	N	Y	N	N	Negative	Negative	Negative	Positive
268	18	13MAR15	560	N	Y	Y	N	Negative	Negative	Negative	Negative
271	21	13MAR15	527	N	Y	P	N	Negative	Negative	Negative	Negative
277	19	13MAR15	480	N	Y	N	N	Negative	Positive	Negative	Negative
291	13	20MAR15	480	N	Y	P	N	Positive	Positive	Positive	Positive
311	12	20MAR15	405	N	Y	Y	N	Negative	Positive	Negative	Negative
312	18	20MAR15	484	N	Y	N	N	Negative	Negative	Negative	Negative
315	25	20MAR15	425	N	N	P	Y	Negative	Negative	Negative	Negative
318	25	20MAR15	455	N	Y	P	Y	Negative	Negative	Negative	Negative
			455	N	Y	P	Y	Negative	Negative	Negative	Negative
323	14	20MAR15	430	N	Y	P	N	Negative	Positive	Negative	Negative
329	15	20MAR15	465	N	Y	N	N	Negative	Positive	Negative	Negative
343	18	20MAR15	557	N	Y	N	N	Negative	Positive	Negative	Negative
346	13	20MAR15	422	N	N	P	Y	Negative	Negative	Negative	Positive
349	16	20MAR15	441	N	Y	P	N	Positive	Positive	Negative	Negative
365	19	20MAR15	457	N	Y	N	N	Negative	Positive	Negative	Negative
367	14	20MAR15	474	N	Y	N	N	Negative	Positive	Negative	Negative
374	13	20MAR15	475	N	Y	P	N	Positive	Negative	Negative	Negative
377	23	20MAR15	436	N	Y	P	N	Negative	Negative	Negative	Negative
388	15	20MAR15	471	N	Y	P	N	Negative	Negative	Negative	Negative
390	16	20MAR15	573	N	Y	P	N	Negative	Positive	Negative	Negative
391	14	20MAR15	597	N	Y	P	N	Negative	Positive	Negative	Negative
406	17	20MAR15	533	N	Y	P	N	Positive	Negative	Negative	Negative
411	12	20MAR15	538	N	Y	Y	N	Negative	Positive	Negative	Negative
415	10	20MAR15	521	N	Y	N	N	Positive	Positive	Negative	Negative

DATA HAVE NOT BEEN VERIFIED

[1] MIC < 64 are non-resistant, MIC >= 64 are resistant.
[2] MIC > 16 are non-susceptible, MIC <= 16 are susceptible.
[3] Did not qualify for BRD re-treatment, not a BRD mortality and not removed for non-BRD reasons

PHASE IV ALL SPECIES STUDY BOVINE
STUDY: A131R-US-13-231
TREATMENT SUCCESS RATE
SUMMARY OF MIC BY ANIMAL

treatment=T01 test material=DRAXXIN period=First Pull

animal	microorganism	mic	resistant [1]	susceptible [2]	day of BRD	score	temperature (F)	day of removal	brd related	BRD mortality	treatment success [3]
250	MH	>64	YES	NO							YES
	PM	16	NO	YES							YES
255	MH	>64	YES	NO							YES
258	PM	16	NO	YES	28	1	103.7	28	BRD		NO
264	MH	>64	YES	NO	28	3	105.4	28	BRD		NO
265	PM	32	NO	NO	30	1	104.0	30	BRD		NO
266	MH	>64	YES	NO							YES
268	MH	>64	YES	NO							YES
271	MH	>64	YES	NO	28	2	105.1	28	BRD		NO
277	MH	>64	YES	NO							YES
291	MH	>64	YES	NO	20	2	104.2	20	BRD		NO
311	MH	>64	YES	NO	19	2	105.7	19	BRD		NO
312	MH	>64	YES	NO							YES
315	PM	>64	YES	NO							YES
318	MH	>64	YES	NO	32	1	103.8	32	BRD		NO
	PM	1	NO	YES	32	1	103.8	32	BRD		NO
323	MH	>64	YES	NO							YES
329	MH	>64	YES	NO							YES
343	MH	>64	YES	NO	26	1	104.5	26	BRD		NO
346	PM	>64	YES	NO	23	3	105.8	23	BRD		NO
349	MH	>64	YES	NO	27	1	104.8	27	BRD		NO
365	MH	>64	YES	NO	27	1	105.3	27	BRD		NO
367	MH	>64	YES	NO	17	3	105.6	17	BRD		NO
374	MH	>64	YES	NO	15	3	104.5	15	BRD	YES	NO
377	MH	>64	YES	NO							YES
388	MH	>64	YES	NO	19	3	104.5	19	BRD		NO
390	MH	>64	YES	NO							YES
391	MH	>64	YES	NO	21	2	106.0	21	BRD		NO
406	MH	>64	YES	NO							YES
411	MH	>64	YES	NO							YES
415	MH	>64	YES	NO	17	1	104.8	17	BRD		NO

DATA HAVE NOT BEEN VERIFIED

[1] MIC < 64 are non-resistant, MIC >= 64 are resistant.
[2] MIC > 16 are non-susceptible, MIC <= 16 are susceptible.
[3] Did not qualify for BRD re-treatment, not a BRD mortality and not removed for non-BRD reasons

treatment=T01 test material=DRAXXIN period=First Pull

animal	day of study	day 0 date	day 0 weight (LBS)	histophilus somni	mannheimia haemolytica	mycoplasma bovis	pasteurella multocida	Bovine Parainfluenza 3 (qPCR-BPI3)	Bovine Respiratory Syncytial Virus (qPCR-BRSV-PCR)	Bovine Viral Diarrhea (qPCR-BVD-PCR)	Infectious Bovine Rhinotracheitis (qPCR-IBR-BHV)
416	13	20MAR15	489	N	Y	P	N	Negative	Positive	Negative	Positive
422	15	20MAR15	514	N	Y	P	N	Negative	Positive	Negative	Positive
428	14	20MAR15	515	N	N	P	Y	Negative	Positive	Negative	Negative
433	12	20MAR15	546	N	N	Y	Y	Negative	Positive	Negative	Positive
445	12	20MAR15	551	N	Y	N	N	Positive	Positive	Negative	Negative
452	23	20MAR15	530	Y	N	N	Y	Negative	Negative	Negative	Negative
454	11	20MAR15	523	Y	Y	Y	N	Negative	Positive	Positive	Positive
467	21	20MAR15	560	N	N	P	Y	Negative	Negative	Negative	Negative
471	16	23MAR15	529	N	Y	N	N	Positive	Positive	Negative	Negative
478	11	23MAR15	555	N	Y	P	N	Positive	Negative	Negative	Negative
484	14	23MAR15	537	N	Y	N	N	Positive	Positive	Negative	Negative
486	15	23MAR15	484	N	Y	N	Y	Negative	Positive	Negative	Negative
			484	N	Y	N	Y	Negative	Positive	Negative	Negative
487	36	23MAR15	579	N	N	P	Y	Negative	Negative	Negative	Negative
491	7	23MAR15	482	N	Y	N	N	Positive	Negative	Negative	Negative
492	10	23MAR15	537	N	Y	P	N	Positive	Negative	Negative	Positive
505	7	23MAR15	486	N	Y	Y	N	Negative	Negative	Negative	Negative
506	15	23MAR15	539	N	Y	P	N	Positive	Positive	Negative	Negative
509	8	23MAR15	457	N	Y	Y	N	Positive	Positive	Negative	Negative
51	10	06MAR15	514	N	Y	Y	N	Negative	Negative	Negative	Negative
510	16	23MAR15	575	Y	Y	P	N	Positive	Positive	Negative	Negative
512	10	23MAR15	573	N	N	P	Y	Negative	Negative	Positive	Positive
518	10	23MAR15	518	N	Y	N	N	Negative	Positive	Negative	Negative
520	7	23MAR15	545	N	N	Y	Y	Negative	Positive	Negative	Negative
524	15	23MAR15	500	N	Y	P	N	Positive	Negative	Negative	Negative
531	15	23MAR15	490	N	Y	P	N	Negative	Negative	Negative	Negative
537	17	23MAR15	491	N	Y	P	N	Negative	Positive	Negative	Negative
541	12	23MAR15	502	N	Y	N	N	Negative	Negative	Negative	Negative
542	19	23MAR15	473	N	Y	P	Y	Negative	Negative	Negative	Positive
			473	N	Y	P	Y	Negative	Negative	Negative	Positive
547	36	23MAR15	433	N	Y	N	N	Negative	Negative	Negative	Negative

DATA HAVE NOT BEEN VERIFIED

[1] MIC < 64 are non-resistant, MIC >= 64 are resistant.
[2] MIC > 16 are non-susceptible, MIC <= 16 are susceptible.
[3] Did not qualify for BRD re-treatment, not a BRD mortality and not removed for non-BRD reasons

treatment=T01 test material=DRAXXIN period=First Pull

animal	microorganism	mic	resistant [1]	susceptible [2]	day of BRD	score	temperature (F)	day of removal	brd related	BRD mortality	treatment success [3]
416	MH	>64	YES	NO							YES
422	MH	>64	YES	NO	18	3	104.5	18	BRD		NO
428	PM	16	NO	YES							YES
433	PM	32	NO	NO	15	3	104.4	15	BRD		NO
445	MH	>64	YES	NO							YES
452	PM	16	NO	YES	25	3	106.2	25	BRD		NO
454	MH	>64	YES	NO	18	1	103.8	18	BRD		NO
467	PM	8	NO	YES							YES
471	MH	>64	YES	NO	18	3	104.1	18	BRD		NO
478	MH	>64	YES	NO	32	2	103.8	32	BRD		NO
484	MH	>64	YES	NO							YES
486	HSPM	>64	YES	NO	18	3	107.4	18	BRD		NO
	MH	>64	YES	NO	18	3	107.4	18	BRD		NO
487	PM	>64	YES	NO	40	3	105.1	40	BRD		NO
491	MH	>64	YES	NO	22	1	105.0	22	BRD		NO
492	MH	>64	YES	NO	14	3	104.1	14	BRD		NO
505	MH	>64	YES	NO	15	1	103.9	15	BRD		NO
506	MH	>64	YES	NO							YES
509	MH	>64	YES	NO	21	2	102.5	21	BRD		NO
51	MH	4	NO	YES	17	2	104.6	17	BRD		NO
510	MH	>64	YES	NO	24	1	104.0	24	BRD		NO
512	PM	>64	YES	NO							YES
518	MH	>64	YES	NO							YES
520	PM	>64	YES	NO	15	1	104.6	15	BRD		NO
524	MH	>64	YES	NO	22	1	105.0	22	BRD		NO
531	MH	>64	YES	NO	22	1	105.8	22	BRD		NO
537	MH	>64	YES	NO	22	3	106.7	22	BRD		NO
541	MH	>64	YES	NO	19	1	104.6	19	BRD		NO
542	MH	>64	YES	NO							YES
	PM	>64	YES	NO							YES
547	MH	>64	YES	NO							YES

DATA HAVE NOT BEEN VERIFIED

[1] MIC < 64 are non-resistant, MIC >= 64 are resistant.
[2] MIC > 16 are non-susceptible, MIC <= 16 are susceptible.
[3] Did not qualify for BRD re-treatment, not a BRD mortality and not removed for non-BRD reasons

PHASE IV ALL SPECIES STUDY BOVINE
STUDY: A131R-US-13-231
TREATMENT SUCCESS RATE
SUMMARY OF MIC BY ANIMAL

treatment=T01 test material=DRAXXIN period=First Pull

animal	day of study	day 0 date	day 0 weight (LBS)	histophilus somni	mannheimia haemolytica	mycoplasma bovis	pasteurella multocida	Bovine Parainfluenza 3 (qPCR-BPI3)	Bovine Respiratory Syncytial Virus (qPCR-BRSV-PCR)	Bovine Viral Diarrhea (qPCR-BVD-PCR)	Infectious Bovine Rhinotracheitis (qPCR-IBR-BHV)
549	14	23MAR15	469	Y	N	P	Y	Negative	Negative	Negative	Negative
564	11	23MAR15	481	N	N	N	Y	Negative	Positive	Negative	Negative
618	7	23MAR15	516	N	Y	Y	N	Negative	Positive	Positive	Negative
62	24	06MAR15	444	N	Y	Y	N	Negative	Negative	Negative	Negative
625	11	23MAR15	497	N	Y	N	N	Negative	Positive	Negative	Negative
644	15	25MAR15	402	N	Y	N	N	Negative	Negative	Negative	Negative
645	14	25MAR15	429	N	N	P	Y	Negative	Positive	Negative	Negative
665	20	25MAR15	437	N	Y	P	N	Negative	Negative	Negative	Negative
675	16	25MAR15	455	N	Y	N	N	Negative	Positive	Negative	Negative
693	15	25MAR15	433	N	Y	P	Y	Negative	Negative	Negative	Negative
			433	N	Y	P	Y	Negative	Negative	Negative	Negative
706	15	25MAR15	424	N	Y	N	N	Negative	Negative	Negative	Negative
708	16	25MAR15	407	N	Y	N	N	Negative	Positive	Negative	Negative
712	22	25MAR15	355	N	Y	N	N	Negative	Negative	Negative	Negative
713	15	25MAR15	409	N	Y	N	N	Negative	Positive	Negative	Negative
721	15	25MAR15	412	N	Y	N	N	Negative	Negative	Negative	Negative
737	14	25MAR15	340	N	Y	N	N	Positive	Positive	Negative	Negative
745	14	25MAR15	450	N	Y	N	N	Positive	Positive	Negative	Negative
752	14	25MAR15	434	N	Y	P	N	Positive	Positive	Negative	Negative
753	9	25MAR15	404	N	Y	N	N	Negative	Negative	Negative	Negative
754	18	25MAR15	349	N	Y	P	N	Negative	Negative	Negative	Negative
766	18	26MAR15	485	N	Y	N	N	Positive	Negative	Negative	Negative
772	18	26MAR15	558	Y	Y	P	N	Positive	Negative	Negative	Negative
773	10	26MAR15	454	N	Y	N	N	Negative	Negative	Positive	Positive
775	8	26MAR15	466	N	Y	N	N	Negative	Positive	Positive	Negative
783	14	26MAR15	505	N	Y	P	N	Negative	Positive	Negative	Negative
786	17	26MAR15	499	N	Y	P	N	Negative	Negative	Negative	Negative
793	9	26MAR15	532	N	Y	N	N	Negative	Positive	Positive	Negative
795	16	26MAR15	535	N	Y	N	N	Negative	Negative	Positive	Negative
798	8	26MAR15	545	N	Y	N	Y	Negative	Positive	Negative	Positive
			545	N	Y	N	Y	Negative	Positive	Negative	Positive

DATA HAVE NOT BEEN VERIFIED

[1] MIC < 64 are non-resistant, MIC >= 64 are resistant.
[2] MIC > 16 are non-susceptible, MIC <= 16 are susceptible.
[3] Did not qualify for BRD re-treatment, not a BRD mortality and not removed for non-BRD reasons

PHASE IV ALL SPECIES STUDY BOVINE
STUDY: A131R-US-13-231
TREATMENT SUCCESS RATE
SUMMARY OF MIC BY ANIMAL

treatment=T01 test material=DRAXXIN period=First Pull

animal	microorganism	mic	resistant [1]	susceptible [2]	day of BRD	score	temperature (F)	day of removal	brd related	BRD mortality	treatment success [3]
549	PM	>64	YES	NO	29	1	104.5	29	BRD		NO
564	PM	0.5	NO	YES							YES
618	MH	32	NO	NO							YES
62	MH	>64	YES	NO							YES
625	MH	32	NO	NO							YES
644	MH	>64	YES	NO	22	1	103.7	22	BRD		NO
645	PM	0.5	NO	YES							YES
665	MH	>64	YES	NO							YES
675	MH	>64	YES	NO							YES
693	MH	>64	YES	NO	22	2	103.7	22	BRD		NO
	PM	0.5	NO	YES	22	2	103.7	22	BRD		NO
706	MH	>64	YES	NO	22	3	106.8	22	BRD		NO
708	MH	>64	YES	NO							YES
712	MH	>64	YES	NO							YES
713	MH	>64	YES	NO	22	1	105.4	22	BRD		NO
721	MH	>64	YES	NO	22	2	105.0	22	BRD		NO
737	MH	>64	YES	NO							YES
745	MH	>64	YES	NO							YES
752	MH	>64	YES	NO	24	1	103.7	24	BRD		NO
753	MH	>64	YES	NO							YES
754	MH	>64	YES	NO							YES
766	MH	>64	YES	NO							YES
772	MH	32	NO	NO	25	2	103.8	25	BRD		NO
773	MH	>64	YES	NO							YES
775	MH	>64	YES	NO	12	3	106.0	12	BRD		NO
783	MH	>64	YES	NO	21	1	106.0	21	BRD		NO
786	MH	>64	YES	NO	33	2	103.2	33	BRD		NO
793	MH	>64	YES	NO							YES
795	MH	>64	YES	NO							YES
798	MH	>64	YES	NO	24	1	103.6	24	BRD		NO
	PM	32	NO	NO	24	1	103.6	24	BRD		NO

DATA HAVE NOT BEEN VERIFIED

[1] MIC < 64 are non-resistant, MIC >= 64 are resistant.
[2] MIC > 16 are non-susceptible, MIC <= 16 are susceptible.
[3] Did not qualify for BRD re-treatment, not a BRD mortality and not removed for non-BRD reasons

treatment=T01 test material=DRAXXIN period=First Pull

animal	day of study	day 0 date	day 0 weight (LBS)	histophilus somni	mannheimia haemolytica	mycoplasma bovis	pasteurella multocida	Bovine Parainfluenza 3 (qPCR-BPI3)	Bovine Respiratory Syncytial Virus (qPCR-BRSV-PCR)	Bovine Viral Diarrhea (qPCR-BVD-PCR)	Infectious Bovine Rhinotracheitis (qPCR-IBR-BHV)
8	14	06MAR15	522	Y	N	N	Y	Positive	Positive	Positive	Negative
800	12	26MAR15	510	N	Y	N	N	Positive	Negative	Negative	Negative
836	19	26MAR15	443	N	Y	P	N	Negative	Negative	Negative	Negative
838	14	26MAR15	501	N	Y	N	N	Negative	Positive	Negative	Positive
842	12	26MAR15	442	N	Y	N	N	Positive	Positive	Negative	Negative
855	32	27MAR15	522	N	Y	N	Y	Negative	Negative	Negative	Negative
			522	N	Y	N	Y	Negative	Negative	Negative	Negative
856	7	27MAR15	530	N	Y	N	N	Negative	Negative	Negative	Negative
868	7	27MAR15	560	N	Y	P	N	Negative	Negative	Positive	Negative
87	19	06MAR15	444	N	Y	Y	N	Negative	Negative	Positive	Negative
872	7	27MAR15	534	N	Y	N	N	Positive	Negative	Negative	Negative
873	10	27MAR15	538	N	Y	P	N	Negative	Positive	Positive	Negative
874	7	27MAR15	563	N	Y	P	N	Positive	Positive	Negative	Negative
876	25	27MAR15	538	N	Y	P	Y	Negative	Negative	Negative	Negative
			538	N	Y	P	Y	Negative	Negative	Negative	Negative
877	9	27MAR15	542	N	Y	N	N	Positive	Positive	Negative	Negative
878	18	27MAR15	475	N	N	N	Y	Negative	Negative	Negative	Negative
88	18	06MAR15	479	N	Y	Y	N	Positive	Positive	Positive	Positive
888	12	27MAR15	483	N	Y	N	N	Positive	Positive	Positive	Negative
893	26	27MAR15	554	Y	Y	P	N	Negative	Negative	Negative	Negative
896	32	27MAR15	582	N	N	N	Y	Negative	Negative	Negative	Negative
898	8	27MAR15	519	N	Y	N	N	Negative	Positive	Negative	Negative
907	33	27MAR15	575	Y	N	P	Y	Negative	Negative	Negative	Negative
915	20	27MAR15	441	N	Y	N	N	Negative	Negative	Negative	Negative
931	29	27MAR15	510	Y	Y	P	N	Negative	Negative	Negative	Negative
934	11	27MAR15	518	N	Y	N	N	Positive	Positive	Negative	Negative
942	11	28MAR15	449	N	Y	N	Y	Positive	Positive	Negative	Positive
			449	N	Y	N	Y	Positive	Positive	Negative	Positive
944	11	28MAR15	440	N	Y	N	N	Positive	Positive	Positive	Negative
950	12	28MAR15	454	N	Y	P	N	Negative	Negative	Negative	Negative
951	11	28MAR15	448	N	Y	P	N	Positive	Positive	Negative	Negative

DATA HAVE NOT BEEN VERIFIED

[1] MIC < 64 are non-resistant, MIC >= 64 are resistant.
[2] MIC > 16 are non-susceptible, MIC <= 16 are susceptible.
[3] Did not qualify for BRD re-treatment, not a BRD mortality and not removed for non-BRD reasons

treatment=T01 test material=DRAXXIN period=First Pull

animal	microorganism	mic	resistant [1]	susceptible [2]	day of BRD	score	temperature (F)	day of removal	brd related	BRD mortality	treatment success [3]
8	PM	>64	YES	NO							YES
800	MH	>64	YES	NO							YES
836	MH	>64	YES	NO	28	2	105.2	28	BRD		NO
838	MH	>64	YES	NO	21	1	103.7	21	BRD		NO
842	MH	>64	YES	NO							YES
855	MH	>64	YES	NO							YES
	PM	>64	YES	NO							YES
856	MH	>64	YES	NO							YES
868	MH	>64	YES	NO	10	4	104.8	10	BRD	YES	NO
87	MH	>64	YES	NO							YES
872	MH	>64	YES	NO							YES
873	MH	>64	YES	NO	12	3	106.9	12	BRD		NO
874	MH	>64	YES	NO	10	3	105.9	10	BRD		NO
876	MH	>64	YES	NO	40	1	104.2	40	BRD		NO
	PM	>64	YES	NO	40	1	104.2	40	BRD		NO
877	MH	>64	YES	NO	19	1	105.3	19	BRD		NO
878	PM	>64	YES	NO							YES
88	MH	4	NO	YES	20	3	101.7	20	BRD		NO
888	MH	>64	YES	NO	19	2	106.7	19	BRD		NO
893	MH	>64	YES	NO	33	1	103.6	33	BRD		NO
896	PM	>64	YES	NO	40	1	103.5	40	BRD		NO
898	MH	>64	YES	NO							YES
907	PM	16	NO	YES							YES
915	MH	>64	YES	NO	37	1	104.9	37	BRD		NO
931	MH	>64	YES	NO							YES
934	MH	>64	YES	NO							YES
942	MH	>64	YES	NO	13	3	105.8	13	BRD		NO
	PM	>64	YES	NO	13	3	105.8	13	BRD		NO
944	MH	>64	YES	NO							YES
950	MH	>64	YES	NO	19	1	104.8	19	BRD		NO
951	MH	>64	YES	NO	21	3	106.4	21	BRD		NO

DATA HAVE NOT BEEN VERIFIED

[1] MIC < 64 are non-resistant, MIC >= 64 are resistant.
[2] MIC > 16 are non-susceptible, MIC <= 16 are susceptible.
[3] Did not qualify for BRD re-treatment, not a BRD mortality and not removed for non-BRD reasons

PHASE IV ALL SPECIES STUDY BOVINE
STUDY: A131R-US-13-231
TREATMENT SUCCESS RATE
SUMMARY OF MIC BY ANIMAL

treatment=T01 test material=DRAXXIN period=First Pull

animal	day of study	day 0 date	day 0 weight (LBS)	histophilus somni	mannheimia haemolytica	mycoplasma bovis	pasteurella multocida	Bovine Parainfluenza 3 (qPCR-BPI3)	Bovine Respiratory Syncytial Virus (qPCR-BRSV-PCR)	Bovine Viral Diarrhea (qPCR-BVD-PCR)	Infectious Bovine Rhinotracheitis (qPCR-IBR-BHV)
953	7	28MAR15	488	N	Y	N	N	Negative	Positive	Negative	Positive
956	12	28MAR15	509	N	Y	P	N	Negative	Negative	Positive	Negative
962	17	28MAR15	446	N	Y	P	N	Negative	Negative	Negative	Negative
964	31	28MAR15	438	N	Y	P	N	Negative	Negative	Negative	Negative
970	37	28MAR15	465	N	Y		Y	Negative	Positive	Negative	Negative
			465	N	Y		Y	Negative	Positive	Negative	Negative
972	17	28MAR15	489	N	Y	P	N	Negative	Negative	Negative	Negative
973	14	28MAR15	417	N	Y	N	N	Positive	Positive	Positive	Negative
98	10	13MAR15	533	N	Y	Y	N	Positive	Positive	Positive	Positive
983	16	28MAR15	475	N	Y	N	N	Negative	Negative	Negative	Negative
987	21	28MAR15	431	N	Y	N	N	Negative	Positive	Negative	Negative
989	8	28MAR15	382	N	Y	N	N	Negative	Positive	Negative	Negative
996	10	28MAR15	536	N	Y	P	N	Negative	Positive	Positive	Negative

DATA HAVE NOT BEEN VERIFIED

[1] MIC < 64 are non-resistant, MIC >= 64 are resistant.
[2] MIC > 16 are non-susceptible, MIC <= 16 are susceptible.
[3] Did not qualify for BRD re-treatment, not a BRD mortality and not removed for non-BRD reasons

PHASE IV ALL SPECIES STUDY BOVINE
STUDY: A131R-US-13-231
TREATMENT SUCCESS RATE
SUMMARY OF MIC BY ANIMAL

treatment=T01 test material=DRAXXIN period=First Pull

animal	microorganism	mic	resistant [1]	susceptible [2]	day of BRD	score	temperature (F)	day of removal	brd related	BRD mortality	treatment success [3]
953	MH	>64	YES	NO	18	2	104.5	18	BRD		NO
956	MH	>64	YES	NO	15	3	107.4	15	BRD		NO
962	MH	>64	YES	NO							YES
964	MH	>64	YES	NO							YES
970	MH	>64	YES	NO							YES
	PM	0.25	NO	YES							YES
972	MH	>64	YES	NO	27	2	104.8	27	BRD		NO
973	MH	>64	YES	NO	21	2	104.7	21	BRD		NO
98	MH	>64	YES	NO	17	1	105.3	17	BRD		NO
983	MH	>64	YES	NO							YES
987	MH	>64	YES	NO							YES
989	MH	>64	YES	NO	13	3	105.2	13	BRD		NO
996	MH	>64	YES	NO							YES

DATA HAVE NOT BEEN VERIFIED

[1] MIC < 64 are non-resistant, MIC >= 64 are resistant.
[2] MIC > 16 are non-susceptible, MIC <= 16 are susceptible.
[3] Did not qualify for BRD re-treatment, not a BRD mortality and not removed for non-BRD reasons

PHASE IV ALL SPECIES STUDY BOVINE
STUDY: A131R-US-13-231
TREATMENT SUCCESS RATE
SUMMARY BY RESISTANCE OF BACTERIAL ISOLATES AND PERIOD

treatment=T01 test material=DRAXXIN

			treatment success				ALL
			NO		YES		
			n	%	n	%	n
period	microorganism	resistant					
Arrival	MH	NO	54	49.1	56	50.9	110
		YES	1	50.0	1	50.0	2
		ALL	55	49.1	57	50.9	112
	PM	resistant					
		NO	45	42.9	60	57.1	105
		YES	0	0	2	100.0	2
		ALL	45	42.1	62	57.9	107
First Pull	MH	resistant					
		NO	4	66.7	2	33.3	6
		YES	65	50.0	65	50.0	130
		ALL	69	50.7	67	49.3	136
	PM	resistant					
		NO	8	50.0	8	50.0	16
		YES	9	56.3	7	43.8	16
		ALL	17	53.1	15	46.9	32

PHASE IV ALL SPECIES STUDY BOVINE
STUDY: A131R-US-13-231
TREATMENT SUCCESS RATE
SUMMARY BY RESISTANCE OF BACTERIAL ISOLATES AND PERIOD
SENSITIVITY, SPECIFICITY, POSITIVE PREDICTIVE VALUE AND NEGATIVE PREDICTIVE VALUE

treatment=T01 test material=DRAXXIN

period	microorganism	sensitivity	specificity	positive predictive value	negative predictive value
Arrival	MH	0.018	0.982	0.500	0.509
	PM	0.000	0.968	0.000	0.571
First Pull	MH	0.942	0.030	0.500	0.333
	PM	0.529	0.533	0.563	0.500

treatment=T01 test material=DRAXXIN

			microorganism									
			MH					PM				
			treatment success				ALL	treatment success				ALL
			NO		YES			NO		YES		
			n	%	n	%	n	n	%	n	%	n
period	day 0 date	resistant										
Arrival	06MAR15	NO	6	66.7	3	33.3	9	9	64.3	5	35.7	14
		ALL	6	66.7	3	33.3	9	9	64.3	5	35.7	14
	13MAR15	resistant										
		NO	18	54.5	15	45.5	33	12	48.0	13	52.0	25
		ALL	18	54.5	15	45.5	33	12	48.0	13	52.0	25
	20MAR15	resistant										
		NO	2	28.6	5	71.4	7	6	30.0	14	70.0	20
		YES	1	100.0	0	0	1	0	0	0	0	0
		ALL	3	37.5	5	62.5	8	6	30.0	14	70.0	20
	23MAR15	resistant										
		NO	22	52.4	20	47.6	42	13	46.4	15	53.6	28
		YES	0	0	0	0	0	0	0	1	100.0	1
		ALL	22	52.4	20	47.6	42	13	44.8	16	55.2	29
	25MAR15	resistant										
		NO	0	0	4	100.0	4	1	14.3	6	85.7	7
		ALL	0	0	4	100.0	4	1	14.3	6	85.7	7
	26MAR15	resistant										
		NO	0	0	3	100.0	3	1	25.0	3	75.0	4
		YES	0	0	0	0	0	0	0	1	100.0	1
		ALL	0	0	3	100.0	3	1	20.0	4	80.0	5
	27MAR15	resistant										
		NO	1	25.0	3	75.0	4	2	66.7	1	33.3	3
		YES	0	0	1	100.0	1	0	0	0	0	0

(Continued)

PHASE IV ALL SPECIES STUDY BOVINE
STUDY: A131R-US-13-231
TREATMENT SUCCESS RATE
SUMMARY BY TIME OF ARRIVAL ON STUDY, RESISTANCE OF BACTERIAL ISOLATES AND PERIOD

treatment=T01 test material=DRAXXIN

			microorganism									
			MH					PM				
			treatment success				ALL	treatment success				ALL
			NO		YES			NO		YES		
			n	%	n	%	n	n	%	n	%	n
period	day 0 date	resistant										
Arrival	27MAR15	ALL	1	20.0	4	80.0	5	2	66.7	1	33.3	3
	28MAR15	resistant										
		NO	5	62.5	3	37.5	8	1	25.0	3	75.0	4
		ALL	5	62.5	3	37.5	8	1	25.0	3	75.0	4
First Pull	06MAR15	resistant										
		NO	2	100.0	0	0	2	0	0	0	0	0
		YES	0	0	2	100.0	2	0	0	1	100.0	1
		ALL	2	50.0	2	50.0	4	0	0	1	100.0	1
	13MAR15	resistant										
		NO	1	100.0	0	0	1	3	60.0	2	40.0	5
		YES	10	37.0	17	63.0	27	0	0	1	100.0	1
		ALL	11	39.3	17	60.7	28	3	50.0	3	50.0	6
	20MAR15	resistant										
		NO	0	0	0	0	0	3	60.0	2	40.0	5
		YES	13	59.1	9	40.9	22	1	50.0	1	50.0	2
		ALL	13	59.1	9	40.9	22	4	57.1	3	42.9	7
	23MAR15	resistant										
		NO	0	0	2	100.0	2	0	0	1	100.0	1
		YES	12	70.6	5	29.4	17	4	66.7	2	33.3	6
		ALL	12	63.2	7	36.8	19	4	57.1	3	42.9	7
	25MAR15	resistant										
		NO	0	0	0	0	0	1	50.0	1	50.0	2
		YES	6	42.9	8	57.1	14	0	0	0	0	0

(Continued)

PHASE IV ALL SPECIES STUDY BOVINE
STUDY: A131R-US-13-231
TREATMENT SUCCESS RATE
SUMMARY BY TIME OF ARRIVAL ON STUDY, RESISTANCE OF BACTERIAL ISOLATES AND PERIOD

treatment=T01 test material=DRAXXIN

			microorganism									
			MH					PM				
			treatment success				ALL	treatment success				ALL
			NO		YES			NO		YES		
			n	%	n	%	n	n	%	n	%	n
period	day 0 date	resistant										
First Pull	25MAR15	ALL	6	42.9	8	57.1	14	1	50.0	1	50.0	2
	26MAR15	resistant										
		NO	1	100.0	0	0	1	1	100.0	0	0	1
		YES	6	50.0	6	50.0	12	0	0	0	0	0
		ALL	7	53.8	6	46.2	13	1	100.0	0	0	1
	27MAR15	resistant										
		NO	0	0	0	0	0	0	0	1	100.0	1
		YES	8	57.1	6	42.9	14	2	50.0	2	50.0	4
		ALL	8	57.1	6	42.9	14	2	40.0	3	60.0	5
	28MAR15	resistant										
		NO	0	0	0	0	0	0	0	1	100.0	1
		YES	10	45.5	12	54.5	22	2	100.0	0	0	2
		ALL	10	45.5	12	54.5	22	2	66.7	1	33.3	3

PHASE IV ALL SPECIES STUDY BOVINE
STUDY: A131R-US-13-231
TREATMENT SUCCESS RATE
SUMMARY BY VIRAL EXPOSURE AND RESISTANCE OF BACTERIAL ISOLATES AT FIRST PULL

treatment=T01 test material=DRAXXIN

			microorganism									
			MH					PM				
			treatment success				ALL	treatment success				ALL
			NO		YES			NO		YES		
			n	%	n	%	n	n	%	n	%	n
period	Bovine Parainfluenza 3 (qPCR-BPI3)	resistant										
First Pull	Negative	NO	1	33.3	2	66.7	3	6	46.2	7	53.8	13
		YES	40	46.0	47	54.0	87	8	57.1	6	42.9	14
		ALL	41	45.6	49	54.4	90	14	51.9	13	48.1	27
	Positive	resistant										
		NO	3	100.0	0	0	3	2	66.7	1	33.3	3
		YES	25	58.1	18	41.9	43	1	50.0	1	50.0	2
		ALL	28	60.9	18	39.1	46	3	60.0	2	40.0	5

DATA HAVE NOT BEEN VERIFIED

TREATMENT SUCCESS RATE

SUMMARY BY VIRAL EXPOSURE AND RESISTANCE OF BACTERIAL ISOLATES AT FIRST PULL

treatment=T01 test material=DRAXXIN

			microorganism									
			MH					PM				
			treatment success				ALL	treatment success				ALL
			NO		YES			NO		YES		
			n	%	n	%	n	n	%	n	%	n
period	Bovine Respiratory Syncytial Virus (qPCR-BRSV-PCR)	resistant										
First Pull	Negative	NO	2	100.0	0	0	2	4	50.0	4	50.0	8
		YES	28	45.2	34	54.8	62	5	45.5	6	54.5	11
		ALL	30	46.9	34	53.1	64	9	47.4	10	52.6	19
	Positive	resistant										
		NO	2	50.0	2	50.0	4	4	50.0	4	50.0	8
		YES	37	54.4	31	45.6	68	4	80.0	1	20.0	5
		ALL	39	54.2	33	45.8	72	8	61.5	5	38.5	13

PHASE IV ALL SPECIES STUDY BOVINE
STUDY: A131R-US-13-231
TREATMENT SUCCESS RATE
SUMMARY BY VIRAL EXPOSURE AND RESISTANCE OF BACTERIAL ISOLATES AT FIRST PULL

treatment=T01 test material=DRAXXIN

			microorganism									
			MH					PM				
			treatment success				ALL	treatment success				ALL
			NO		YES			NO		YES		
			n	%	n	%	n	n	%	n	%	n
period	Bovine Viral Diarrhea (qPCR-BVD-PCR)	resistant										
First Pull	Negative	NO	3	75.0	1	25.0	4	8	53.3	7	46.7	15
		YES	50	46.7	57	53.3	107	9	64.3	5	35.7	14
		ALL	53	47.7	58	52.3	111	17	58.6	12	41.4	29
	Positive	resistant										
		NO	1	50.0	1	50.0	2	0	0	1	100.0	1
		YES	15	65.2	8	34.8	23	0	0	2	100.0	2
		ALL	16	64.0	9	36.0	25	0	0	3	100.0	3

STUDY: A131R-US-13-231
TREATMENT SUCCESS RATE
SUMMARY BY VIRAL EXPOSURE AND RESISTANCE OF BACTERIAL ISOLATES AT FIRST PULL

treatment=T01 test material=DRAXXIN

			microorganism									
			MH					PM				
			treatment success				ALL	treatment success				ALL
			NO		YES			NO		YES		
			n	%	n	%	n	n	%	n	%	n
period	Infectious Bovine Rhinotracheitis (qPCR-IBR-BHV)	resistant										
First Pull	Negative	NO	3	60.0	2	40.0	5	6	42.9	8	57.1	14
		YES	54	47.4	60	52.6	114	7	58.3	5	41.7	12
		ALL	57	47.9	62	52.1	119	13	50.0	13	50.0	26
	Positive	resistant										
		NO	1	100.0	0	0	1	2	100.0	0	0	2
		YES	11	68.8	5	31.3	16	2	50.0	2	50.0	4
		ALL	12	70.6	5	29.4	17	4	66.7	2	33.3	6

PHASE IV ALL SPECIES STUDY BOVINE
STUDY: A131R-US-13-231
TREATMENT SUCCESS RATE
SUMMARY BY SUSCEPTIBILITY OF BACTERIAL ISOLATES AND PERIOD

treatment=T01 test material=DRAXXIN

			treatment success				
			NO		YES		ALL
			n	%	n	%	n
period	microorganism	susceptible					
Arrival	MH	NO	3	33.3	6	66.7	9
		YES	52	50.5	51	49.5	103
		ALL	55	49.1	57	50.9	112
	PM	susceptible					
		NO	0	0	2	100.0	2
		YES	45	42.9	60	57.1	105
		ALL	45	42.1	62	57.9	107
First Pull	MH	susceptible					
		NO	66	49.6	67	50.4	133
		YES	3	100.0	0	0	3
		ALL	69	50.7	67	49.3	136
	PM	susceptible					
		NO	12	60.0	8	40.0	20
		YES	5	41.7	7	58.3	12
		ALL	17	53.1	15	46.9	32

PHASE IV ALL SPECIES STUDY BOVINE
STUDY: A131R-US-13-231
TREATMENT SUCCESS RATE
SUMMARY BY SUSCEPTIBILITY OF BACTERIAL ISOLATES AND PERIOD
SENSITIVITY, SPECIFICITY, POSITIVE PREDICTIVE VALUE AND NEGATIVE PREDICTIVE VALUE

treatment=T01 test material=DRAXXIN

period	microorganism	sensitivity	specificity	positive predictive value	negative predictive value
Arrival	MH	0.895	0.055	0.495	0.333
	PM	0.968	0.000	0.571	0.000
First Pull	MH	0.000	0.957	0.000	0.496
	PM	0.467	0.706	0.583	0.600

DATA HAVE NOT BEEN VERIFIED

treatment=T01 test material=DRAXXIN

			microorganism									
			MH					PM				
			treatment success				ALL	treatment success				ALL
			NO		YES			NO		YES		
			n	%	n	%	n	n	%	n	%	n
period	day 0 date	susceptible										
Arrival	06MAR15	YES	6	66.7	3	33.3	9	9	64.3	5	35.7	14
		ALL	6	66.7	3	33.3	9	9	64.3	5	35.7	14
	13MAR15	susceptible										
		YES	18	54.5	15	45.5	33	12	48.0	13	52.0	25
		ALL	18	54.5	15	45.5	33	12	48.0	13	52.0	25
	20MAR15	susceptible										
		NO	1	100.0	0	0	1	0	0	0	0	0
		YES	2	28.6	5	71.4	7	6	30.0	14	70.0	20
		ALL	3	37.5	5	62.5	8	6	30.0	14	70.0	20
	23MAR15	susceptible										
		NO	2	28.6	5	71.4	7	0	0	1	100.0	1
		YES	20	57.1	15	42.9	35	13	46.4	15	53.6	28
		ALL	22	52.4	20	47.6	42	13	44.8	16	55.2	29
	25MAR15	susceptible										
		YES	0	0	4	100.0	4	1	14.3	6	85.7	7
		ALL	0	0	4	100.0	4	1	14.3	6	85.7	7
	26MAR15	susceptible										
		NO	0	0	0	0	0	0	0	1	100.0	1
		YES	0	0	3	100.0	3	1	25.0	3	75.0	4
		ALL	0	0	3	100.0	3	1	20.0	4	80.0	5
	27MAR15	susceptible										
		NO	0	0	1	100.0	1	0	0	0	0	0
		YES	1	25.0	3	75.0	4	2	66.7	1	33.3	3

(Continued)

SUMMARY BY TIME OF ARRIVAL ON STUDY, SUSCEPTIBILITY OF BACTERIAL ISOLATES AND PERIOD

treatment=T01 test material=DRAXXIN

			microorganism									
			MH					PM				
			treatment success				ALL	treatment success				ALL
			NO		YES			NO		YES		
			n	%	n	%	n	n	%	n	%	n
**period**	**day 0 date**	**susceptible**										
**Arrival**	**27MAR15**	**ALL**	1	20.0	4	80.0	5	2	66.7	1	33.3	3
	**28MAR15**	**susceptible**										
		**YES**	5	62.5	3	37.5	8	1	25.0	3	75.0	4
		**ALL**	5	62.5	3	37.5	8	1	25.0	3	75.0	4
**First Pull**	**06MAR15**	**susceptible**										
		**NO**	0	0	2	100.0	2	0	0	1	100.0	1
		**YES**	2	100.0	0	0	2	0	0	0	0	0
		**ALL**	2	50.0	2	50.0	4	0	0	1	100.0	1
	**13MAR15**	**susceptible**										
		**NO**	10	37.0	17	63.0	27	1	33.3	2	66.7	3
		**YES**	1	100.0	0	0	1	2	66.7	1	33.3	3
		**ALL**	11	39.3	17	60.7	28	3	50.0	3	50.0	6
	**20MAR15**	**susceptible**										
		**NO**	13	59.1	9	40.9	22	2	66.7	1	33.3	3
		**YES**	0	0	0	0	0	2	50.0	2	50.0	4
		**ALL**	13	59.1	9	40.9	22	4	57.1	3	42.9	7
	**23MAR15**	**susceptible**										
		**NO**	12	63.2	7	36.8	19	4	66.7	2	33.3	6
		**YES**	0	0	0	0	0	0	0	1	100.0	1
		**ALL**	12	63.2	7	36.8	19	4	57.1	3	42.9	7
	**25MAR15**	**susceptible**										
		**NO**	6	42.9	8	57.1	14	0	0	0	0	0
		**YES**	0	0	0	0	0	1	50.0	1	50.0	2

(Continued)

PHASE IV ALL SPECIES STUDY BOVINE
STUDY: A131R-US-13-231
TREATMENT SUCCESS RATE
SUMMARY BY TIME OF ARRIVAL ON STUDY, SUSCEPTIBILITY OF BACTERIAL ISOLATES AND PERIOD

treatment=T01 test material=DRAXXIN

			microorganism									
			MH					PM				
			treatment success				ALL	treatment success				ALL
			NO		YES			NO		YES		
			n	%	n	%	n	n	%	n	%	n
period	day 0 date	susceptible										
First Pull	25MAR15	ALL	6	42.9	8	57.1	14	1	50.0	1	50.0	2
	26MAR15	susceptible										
		NO	7	53.8	6	46.2	13	1	100.0	0	0	1
		ALL	7	53.8	6	46.2	13	1	100.0	0	0	1
	27MAR15	susceptible										
		NO	8	57.1	6	42.9	14	2	50.0	2	50.0	4
		YES	0	0	0	0	0	0	0	1	100.0	1
		ALL	8	57.1	6	42.9	14	2	40.0	3	60.0	5
	28MAR15	susceptible										
		NO	10	45.5	12	54.5	22	2	100.0	0	0	2
		YES	0	0	0	0	0	0	0	1	100.0	1
		ALL	10	45.5	12	54.5	22	2	66.7	1	33.3	3

PHASE IV ALL SPECIES STUDY BOVINE
STUDY: A131R-US-13-231
TREATMENT SUCCESS RATE
SUMMARY BY VIRAL EXPOSURE AND SUSCEPTIBILITY OF BACTERIAL ISOLATES AT FIRST PULL

treatment=T01 test material=DRAXXIN

			microorganism									
			MH					PM				
			treatment success				ALL	treatment success				ALL
			NO		YES			NO		YES		
			n	%	n	%	n	n	%	n	%	n
period	Bovine Parainfluenza 3 (qPCR-BPI3)	susceptible										
First Pull	Negative	NO	40	44.9	49	55.1	89	10	62.5	6	37.5	16
		YES	1	100.0	0	0	1	4	36.4	7	63.6	11
		ALL	41	45.6	49	54.4	90	14	51.9	13	48.1	27
	Positive	susceptible										
		NO	26	59.1	18	40.9	44	2	50.0	2	50.0	4
		YES	2	100.0	0	0	2	1	100.0	0	0	1
		ALL	28	60.9	18	39.1	46	3	60.0	2	40.0	5

PHASE IV ALL SPECIES STUDY BOVINE
STUDY: A131R-US-13-231
TREATMENT SUCCESS RATE
SUMMARY BY VIRAL EXPOSURE AND SUSCEPTIBILITY OF BACTERIAL ISOLATES AT FIRST PULL

treatment=T01 test material=DRAXXIN

			microorganism									
			MH					PM				
			treatment success				ALL	treatment success				ALL
			NO		YES			NO		YES		
			n	%	n	%	n	n	%	n	%	n
period	Bovine Respiratory Syncytial Virus (qPCR-BRSV-PCR)	susceptible										
First Pull	Negative	NO	29	46.0	34	54.0	63	6	46.2	7	53.8	13
		YES	1	100.0	0	0	1	3	50.0	3	50.0	6
		ALL	30	46.9	34	53.1	64	9	47.4	10	52.6	19
	Positive	susceptible										
		NO	37	52.9	33	47.1	70	6	85.7	1	14.3	7
		YES	2	100.0	0	0	2	2	33.3	4	66.7	6
		ALL	39	54.2	33	45.8	72	8	61.5	5	38.5	13

treatment=T01 test material=DRAXXIN

			microorganism									
			MH					PM				
			treatment success				ALL	treatment success				ALL
			NO		YES			NO		YES		
			n	%	n	%	n	n	%	n	%	n
period	Bovine Viral Diarrhea (qPCR-BVD-PCR)	susceptible										
First Pull	Negative	NO	51	46.8	58	53.2	109	12	70.6	5	29.4	17
		YES	2	100.0	0	0	2	5	41.7	7	58.3	12
		ALL	53	47.7	58	52.3	111	17	58.6	12	41.4	29
	Positive	susceptible										
		NO	15	62.5	9	37.5	24	0	0	3	100.0	3
		YES	1	100.0	0	0	1	0	0	0	0	0
		ALL	16	64.0	9	36.0	25	0	0	3	100.0	3

SUMMARY BY VIRAL EXPOSURE AND SUSCEPTIBILITY OF BACTERIAL ISOLATES AT FIRST PULL

treatment=T01 test material=DRAXXIN

			microorganism									
			MH					PM				
			treatment success				ALL	treatment success				ALL
			NO		YES			NO		YES		
			n	%	n	%	n	n	%	n	%	n
period	Infectious Bovine Rhinotracheitis (qPCR-IBR-BHV)	susceptible										
First Pull	Negative	NO	55	47.0	62	53.0	117	8	57.1	6	42.9	14
		YES	2	100.0	0	0	2	5	41.7	7	58.3	12
		ALL	57	47.9	62	52.1	119	13	50.0	13	50.0	26
	Positive	susceptible										
		NO	11	68.8	5	31.3	16	4	66.7	2	33.3	6
		YES	1	100.0	0	0	1	0	0	0	0	0
		ALL	12	70.6	5	29.4	17	4	66.7	2	33.3	6

PHASE IV ALL SPECIES STUDY BOVINE
STUDY: A131R-US-13-231
TREATMENT SUCCESS RATE
SUMMARY OF MIC BY PERIOD

treatment=T01 test material=DRAXXIN

		mic																					ALL
		<=0.12		0.25		0.5		1		2		4		8		16		32		64		>64		
		n	%	n	%	n	%	n	%	n	%	n	%	n	%	n	%	n	%	n	%	n	%	n
**period**	**microorganism**																							
**Arrival**	**PM**	4	3.7	22	20.6	64	59.8	10	9.3	2	1.9	0	0	1	0.9	2	1.9	0	0	1	0.9	1	0.9	107
	**MH**	0	0	0	0	6	5.4	44	39.3	33	29.5	18	16.1	0	0	2	1.8	7	6.3	0	0	2	1.8	112
**First Pull**	**PM**	0	0	1	3.1	4	12.5	1	3.1	0	0	0	0	1	3.1	5	15.6	4	12.5	0	0	16	50.0	32
	**MH**	0	0	0	0	0	0	0	0	1	0.7	2	1.5	0	0	0	0	3	2.2	0	0	130	95.6	136
